# Possible risk factors of opaque bubble layer and its effect on high-order aberrations after small incision Lenticule extraction

**DOI:** 10.3389/fmed.2023.1156677

**Published:** 2023-12-20

**Authors:** Shan Yang, Heng Wang, Zhengyu Chen, Ying Li, Youxin Chen, Qin Long

**Affiliations:** Department of Ophthalmology, Peking Union Medical College Hospital, Chinese Academy of Medical Sciences and Peking Union Medical College, Beijing, China

**Keywords:** opaque bubble layer, high-order aberration, small incision lenticule extraction, short term, visual quality

## Abstract

**Purpose:**

To evaluate the possible risk factors of opaque bubble layer (OBL) formation in small incision lenticule extraction (SMILE) surgery and its effects on visual quality.

**Methods:**

Fifty-six eyes from 28 patients were included in this study. The preoperative parameters and intraoperative designs were recorded. Corneal high-order aberrations (HOAs), point spread function (PSF), and modulation transfer function (MTF) were measured using iTrace at pre-operation, 1 week, 1 month, and 3 months after SMILE. Generalized Estimating Equation and Linear Mixed Effects Model were employed for statistical analysis.

**Results:**

The mean OBL area in SMILE surgery was 2.75% ± 1.25%. The patients were divided into groups based on whether the OBL was greater than the mean group or less than the mean group. Compared to the group with a smaller OBL area, the group with the larger OBL area had steeper corneal curvature and thinner cap thickness, the OBL area was positively correlated with the preoperative keratometry (r = 0.21, *p* = 0.04) and preoperative spherical value (r = 0.47, *p* = 0.01). The group with the larger OBL area induced more corneal SA and trefoil at 1 week postoperatively, but the difference was not significant at 1 month and 3 months postoperatively.

**Conclusion:**

A steep corneal curvature, thin cap thickness, and high preoperative spherical value are possible risk factors for OBL formation in SMILE surgery. The OBL increased the ocular and corneal HOAs postoperatively for a short period (1 week), while it did not affect the long-term outcomes.

## Introduction

Small incision lenticular extraction (SMILE) has been widely used for the surgical treatment of ametropia (1, 2). Compared to laser *in situ* keratomileusis (LASIK), SMILE, which creates a refractive lenticule within the corneal stroma using a femtosecond laser, showed better corneal mechanical stability, less denervation and dry eye symptoms postoperatively (3). Opaque bubble layer (OBL) is a common intraoperative complication of corneal refractive surgeries involving a femtosecond laser (4–6). The OBL may interfere with flap separation and delay the recovery of visual acuity. It may cause flap tearing and perforation in some severe cases, leading to vison-threatening complications (7). Therefore, it’s of great significance to reduce the development of OBL during SMILE surgery.

A femtosecond laser emits a light pulse of 10^−15^ s at a wavelength of 1,043 nm, causing photodisruption and creating a gas bubble (carbon dioxide and water) to separate the corneal lamellae (8, 9). Occasionally, an OBL may develop when the detained gas bubbles accumulate in the stroma layer resulting in tissue opacity. Previous reports found that OBL development was associated with corneal curvature, corneal thickness, docking technique, and flap size during FS-LASIK surgery (10–12). Several studies also showed that a thicker cornea and thinner lenticule are possible risk factors of OBL during SMILE.

Although OBL formation may cause difficulty during SMILE surgery, OBL does not affect the long-term visual acuity and refraction (13). Visual perception is a complex psychophysical process, and the visual system adapts to changes in the environment, as well as the type of astigmatism, thereby influencing visual quality (14, 15). High-order aberrations are kind of refractive error, but they cannot be corrected with sphere or cylinder. Previous study has found that combination correction of coma and astigmatism can improve retinal image quality over the condition with the same amount of astigmatism alone, especially in eyes with no natural astigmatism (16, 17). However, no study has been conducted to evaluate the effect of OBL on visual quality after SMILE surgery. The purpose of this study is to evaluate OBL risk factors and the effect of OBL on high-order aberrations and visual quality parameters during SMILE surgery.

### Patients and methods

This prospective study included 28 patients (56 eyes) who underwent SMILE at the Peking Union Medical College Hospital (Beijing, China) between January 2021 and June 2021. The inclusion criteria were as follows: age ≥ 18 years; refractive errors were stable for at least 2 years; spherical refraction less than −10 diopters (D); astigmatism less than −5 diopters (D); and the absence of abnormal corneal topography and other ocular diseases. SMILE was performed by the same surgeon (Q.L) using the VisuMax system (500 kHz) (Carl Zeiss Meditec AG, Jena, Germany). The study was approved by the Ethics Committee of Peking Union Medical College Hospital (Beijing, China) and complied with the tenets of the Declaration of Helsinki. Informed content was obtained from all patients.

### Preoperative measurements and clinical outcomes

The preoperative measurements including uncorrected visual acuity, corrected visual acuity, corneal thickness, corneal topography, manifest and cycloplegic refractions. Ocular aberrations were measured with a ray-tracing aberrometer, iTrace system (Tracey Technologies, Houston, TX, United States) at baseline, 1 week, 1 month and 3 months after surgery. The total corneal high-order aberrations (tHOAs), coma, spherical aberration (SA), trefoil aberration, point spread function (PSF), and modulation transfer function (MTF) cut-off values were recorded.

### OBL measurement

The OBL area was measured as previously reported (18). Briefly, the video recordings of the SMILE surgeries were extracted from the VisuMax storage system. After the side cut was completed, the video was paused immediately, and the image was captured in a JPEG format using a “screenshot.” After inputting the JPEG files to Adobe Photoshop CS6 software (Adobe Systems, San Jose, CA), the total corneal area was selected using the elliptical marque tool. The mean luminosity and standard deviation were recorded. The percentage of pixels that surpassed the threshold (mean luminosity + two standard deviations) was recorded as the OBL area.

### Statistical analysis

Continuous variables are expressed as mean ± standard deviation. Generalized Estimating Equation was used to compare the preoperative and postoperative visual quality parameters between groups. Linear mixed effects model was performed to evaluate the correlation between the OBL area and the various parameters. The statistical analyses were performed using SPSS software (version 17.0; SPSS, Inc., Chicago, IL). A *p* value less than 0.05 was considered statistically significant.

## Results

Twenty-eight patients (56 eyes) were included in this study. The mean OBL area was 2.75 ± 1.25 (%). To further analyze the potential risk factors of OBL formation, we divided the patients into two groups based on whether the OBL area was greater than the mean or if the OBL area was less than the mean. The OBL greater than the mean group had less spherical value (*p* = 0.02), steeper corneal curvature (*p* < 0.01), thinner cap thickness (*p* = 0.03) compared to the OBL less than the mean group. The central corneal thickness (CCT) and residual stroma thickness (RST) was larger in the OBL greater than the mean group, but the difference was not statistically significant. The other parameters (age, astigmatism, and lenticule thickness) were not significantly different (Table 1).

**Table 1:** Comparison of baseline characteristics and surgical designs between two groups.

Parameters	Total	OBL ≥ mean group	OBL < mean group	*p* value
OBL area (%)	2.75 ± 1.25	3.76 ± 0.61	1.68 ± 0.77	<0.01
Age (years)	30.1 ± 5.7	29.9 ± 4.9	30.3 ± 6.6	0.86
Sphere (D)	−5.31 ± 1.62	−4.79 ± 1.35	−5.87 ± 1.71	0.02*
Astigmatism (D)	−0.62 ± 0.55	−0.49 ± 0.39	−0.75 ± 0.66	0.15
Keratometry (D)	43.54 ± 1.25	44.14 ± 1.12	42.97 ± 1.13	<0.01*
CCT (μm)	524.9 ± 25.5	528.45 ± 19.14	521.19 ± 26.62	0.38
Cap thickness (μm)	118.83 ± 2.85	119.65 ± 1.85	117.96 ± 3.46	0.03*
Lenticule thickness (μm)	103.5 ± 18.5	99.97 ± 16.45	107.33 ± 20.12	0.21
RST (μm)	302.6 ± 25.5	308.83 ± 26.46	295.89 ± 22.95	0.12

OBL, opaque bubble layer; CCT, central corneal thickness; RST, residual stroma thickness. *p ≤ 0.05.

Linear mixed effects model was applied to explore the potential associations between the OBL area and various parameters. OBL area was positively correlated with keratomerty (*r* = 0.21, *p* = 0.04), and preoperative sphere (*r* = 0.47, *p* = 0.01) (Table 2).

**Table 2:** Correlation analysis of OBL area with preoperative parameters.

	Sphere	Keratometry	CCT	Lenticule thickness	RST
*r*	0.47	0.21	0.68	−3.20	4.37
*p*	0.01*	0.04*	0.41	0.15	0.09

OBL, opaque bubble layer; CCT, central corneal thickness; RST, residual stroma thickness. *p ≤ 0.05.

**Figure 1:**
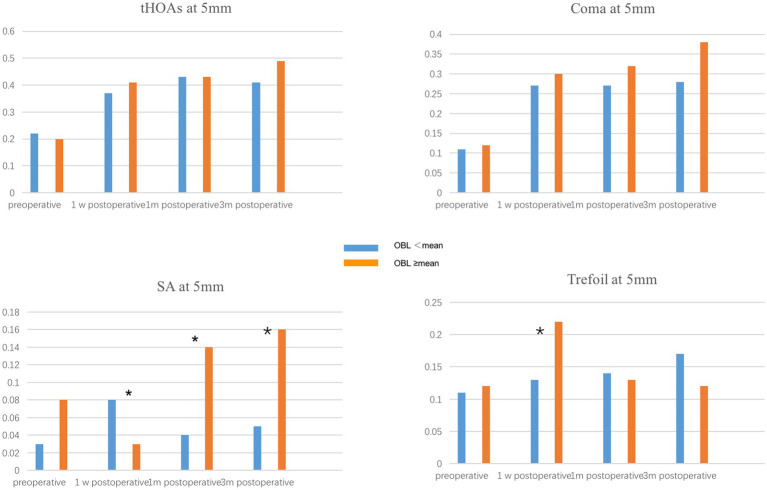
Comparison of corneal HOAs at 5 mm between the two groups. SA (spherical aberration) and trefoil were higher in the OBL greater than the mean group 1 week postoperatively. *p ≤ 0.05.

**Figure 2:**
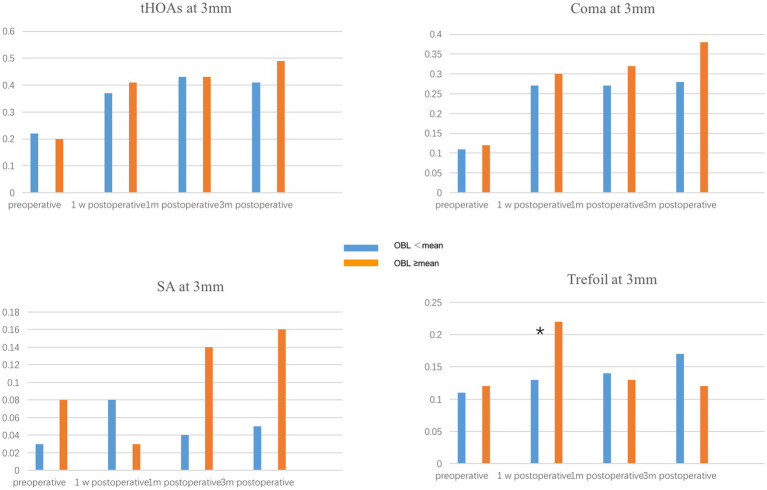
Comparison of corneal HOAs at 3 mm between the two groups. Trefoil was higher in the OBL greater than the mean group 1 week postoperatively. *p ≤ 0.05.

To evaluate the effect of OBL on optical quality, we compared the corneal high-order aberration parameters, including tHOAs, coma, SA, and trefoil at 3 mm and 5 mm pupil size. The preoperative corneal high-order aberrations were not significantly different between the two groups. In the 5 mm pupil analysis, SA and trefoil were higher in the OBL greater than the mean group 1 week postoperatively, but there was no significant difference in trefoil between the groups 1-month and 3-months postoperatively (Figure 1). However, SA was still higher in the OBL greater than the mean group at the end of the follow-up. In the 3 mm pupil size analysis, SA and trefoil were also higher in the OBL greater than the mean group 1 week postoperatively, but the difference was not significant at 1-month and 3-months postoperatively. There was no significant difference in tHOAs and coma between the two groups (Figure 2).

The PSF and MTF values were measured pre-operation, 1-week, 1-month, and 3-months post-operation by iTrace. In the 5 mm pupil analysis, the PSF and MTF values were not significantly different between the two groups pre-operation and post-operation. In the 3 mm pupil analysis, there was no statistical difference in the PSF and MTF values preoperatively. However, the MTF values were higher in the OBL less than the mean group at 1-week postoperative, and the difference between the two groups was not significant at 1- month and 3-months postoperatively (Table 3).

**Table 3:** Comparison of ocular optical quality between the two groups.

	Preoperative			1 w postoperative			1 m postoperative			3 m postoperative		
	OBL ≥ mean	OBL < mean	*p*	OBL ≥ mean	OBL < mean	*p*	OBL ≥ mean	OBL < mean	*p*	OBL ≥ mean	OBL < mean	*p*
PSF at 5 mm	0.12 ± 0.2	0.09 ± 0.12	0.66	0.09 ± 0.08	0.10 ± 0.10	0.71	0.12 ± 0.10	0.16 ± 0.15	0.18	0.27 ± 0.28	0.13 ± 0.28	0.06
MTF at 5 mm	0.30 ± 0.12	0.30 ± 0.09	0.97	0.38 ± 0.11	0.32 ± 0.11	0.38	0.33 ± 0.16	0.31 ± 0.09	0.62	0.52 ± 0.12	0.46 ± 0.09	0.09
PSF at 3 mm	0.16 ± 0.15	0.11 ± 0.07	0.31	0.23 ± 0.14	0.20 ± 0.13	0.55	0.24 ± 0.19	0.21 ± 0.14	0.61	0.32 ± 0.21	0.19 ± 0.2	0.06
MTF at 3 mm	0.35 ± 0.12	0.33 ± 0.09	0.60	0.21 ± 0.14	0.44 ± 0.12	<0.01*	0.27 ± 0.12	0.36 ± 0.15	0.08	0.47 ± 0.10	0.44 ± 0.07	0.54

OBL, opaque bubble layer; PSF, point spread function; MTF, modulation transfer function. *p ≤ 0.05.

## Discussion

In this study, we evaluated the relationship between OBL area and preoperative parameters and found that a steep corneal curvature, thin cap thickness, and higher spherical value are possible risk factors for OBL formation in SMILE surgery. This study is valuable as it further evaluated the effect of OBL on HOAs and visual quality parameters and found that the OBL increased corneal HOAs postoperatively for a short period, which has not been reported before.

In the present study, the mean OBL area was 2.75% ± 1.25% of the total cornea, which was similar to a previous study (18). However, the incidence of OBL varies among different studies. For instance, the incidence of OBL in FS-LASIK varies from 5 to 72.51% (11, 12, 19). Furthermore, Ma et al. found the incidence of OBL in SMILE was 0.73% (13), which was quite lower than Son’s finding at 51.82% (18). These different incidences may be attributed to different surgical parameters, laser designs, and docking methods.

A thicker cornea, small cap, steeper corneal curvature, flap shape, and hard docking technique are possible risk factors of OBL formation in FS-LASIK surgery as previously reported (5, 11, 20). Our results revealed that OBL tended to be more prominent when the sphere was larger, the cornea was steeper, and the cap thickness was thin. The OBL area was positively correlated with those parameters, but not the CCT. Previous studies found that a thicker cornea was a risk factor for OBL formation in both FS-LASIK and SMILE surgeries, but the correlation was relatively weak when the CCT was below 550 um (13). A majority of the cases in our study had a CCT of less than 550 um, which could explain why the OBL area did not correlate with CCT.

RST is another risk factor for OBL development in SMILE surgery. Ma et al. found that when RST increased 1 um, the risk of OBL formation increased 3% (13). Our study also found that OBL tended to develop when there was a thicker RST and thinner cap thickness. The anterior cornea stroma is denser, which forms more gas bubbles during photoablation, and gas bubbles might not be able to dissipate, resulting in OBL. In addition, corneal biomechanics may also play a role in the OBL formation. A previous study reported that higher corneal resistance factor (CRF) and cornea hysteresis (CH) values resulted in more severe OBL (5, 21). The anterior part of the cornea has a stronger biomechanical strength, which could explain why OBL tended to develop if the cap thickness was thin.

Our results also showed that a steeper cornea tended to increase the formation of OBL. A steeper cornea could narrow the gap between the contact glass and generate greater pressure, which may prevent the gas bubbles from dissipating. In addition, Li et al. found that the risk of OBL decreased with increasing myopia (22). We also found that OBL negatively correlated with the spherical value. The spherical value is an important factor for determining the scanning depth, which indicates that deepening the photodisruption plane could reduce OBL formation when corrected for low myopia. Other risk factors, such as the laser energy and pulse rate, were slightly changed in the present study. Further studies are needed to explore the relationship between those factors and OBL formation in SMILE surgery.

Several studies have demonstrated that OBL has no significant effect on postoperative visual outcomes (15, 23). Our results showed that OBL increased corneal HOAs, especially SA and trefoil, in both 5 mm and 3 mm pupil analyses in the short term after surgery. One possible explanation is that the subjective image focus appears to be driven primarily by the overall amount of blur and only weakly by HOA blur orientation. Furthermore, vision calibrates itself to specific blur levels present in each individual’s retinal image. Therefore, although the formation of OBL increased the higher-order aberrations, it does not have an impact on visual acuity (14, 15). Furthermore, a majority of the OBL was located at the periphery of the cornea, which could explain why the effect of OBL on corneal HOAs was more significant in the 5 mm pupil analysis.

MTF refers to the ratio between the image contrast of a specific object and the contrast of the object itself at different spatial frequencies, generally, the higher MTF represents better ocular optical quality. SPF reveals how a single spot is visualized considering optical aberration using iTrace point square function. They both are important objective methods to evaluate optical quality. This study found that the MTF values were higher in the OBL less than the mean group at 1-week postoperative, but the difference between the two groups was not significant at 1- month and 3-months postoperatively. This result further supported the fact that OBL decreased visual quality postoperatively for a short period, while it did not affect the long-term outcomes.

We did not investigate the effect of OBL on visual acuity and refraction, as several studies have proved that OBL has no effect on visual acuity and refraction. However, there are several limitations of this study. Firstly, the sample size is small and follow-up time was relatively limited. Secondly, Other risk factors, such as the laser energy and pulse rate, were not evaluated in the present study. Further studies are needed to explore the relationship between those factors and OBL formation in SMILE surgery.

In summary, a steep corneal curvature, and thin cap thickness are possible risk factors for OBL formation in SMILE surgery. Although OBL did not affect visual outcomes, it may induce more HOAs and decrease visual quality in the short term after surgery.

## Data availability statement

The raw data supporting the conclusions of this article will be made available by the authors, without undue reservation.

## Ethics statement

The studies involving humans were approved by Ethics Committee of Peking Union Medical College Hospital. The studies were conducted in accordance with the local legislation and institutional requirements. The participants provided their written informed consent to participate in this study.

## Author contributions

SY, ZC, and QL contributed to conception and design of the study. SY and HW organized the database. SY performed the statistical analysis and wrote the first draft of the manuscript. ZC, HW, YL, and QL wrote sections of the manuscript. YC contributed to analysis of data and revised the manuscript. All authors contributed to the article and approved the submitted version.
