# Corrigendum: Possible risk factors of opaque bubble layer and its effect on high-order aberrations after small incision Lenticule extraction

**DOI:** 10.3389/fmed.2023.1360144

**Published:** 2024-01-11

**Authors:** Shan Yang, Heng Wang, Zhengyu Chen, Ying Li, Youxin Chen, Qin Long

**Affiliations:** Department of Ophthalmology, Peking Union Medical College Hospital, Chinese Academy of Medical Sciences and Peking Union Medical College, Beijing, China

**Keywords:** opaque bubble layer, high-order aberration, small incision lenticule extraction, short term, visual quality

In the published article, there was an error in the author list order. The corrected author list, citation and copyright appears below.

Shan Yang, Heng Wang, Zhengyu Chen, Ying Li, Youxin Chen, Qin Long

The authors apologize for this error and state that this does not change the scientific conclusions of the article in any way. The original article has been updated.

