# Supplementary material for: Possible risk factors of opaque bubble layer and its effect on high-order aberrations after small incision Lenticule extraction
Source: Front Med (Lausanne). 2023 Dec 20;10:1156677. doi: 10.3389/fmed.2023.1156677 (PMC10765512; doi:10.3389/fmed.2023.1156677)
Supplement: Supplementary file 1 [file Data_Sheet_1.PDF]

```
 * Generalized Estimating Equations.
GENLIN sphere BY group eyes (ORDER=ASCENDING)
  /MODEL group eyes group*eyes INTERCEPT=YES
 DISTRIBUTION=NORMAL LINK=IDENTITY
  /CRITERIA SCALE=MLE PCONVERGE=1E-006(ABSOLUTE) SINGULAR=1E-012 ANALYSISTY
PE=3(WALD) CILEVEL=95
    LIKELIHOOD=FULL
  /REPEATED SUBJECT=patients WITHINSUBJECT=eyes SORT=YES CORRTYPE=INDEPENDE
NT ADJUSTCORR=YES
    COVB=ROBUST
  /MISSING CLASSMISSING=EXCLUDE
  /PRINT CPS DESCRIPTIVES MODELINFO FIT SUMMARY SOLUTION.
```

Generalized Linear Models

Notes

Output Created		28-NOV-2023 15:45...
Comments		
Input	Data	/Users/yangshan/Desktop/2022-5-12//2023-10-OBL/2023-11-22 revised manuscript/OBL.sav
	Active Dataset	DataSet1
	Filter	<none>
	Weight	<none>
	Split File	<none>
	N of Rows in Working Data File	56
Missing Value Handling	Definition of Missing	User-defined missing values for factor, subject and within-subject variables are treated as missing.
	Cases Used	Statistics are based on cases with valid data for all variables in the model.
Weight Handling		not applicable

Notes

Syntax		GENLIN sphere BY group eyes (ORDER=ASCENDING)<br>/MODEL group eyes group*eyes INTERCEPT=YES<br><br>DISTRIBUTION=NORMAL LINK=IDENTITY<br>/CRITERIA SCALE=MLE PCONVERGE=1E-006 (ABSOLUTE) SINGULAR=1E-012 ANALYSISTYPE=3(WALD) CILEVEL=95<br>LIKELIHOOD=FULL<br>/REPEATED SUBJECT=patients WITHINSUBJECT=eyes SORT=YES CORRTYPE=INDEPENDENT ADJUSTCORR=YES<br>COVB=ROBUST<br>/MISSING CLASSMISSING=EXCLUDE<br>/PRINT CPS DESCRIPTIVES MODELINFO FIT SUMMARY SOLUTION.
Resources	Processor Time	00:00:00.04
	Elapsed Time	00:00:00.00

Model Information

Dependent Variable		sphere
Probability Distribution		Normal
Link Function		Identity
Subject Effect	1	patients
Within-Subject Effect	1	eyes
Working Correlation Matrix Structure		Independent

Case Processing Summary

	N	Percent
Included	56	100.0%
Excluded	0	0.0%
Total	56	100.0%

Correlated Data Summary

Number of Levels	Subject Effect	patients	32
	Within-Subject Effect	eyes	2
Number of Subjects			32
Number of Measurements per Subject	Minimum		1
	Maximum		2
Correlation Matrix Dimension			2

Categorical Variable Information

			N	Percent
Factor	group	1	29	51.8%
		2	27	48.2%
		Total	56	100.0%
	eyes	1	32	57.1%
		2	24	42.9%
		Total	56	100.0%

Continuous Variable Information

		N	Minimum	Maximum	Mean	Std. Deviation
Dependent Variable	sphere	56	-9.65	-2.75	-5.3098	1.61992

Goodness of Fit[a]

	Value
Quasi Likelihood under Independence Model Criterion (QIC)[b]	133.868
Corrected Quasi Likelihood under Independence Model Criterion (QICC)[b]	133.893

Dependent Variable: sphere
Model: (Intercept), group, eyes, group * eyes[a]

a. Information criteria are in smaller-is-better form.

b. Computed using the full log quasi-likelihood function.

Tests of Model Effects

Source	Type III		
	Wald Chi-Square	df	Sig.
(Intercept)	445.405	1	.000
group	5.084	1	.024
eyes	.108	1	.743
group * eyes	2.056	1	.152

Dependent Variable: sphere
Model: (Intercept), group, eyes, group * eyes

Parameter Estimates

Parameter	B	Std. Error	95% Wald Confidence Interval		Hypothesis ...
			Lower	Upper	Wald Chi-Square
(Intercept)	-6.033	.4946	-7.003	-5.064	148.822
[group=1]	1.521	.5927	.359	2.682	6.585
[group=2]	0[a]	.	.	.	.
[eyes=1]	.297	.4266	-.539	1.133	.484
[eyes=2]	0[a]	.	.	.	.
[group=1] * [eyes=1]	-.769	.5367	-1.821	.282	2.056
[group=1] * [eyes=2]	0[a]	.	.	.	.
[group=2] * [eyes=1]	0[a]	.	.	.	.
[group=2] * [eyes=2]	0[a]	.	.	.	.
(Scale)	2.421				

Parameter Estimates

Parameter	Hypothesis Test	
	df	Sig.
(Intercept)	1	.000
[group=1]	1	.010
[group=2]	.	.
[eyes=1]	1	.487
[eyes=2]	.	.
[group=1] * [eyes=1]	1	.152
[group=1] * [eyes=2]	.	.
[group=2] * [eyes=1]	.	.
[group=2] * [eyes=2]	.	.
(Scale)		

Dependent Variable: sphere
Model: (Intercept), group, eyes, group * eyes

a. Set to zero because this parameter is redundant.

```
 * Generalized Estimating Equations.
GENLIN astigmatism BY group eyes (ORDER=ASCENDING)
  /MODEL group eyes group*eyes INTERCEPT=YES
 DISTRIBUTION=NORMAL LINK=IDENTITY
  /CRITERIA SCALE=MLE PCONVERGE=1E-006(ABSOLUTE) SINGULAR=1E-012 ANALYSISTYPE=3(WALD) CILEVEL=95
    LIKELIHOOD=FULL
  /REPEATED SUBJECT=patients WITHINSUBJECT=eyes SORT=YES CORRTYPE=INDEPENDENT ADJUSTCORR=YES
    COVB=ROBUST
  /MISSING CLASSMISSING=EXCLUDE
  /PRINT CPS DESCRIPTIVES MODELINFO FIT SUMMARY SOLUTION.
```

Generalized Linear Models

Notes

Output Created		28-NOV-2023 15:45...
Comments		
Input	Data	/Users/yangshan/Desktop/2022-5-12//2023-10-OBL/2023-11-22 revised manuscript/OBL.sav
	Active Dataset	DataSet1
	Filter	<none>
	Weight	<none>
	Split File	<none>
	N of Rows in Working Data File	56
Missing Value Handling	Definition of Missing	User-defined missing values for factor, subject and within-subject variables are treated as missing.
	Cases Used	Statistics are based on cases with valid data for all variables in the model.
Weight Handling		not applicable

Notes

Syntax		GENLIN astigmatism BY group eyes (ORDER=ASCENDING)<br>/MODEL group eyes group*eyes INTERCEPT=YES<br><br>DISTRIBUTION=NORMAL LINK=IDENTITY<br>/CRITERIA SCALE=MLE PCONVERGE=1E-006 (ABSOLUTE) SINGULAR=1E-012 ANALYSISTYPE=3(WALD) CILEVEL=95<br>LIKELIHOOD=FULL<br>/REPEATED SUBJECT=patients WITHINSUBJECT=eyes SORT=YES CORRTYPE=INDEPENDENT ADJUSTCORR=YES<br>COVB=ROBUST<br>/MISSING CLASSMISSING=EXCLUDE<br>/PRINT CPS DESCRIPTIVES MODELINFO FIT SUMMARY SOLUTION.
Resources	Processor Time	00:00:00.04
	Elapsed Time	00:00:01.00

Model Information

Dependent Variable		astigmatism
Probability Distribution		Normal
Link Function		Identity
Subject Effect	1	patients
Within-Subject Effect	1	eyes
Working Correlation Matrix Structure		Independent

Case Processing Summary

	N	Percent
Included	56	100.0%
Excluded	0	0.0%
Total	56	100.0%

Correlated Data Summary

Number of Levels	Subject Effect	patients	32
	Within-Subject Effect	eyes	2
Number of Subjects			32
Number of Measurements per Subject	Minimum		1
	Maximum		2
Correlation Matrix Dimension			2

Categorical Variable Information

			N	Percent
Factor	group	1	29	51.8%
		2	27	48.2%
		Total	56	100.0%
	eyes	1	32	57.1%
		2	24	42.9%
		Total	56	100.0%

Continuous Variable Information

		N	Minimum	Maximum	Mean	Std. Deviation
Dependent Variable	astigmatism	56	-2.50	.00	-.6161	.54972

Goodness of Fit[a]

	Value
Quasi Likelihood under Independence Model Criterion (QIC)[b]	24.041
Corrected Quasi Likelihood under Independence Model Criterion (QICC)[b]	23.646

Dependent Variable: astigmatism
Model: (Intercept), group, eyes, group * eyes[a]

a. Information criteria are in smaller-is-better form.

b. Computed using the full log quasi-likelihood function.

Tests of Model Effects

Source	Type III		
	Wald Chi-Square	df	Sig.
(Intercept)	48.283	1	.000
group	2.018	1	.155
eyes	.070	1	.791
group * eyes	.155	1	.694

Dependent Variable: astigmatism
Model: (Intercept), group, eyes, group * eyes

Parameter Estimates

Parameter	B	Std. Error	95% Wald Confidence Interval		Hypothesis ...
			Lower	Upper	Wald Chi-Square
(Intercept)	-.708	.2241	-1.147	-.269	9.994
[group=1]	.208	.2497	-.281	.698	.696
[group=2]	0[a]	.	.	.	.
[eyes=1]	-.075	.2076	-.482	.332	.130
[eyes=2]	0[a]	.	.	.	.
[group=1] * [eyes=1]	.090	.2277	-.357	.536	.155
[group=1] * [eyes=2]	0[a]	.	.	.	.
[group=2] * [eyes=1]	0[a]	.	.	.	.
[group=2] * [eyes=2]	0[a]	.	.	.	.
(Scale)	.301				

Parameter Estimates

Parameter	Hypothesis Test	
	df	Sig.
(Intercept)	1	.002
[group=1]	1	.404
[group=2]	.	.
[eyes=1]	1	.718
[eyes=2]	.	.
[group=1] * [eyes=1]	1	.694
[group=1] * [eyes=2]	.	.
[group=2] * [eyes=1]	.	.
[group=2] * [eyes=2]	.	.
(Scale)		

Dependent Variable: astigmatism
Model: (Intercept), group, eyes, group * eyes

a. Set to zero because this parameter is redundant.

```
 * Generalized Estimating Equations.
GENLIN Km BY group eyes (ORDER=ASCENDING)
  /MODEL group eyes group*eyes INTERCEPT=YES
 DISTRIBUTION=NORMAL LINK=IDENTITY
  /CRITERIA SCALE=MLE PCONVERGE=1E-006(ABSOLUTE) SINGULAR=1E-012 ANALYSISTY
PE=3(WALD) CILEVEL=95
    LIKELIHOOD=FULL
  /REPEATED SUBJECT=patients WITHINSUBJECT=eyes SORT=YES CORRTYPE=INDEPENDE
NT ADJUSTCORR=YES
    COVB=ROBUST
  /MISSING CLASSMISSING=EXCLUDE
  /PRINT CPS DESCRIPTIVES MODELINFO FIT SUMMARY SOLUTION.
```

Generalized Linear Models

Notes

Output Created		28-NOV-2023 15:46...
Comments		
Input	Data	/Users/yangshan/Desktop/2022-5-12//2023-10-OBL/2023-11-22 revised manuscript/OBL.sav
	Active Dataset	DataSet1
	Filter	<none>
	Weight	<none>
	Split File	<none>
	N of Rows in Working Data File	56
Missing Value Handling	Definition of Missing	User-defined missing values for factor, subject and within-subject variables are treated as missing.
	Cases Used	Statistics are based on cases with valid data for all variables in the model.
Weight Handling		not applicable

Notes

Syntax		GENLIN Km BY group eyes (ORDER=ASCENDING)<br>/MODEL group eyes group*eyes INTERCEPT=YES<br><br>DISTRIBUTION=NORMAL LINK=IDENTITY<br>/CRITERIA SCALE=MLE PCONVERGE=1E-006 (ABSOLUTE) SINGULAR=1E-012 ANALYSISTYPE=3(WALD) CILEVEL=95<br>LIKELIHOOD=FULL<br>/REPEATED SUBJECT=patients WITHINSUBJECT=eyes SORT=YES CORRTYPE=INDEPENDENT ADJUSTCORR=YES<br>COVB=ROBUST<br>/MISSING CLASSMISSING=EXCLUDE<br>/PRINT CPS DESCRIPTIVES MODELINFO FIT SUMMARY SOLUTION.
Resources	Processor Time	00:00:00.04
	Elapsed Time	00:00:00.00

Model Information

Dependent Variable		Km
Probability Distribution		Normal
Link Function		Identity
Subject Effect	1	patients
Within-Subject Effect	1	eyes
Working Correlation Matrix Structure		Independent

Case Processing Summary

	N	Percent
Included	56	100.0%
Excluded	0	0.0%
Total	56	100.0%

Correlated Data Summary

Number of Levels	Subject Effect	patients	32
	Within-Subject Effect	eyes	2
Number of Subjects			32
Number of Measurements per Subject	Minimum		1
	Maximum		2
Correlation Matrix Dimension			2

Categorical Variable Information

			N	Percent
Factor	group	1	29	51.8%
		2	27	48.2%
		Total	56	100.0%
	eyes	1	32	57.1%
		2	24	42.9%
		Total	56	100.0%

Continuous Variable Information

		N	Minimum	Maximum	Mean	Std. Deviation
Dependent Variable	Km	56	40.93	46.02	43.5373	1.25631

Goodness of Fit[a]

	Value
Quasi Likelihood under Independence Model Criterion (QIC)[b]	75.297
Corrected Quasi Likelihood under Independence Model Criterion (QICC)[b]	75.260

Dependent Variable: Km
Model: (Intercept), group, eyes, group * eyes[a]

a. Information criteria are in smaller-is-better form.

b. Computed using the full log quasi-likelihood function.

Tests of Model Effects

Source	Type III		
	Wald Chi-Square	df	Sig.
(Intercept)	45023.631	1	.000
group	8.413	1	.004
eyes	.508	1	.476
group * eyes	3.616	1	.057

Dependent Variable: Km
Model: (Intercept), group, eyes, group * eyes

Parameter Estimates

Parameter	B	Std. Error	95% Wald Confidence Interval		Hypothesis ...
			Lower	Upper	Wald Chi-Square
(Intercept)	44.276	.3256	43.638	44.914	18488.422
[group=1]	-1.366	.4558	-2.259	-.472	8.978
[group=2]	0[a]	.	.	.	.
[eyes=1]	-.241	.1300	-.495	.014	3.421
[eyes=2]	0[a]	.	.	.	.
[group=1] * [eyes=1]	.350	.1840	-.011	.711	3.616
[group=1] * [eyes=2]	0[a]	.	.	.	.
[group=2] * [eyes=1]	0[a]	.	.	.	.
[group=2] * [eyes=2]	0[a]	.	.	.	.
(Scale)	1.293				

Parameter Estimates

Parameter	Hypothesis Test	
	df	Sig.
(Intercept)	1	.000
[group=1]	1	.003
[group=2]	.	.
[eyes=1]	1	.064
[eyes=2]	.	.
[group=1] * [eyes=1]	1	.057
[group=1] * [eyes=2]	.	.
[group=2] * [eyes=1]	.	.
[group=2] * [eyes=2]	.	.
(Scale)		

Dependent Variable: Km
Model: (Intercept), group, eyes, group * eyes

a. Set to zero because this parameter is redundant.

```
 * Generalized Estimating Equations.
GENLIN CCT BY group eyes (ORDER=ASCENDING)
  /MODEL group eyes group*eyes INTERCEPT=YES
 DISTRIBUTION=NORMAL LINK=IDENTITY
  /CRITERIA SCALE=MLE PCONVERGE=1E-006(ABSOLUTE) SINGULAR=1E-012 ANALYSISTY
PE=3(WALD) CILEVEL=95
    LIKELIHOOD=FULL
  /REPEATED SUBJECT=patients WITHINSUBJECT=eyes SORT=YES CORRTYPE=INDEPENDE
NT ADJUSTCORR=YES
    COVB=ROBUST
  /MISSING CLASSMISSING=EXCLUDE
  /PRINT CPS DESCRIPTIVES MODELINFO FIT SUMMARY SOLUTION.
```

Generalized Linear Models

Notes

Output Created		28-NOV-2023 15:46...
Comments		
Input	Data	/Users/yangshan/Desktop/2022-5-12/ /2023-10-OBL/2023-11-22 revised manuscript/OBL.sav
	Active Dataset	DataSet1
	Filter	<none>
	Weight	<none>
	Split File	<none>
	N of Rows in Working Data File	56
Missing Value Handling	Definition of Missing	User-defined missing values for factor, subject and within-subject variables are treated as missing.
	Cases Used	Statistics are based on cases with valid data for all variables in the model.
Weight Handling		not applicable

Notes

Syntax		GENLIN CCT BY group eyes (ORDER=ASCENDING)<br>/MODEL group eyes group*eyes INTERCEPT=YES<br><br>DISTRIBUTION=NORMAL LINK=IDENTITY<br>/CRITERIA SCALE=MLE PCONVERGE=1E-006 (ABSOLUTE) SINGULAR=1E-012 ANALYSISTYPE=3(WALD) CILEVEL=95<br>LIKELIHOOD=FULL<br>/REPEATED SUBJECT=patients WITHINSUBJECT=eyes SORT=YES CORRTYPE=INDEPENDENT ADJUSTCORR=YES<br>COVB=ROBUST<br>/MISSING CLASSMISSING=EXCLUDE<br>/PRINT CPS DESCRIPTIVES MODELINFO FIT SUMMARY SOLUTION.
Resources	Processor Time	00:00:00.04
	Elapsed Time	00:00:00.00

Model Information

Dependent Variable		CCT
Probability Distribution		Normal
Link Function		Identity
Subject Effect	1	patients
Within-Subject Effect	1	eyes
Working Correlation Matrix Structure		Independent

Case Processing Summary

	N	Percent
Included	56	100.0%
Excluded	0	0.0%
Total	56	100.0%

Correlated Data Summary

Number of Levels	Subject Effect	patients	32
	Within-Subject Effect	eyes	2
Number of Subjects			32
Number of Measurements per Subject	Minimum		1
	Maximum		2
Correlation Matrix Dimension			2

Categorical Variable Information

			N	Percent
Factor	group	1	29	51.8%
		2	27	48.2%
		Total	56	100.0%
	eyes	1	32	57.1%
		2	24	42.9%
		Total	56	100.0%

Continuous Variable Information

		N	Minimum	Maximum	Mean	Std. Deviation
Dependent Variable	CCT	56	484.00	581.00	524.9464	23.13551

Goodness of Fit[a]

	Value
Quasi Likelihood under Independence Model Criterion (QIC)[b]	28568.237
Corrected Quasi Likelihood under Independence Model Criterion (QICC)[b]	28568.037

Dependent Variable: CCT
Model: (Intercept), group, eyes, group * eyes[a]

a. Information criteria are in smaller-is-better form.

b. Computed using the full log quasi-likelihood function.

Tests of Model Effects

Source	Type III		
	Wald Chi-Square	df	Sig.
(Intercept)	15104.751	1	.000
group	.766	1	.381
eyes	1.882	1	.170
group * eyes	.146	1	.703

Dependent Variable: CCT
Model: (Intercept), group, eyes, group * eyes

Parameter Estimates

Parameter	B	Std. Error	95% Wald Confidence Interval		Hypothesis ...
			Lower	Upper	Wald Chi-Square
(Intercept)	522.417	7.9778	506.780	538.053	4288.131
[group=1]	8.333	9.7927	-10.860	27.527	.724
[group=2]	0[a]	.	.	.	.
[eyes=1]	-2.217	3.3220	-8.728	4.294	.445
[eyes=2]	0[a]	.	.	.	.
[group=1] * [eyes=1]	-1.710	4.4775	-10.485	7.066	.146
[group=1] * [eyes=2]	0[a]	.	.	.	.
[group=2] * [eyes=1]	0[a]	.	.	.	.
[group=2] * [eyes=2]	0[a]	.	.	.	.
(Scale)	549.231				

Parameter Estimates

Parameter	Hypothesis Test	
	df	Sig.
(Intercept)	1	.000
[group=1]	1	.395
[group=2]	.	.
[eyes=1]	1	.505
[eyes=2]	.	.
[group=1] * [eyes=1]	1	.703
[group=1] * [eyes=2]	.	.
[group=2] * [eyes=1]	.	.
[group=2] * [eyes=2]	.	.
(Scale)		

Dependent Variable: CCT
Model: (Intercept), group, eyes, group * eyes

a. Set to zero because this parameter is redundant.

```
 * Generalized Estimating Equations.
GENLIN cap BY group eyes (ORDER=ASCENDING)
  /MODEL group eyes group*eyes INTERCEPT=YES
 DISTRIBUTION=NORMAL LINK=IDENTITY
  /CRITERIA SCALE=MLE PCONVERGE=1E-006(ABSOLUTE) SINGULAR=1E-012 ANALYSISTY
PE=3(WALD) CILEVEL=95
    LIKELIHOOD=FULL
  /REPEATED SUBJECT=patients WITHINSUBJECT=eyes SORT=YES CORRTYPE=INDEPENDE
NT ADJUSTCORR=YES
    COVB=ROBUST
  /MISSING CLASSMISSING=EXCLUDE
  /PRINT CPS DESCRIPTIVES MODELINFO FIT SUMMARY SOLUTION.
```

Generalized Linear Models

Notes

Output Created		28-NOV-2023 15:47...
Comments		
Input	Data	/Users/yangshan/Desktop/2022-5-12//2023-10-OBL/2023-11-22 revised manuscript/OBL.sav
	Active Dataset	DataSet1
	Filter	<none>
	Weight	<none>
	Split File	<none>
	N of Rows in Working Data File	56
Missing Value Handling	Definition of Missing	User-defined missing values for factor, subject and within-subject variables are treated as missing.
	Cases Used	Statistics are based on cases with valid data for all variables in the model.
Weight Handling		not applicable

Notes

Syntax		GENLIN cap BY group eyes (ORDER=ASCENDING)<br>/MODEL group eyes group*eyes INTERCEPT=YES<br><br>DISTRIBUTION=NORMAL LINK=IDENTITY<br>/CRITERIA SCALE=MLE PCONVERGE=1E-006 (ABSOLUTE) SINGULAR=1E-012 ANALYSISTYPE=3(WALD) CILEVEL=95<br>LIKELIHOOD=FULL<br>/REPEATED SUBJECT=patients WITHINSUBJECT=eyes SORT=YES CORRTYPE=INDEPENDENT ADJUSTCORR=YES<br>COVB=ROBUST<br>/MISSING CLASSMISSING=EXCLUDE<br>/PRINT CPS DESCRIPTIVES MODELINFO FIT SUMMARY SOLUTION.
Resources	Processor Time	00:00:00.04
	Elapsed Time	00:00:00.00

Model Information

Dependent Variable		cap
Probability Distribution		Normal
Link Function		Identity
Subject Effect	1	patients
Within-Subject Effect	1	eyes
Working Correlation Matrix Structure		Independent

Case Processing Summary

	N	Percent
Included	56	100.0%
Excluded	0	0.0%
Total	56	100.0%

Correlated Data Summary

Number of Levels	Subject Effect	patients	32
	Within-Subject Effect	eyes	2
Number of Subjects			32
Number of Measurements per Subject	Minimum		1
	Maximum		2
Correlation Matrix Dimension			2

Categorical Variable Information

			N	Percent
Factor	group	1	29	51.8%
		2	27	48.2%
		Total	56	100.0%
	eyes	1	32	57.1%
		2	24	42.9%
		Total	56	100.0%

Continuous Variable Information

		N	Minimum	Maximum	Mean	Std. Deviation
Dependent Variable	cap	56	110.00	120.00	118.8393	2.85897

Goodness of Fit[a]

	Value
Quasi Likelihood under Independence Model Criterion (QIC)[b]	410.524
Corrected Quasi Likelihood under Independence Model Criterion (QICC)[b]	410.451

Dependent Variable: cap
Model: (Intercept), group, eyes, group * eyes[a]

a. Information criteria are in smaller-is-better form.

b. Computed using the full log quasi-likelihood function.

Tests of Model Effects

Source	Type III		
	Wald Chi-Square	df	Sig.
(Intercept)	79865.413	1	.000
group	4.528	1	.033
eyes	.042	1	.838
group * eyes	1.404	1	.236

Dependent Variable: cap
Model: (Intercept), group, eyes, group * eyes

Parameter Estimates

Parameter	B	Std. Error	95% Wald Confidence Interval		Hypothesis ...
			Lower	Upper	Wald Chi-Square
(Intercept)	117.500	1.1024	115.339	119.661	11360.571
[group=1]	2.500	1.1024	.339	4.661	5.143
[group=2]	0[a]	.	.	.	.
[eyes=1]	.833	1.0552	-1.235	2.901	.624
[eyes=2]	0[a]	.	.	.	.
[group=1] * [eyes=1]	-1.422	1.1996	-3.773	.930	1.404
[group=1] * [eyes=2]	0[a]	.	.	.	.
[group=2] * [eyes=1]	0[a]	.	.	.	.
[group=2] * [eyes=2]	0[a]	.	.	.	.
(Scale)	7.739				

Parameter Estimates

Parameter	Hypothesis Test	
	df	Sig.
(Intercept)	1	.000
[group=1]	1	.023
[group=2]	.	.
[eyes=1]	1	.430
[eyes=2]	.	.
[group=1] * [eyes=1]	1	.236
[group=1] * [eyes=2]	.	.
[group=2] * [eyes=1]	.	.
[group=2] * [eyes=2]	.	.
(Scale)		

Dependent Variable: cap
Model: (Intercept), group, eyes, group * eyes

a. Set to zero because this parameter is redundant.

```
 * Generalized Estimating Equations.
GENLIN lenticule BY group eyes (ORDER=ASCENDING)
  /MODEL group eyes group*eyes INTERCEPT=YES
 DISTRIBUTION=NORMAL LINK=IDENTITY
  /CRITERIA SCALE=MLE PCONVERGE=1E-006(ABSOLUTE) SINGULAR=1E-012 ANALYSISTYPE=3(WALD) CILEVEL=95
    LIKELIHOOD=FULL
  /REPEATED SUBJECT=patients WITHINSUBJECT=eyes SORT=YES CORRTYPE=INDEPENDENT ADJUSTCORR=YES
    COVB=ROBUST
  /MISSING CLASSMISSING=EXCLUDE
  /PRINT CPS DESCRIPTIVES MODELINFO FIT SUMMARY SOLUTION.
```

Generalized Linear Models

Notes

Output Created		28-NOV-2023 15:47...
Comments		
Input	Data	/Users/yangshan/Desktop/2022-5-12/ /2023-10-OBL/2023-11-22 revised manuscript/OBL .sav
	Active Dataset	DataSet1
	Filter	<none>
	Weight	<none>
	Split File	<none>
	N of Rows in Working Data File	56
Missing Value Handling	Definition of Missing	User-defined missing values for factor, subject and within-subject variables are treated as missing.
	Cases Used	Statistics are based on cases with valid data for all variables in the model.
Weight Handling		not applicable

Notes

Syntax		GENLIN lenticule BY group eyes (ORDER=ASCENDING)<br>/MODEL group eyes group*eyes INTERCEPT=YES<br><br>DISTRIBUTION=NORMAL LINK=IDENTITY<br>/CRITERIA SCALE=MLE PCONVERGE=1E-006 (ABSOLUTE) SINGULAR=1E-012 ANALYSISTYPE=3(WALD) CILEVEL=95<br>LIKELIHOOD=FULL<br>/REPEATED SUBJECT=patients WITHINSUBJECT=eyes SORT=YES CORRTYPE=INDEPENDENT ADJUSTCORR=YES<br>COVB=ROBUST<br>/MISSING CLASSMISSING=EXCLUDE<br>/PRINT CPS DESCRIPTIVES MODELINFO FIT SUMMARY SOLUTION.
Resources	Processor Time	00:00:00.04
	Elapsed Time	00:00:00.00

Model Information

Dependent Variable		lenticule
Probability Distribution		Normal
Link Function		Identity
Subject Effect	1	patients
Within-Subject Effect	1	eyes
Working Correlation Matrix Structure		Independent

Case Processing Summary

	N	Percent
Included	56	100.0%
Excluded	0	0.0%
Total	56	100.0%

Correlated Data Summary

Number of Levels	Subject Effect	patients	32
	Within-Subject Effect	eyes	2
Number of Subjects			32
Number of Measurements per Subject	Minimum		1
	Maximum		2
Correlation Matrix Dimension			2

Categorical Variable Information

			N	Percent
Factor	group	1	29	51.8%
		2	27	48.2%
		Total	56	100.0%
	eyes	1	32	57.1%
		2	24	42.9%
		Total	56	100.0%

Continuous Variable Information

		N	Minimum	Maximum	Mean	Std. Deviation
Dependent Variable	lenticule	56	69.00	146.00	103.5179	18.52369

Goodness of Fit[a]

	Value
Quasi Likelihood under Independence Model Criterion (QIC)[b]	17948.350
Corrected Quasi Likelihood under Independence Model Criterion (QICC)[b]	17948.132

Dependent Variable: lenticule
Model: (Intercept), group, eyes, group * eyes[a]

a. Information criteria are in smaller-is-better form.

b. Computed using the full log quasi-likelihood function.

Tests of Model Effects

Source	Type III		
	Wald Chi-Square	df	Sig.
(Intercept)	1071.115	1	.000
group	1.543	1	.214
eyes	.004	1	.948
group * eyes	1.375	1	.241

Dependent Variable: lenticule
Model: (Intercept), group, eyes, group * eyes

Parameter Estimates

Parameter	B	Std. Error	95% Wald Confidence Interval		Hypothesis ...
			Lower	Upper	Wald Chi-Square
(Intercept)	109.417	6.1061	97.449	121.384	321.102
[group=1]	-11.417	7.9193	-26.938	4.105	2.078
[group=2]	0[a]	.	.	.	.
[eyes=1]	-3.750	4.5299	-12.628	5.128	.685
[eyes=2]	0[a]	.	.	.	.
[group=1] * [eyes=1]	7.103	6.0582	-4.771	18.977	1.375
[group=1] * [eyes=2]	0[a]	.	.	.	.
[group=2] * [eyes=1]	0[a]	.	.	.	.
[group=2] * [eyes=2]	0[a]	.	.	.	.
(Scale)	345.003				

Parameter Estimates

Parameter	Hypothesis Test	
	df	Sig.
(Intercept)	1	.000
[group=1]	1	.149
[group=2]	.	.
[eyes=1]	1	.408
[eyes=2]	.	.
[group=1] * [eyes=1]	1	.241
[group=1] * [eyes=2]	.	.
[group=2] * [eyes=1]	.	.
[group=2] * [eyes=2]	.	.
(Scale)		

Dependent Variable: lenticule
Model: (Intercept), group, eyes, group * eyes

a. Set to zero because this parameter is redundant.

```
 * Generalized Estimating Equations.
GENLIN RST BY group eyes (ORDER=ASCENDING)
  /MODEL group eyes group*eyes INTERCEPT=YES
 DISTRIBUTION=NORMAL LINK=IDENTITY
  /CRITERIA SCALE=MLE PCONVERGE=1E-006(ABSOLUTE) SINGULAR=1E-012 ANALYSISTY
PE=3(WALD) CILEVEL=95
    LIKELIHOOD=FULL
  /REPEATED SUBJECT=patients WITHINSUBJECT=eyes SORT=YES CORRTYPE=INDEPENDE
NT ADJUSTCORR=YES
    COVB=ROBUST
  /MISSING CLASSMISSING=EXCLUDE
  /PRINT CPS DESCRIPTIVES MODELINFO FIT SUMMARY SOLUTION.
```

Generalized Linear Models

Notes

Output Created		28-NOV-2023 15:47...
Comments		
Input	Data	/Users/yangshan/Desktop/2022-5-12/ /2023-10-OBL/2023-11-22 revised manuscript/OBL .sav
	Active Dataset	DataSet1
	Filter	<none>
	Weight	<none>
	Split File	<none>
	N of Rows in Working Data File	56
Missing Value Handling	Definition of Missing	User-defined missing values for factor, subject and within-subject variables are treated as missing.
	Cases Used	Statistics are based on cases with valid data for all variables in the model.
Weight Handling		not applicable

Notes

Syntax		GENLIN RST BY group eyes (ORDER=ASCENDING)<br>/MODEL group eyes group*eyes INTERCEPT=YES<br><br>DISTRIBUTION=NORMAL LINK=IDENTITY<br>/CRITERIA SCALE=MLE PCONVERGE=1E-006 (ABSOLUTE) SINGULAR=1E-012 ANALYSISTYPE=3(WALD) CILEVEL=95<br>LIKELIHOOD=FULL<br>/REPEATED SUBJECT=patients WITHINSUBJECT=eyes SORT=YES CORRTYPE=INDEPENDENT ADJUSTCORR=YES<br>COVB=ROBUST<br>/MISSING CLASSMISSING=EXCLUDE<br>/PRINT CPS DESCRIPTIVES MODELINFO FIT SUMMARY SOLUTION.
Resources	Processor Time	00:00:00.03
	Elapsed Time	00:00:00.00

Model Information

Dependent Variable		RST
Probability Distribution		Normal
Link Function		Identity
Subject Effect	1	patients
Within-Subject Effect	1	eyes
Working Correlation Matrix Structure		Independent

Case Processing Summary

	N	Percent
Included	56	100.0%
Excluded	0	0.0%
Total	56	100.0%

Correlated Data Summary

Number of Levels	Subject Effect	patients	32
	Within-Subject Effect	eyes	2
Number of Subjects			32
Number of Measurements per Subject	Minimum		1
	Maximum		2
Correlation Matrix Dimension			2

Categorical Variable Information

			N	Percent
Factor	group	1	29	51.8%
		2	27	48.2%
		Total	56	100.0%
	eyes	1	32	57.1%
		2	24	42.9%
		Total	56	100.0%

Continuous Variable Information

		N	Minimum	Maximum	Mean	Std. Deviation
Dependent Variable	RST	56	280.00	378.00	302.5893	25.45711

Goodness of Fit[a]

	Value
Quasi Likelihood under Independence Model Criterion (QIC)[b]	32992.663
Corrected Quasi Likelihood under Independence Model Criterion (QICC)[b]	32992.591

Dependent Variable: RST
Model: (Intercept), group, eyes, group * eyes[a]

a. Information criteria are in smaller-is-better form.

b. Computed using the full log quasi-likelihood function.

Tests of Model Effects

Source	Type III		
	Wald Chi-Square	df	Sig.
(Intercept)	4761.170	1	.000
group	2.388	1	.122
eyes	.844	1	.358
group * eyes	1.284	1	.257

Dependent Variable: RST
Model: (Intercept), group, eyes, group * eyes

Parameter Estimates

Parameter	B	Std. Error	95% Wald Confidence Interval		Hypothesis ...
			Lower	Upper	Wald Chi-Square
(Intercept)	295.500	6.0145	283.712	307.288	2413.921
[group=1]	17.250	10.2453	-2.830	37.330	2.835
[group=2]	0[a]	.	.	.	.
[eyes=1]	.700	3.1967	-5.565	6.965	.048
[eyes=2]	0[a]	.	.	.	.
[group=1] * [eyes=1]	-7.391	6.5219	-20.174	5.392	1.284
[group=1] * [eyes=2]	0[a]	.	.	.	.
[group=2] * [eyes=1]	0[a]	.	.	.	.
[group=2] * [eyes=2]	0[a]	.	.	.	.
(Scale)	634.319				

Parameter Estimates

Parameter	Hypothesis Test	
	df	Sig.
(Intercept)	1	.000
[group=1]	1	.092
[group=2]	.	.
[eyes=1]	1	.827
[eyes=2]	.	.
[group=1] * [eyes=1]	1	.257
[group=1] * [eyes=2]	.	.
[group=2] * [eyes=1]	.	.
[group=2] * [eyes=2]	.	.
(Scale)		

Dependent Variable: RST
Model: (Intercept), group, eyes, group * eyes

a. Set to zero because this parameter is redundant.

```
 MIXED sphere WITH OBL
  /CRITERIA=DFMETHOD(SATTERTHWAITE) CIN(95) MXITER(100) MXSTEP(10) SCORING(
1)
    SINGULAR(0.000000000001) HCONVERGE(0, ABSOLUTE) LCONVERGE(0, ABSOLUTE)
PCONVERGE(0.000001, ABSOLUTE)
  /FIXED=OBL | SSTYPE(3)
  /METHOD=ML
  /PRINT=COVB  SOLUTION
  /RANDOM=INTERCEPT | SUBJECT(patients) COVTYPE(VC)
  /REPEATED=eyes | SUBJECT(patients) COVTYPE(CS).
```

Mixed Model Analysis

Notes

Output Created		28-NOV-2023 15:50...
Comments		
Input	Data	/Users/yangshan/Desktop/2022-5-12/ /2023-10-OBL/2023-11-22 revised manuscript/OBL.sav
	Active Dataset	DataSet1
	Filter	<none>
	Weight	<none>
	Split File	<none>
	N of Rows in Working Data File	56
Missing Value Handling	Definition of Missing	User-defined missing values are treated as missing.
	Cases Used	Statistics are based on all cases with valid data for all variables in the model.

Notes

Syntax		MIXED sphere WITH OBL<br><br>/CRITERIA=DFMETHOD (SATTERTHWAITE) CIN (95) MXITER(100) MXSTEP(10) SCORING(1) SINGULAR (0.000000000001) HCONVERGE(0, ABSOLUTE) LCONVERGE (0, ABSOLUTE) PCONVERGE(0.000001, ABSOLUTE)<br>/FIXED=OBL | SSTYPE (3)<br>/METHOD=ML<br>/PRINT=COVB SOLUTION<br><br>/RANDOM=INTERCEPT | SUBJECT(patients) COVTYPE(VC)<br>/REPEATED=eyes | SUBJECT(patients) COVTYPE(CS).
Resources	Processor Time	00:00:00.02
	Elapsed Time	00:00:00.00

Warnings

Iteration was terminated but convergence has not been achieved. The MIXED procedure continues despite this warning. Subsequent results produced are based on the last iteration. Validity of the model fit is uncertain.

Model Dimension[a]

		Number of Levels	Covariance Structure	Number of Parameters	Subject Variables
Fixed Effects	Intercept	1		1	
	OBL	1		1	
Random Effects	Intercept[b]	1	Variance Components	1	patients
Repeated Effects	eyes	2	Compound Symmetry	2	patients
Total		5		5	

Model Dimension[a]

		Number of Subjects
Fixed Effects	Intercept	
	OBL	
Random Effects	Intercept[b]	
Repeated Effects	eyes	32
Total		

a. Dependent Variable: sphere.

b. As of version 11.5, the syntax rules for the RANDOM subcommand have changed. Your command syntax may yield results that differ from those produced by prior versions. If you are using version 11 syntax, please consult the current syntax reference guide for more...

Information Criteria[a]

-2 Log Likelihood	188.582
Akaike's Information Criterion (AIC)	198.582
Hurvich and Tsai's Criterion (AICC)	199.782
Bozdogan's Criterion (CAIC)	213.709
Schwarz's Bayesian Criterion (BIC)	208.709

The information criteria are displayed in smaller-is-better form.

a. Dependent Variable: sphere.

Fixed Effects

Type III Tests of Fixed Effects[a]

Source	Numerator df	Denominator df	F	Sig.
Intercept	1	47.556	136.845	.000
OBL	1	51.187	6.510	.014

a. Dependent Variable: sphere.

Estimates of Fixed Effects[a]

Parameter	Estimate	Std. Error	df	t	Sig.	95% ... Lower Bound
Intercept	-6.764453	.578254	47.556	-11.698	.000	-7.927391
OBL	.479281	.187843	51.187	2.552	.014	.102204

Estimates of Fixed Effects[a]

Parameter	95% Confidence . Upper Bound
Intercept	-5.601515
OBL	.856357

a. Dependent Variable: sphere.

Covariance Matrix for Estimates of Fixed Effects[a]

Parameter	Intercept	OBL
Intercept	.334378	-.097043
OBL	-.097043	.035285

a. Dependent Variable: sphere.

Covariance Parameters

Estimates of Covariance Parameters[a]

Parameter		Estimate	Std. Error
Repeated Measures	CS diagonal offset	.615070	.178720
	CS covariance	.591069	.554656
Intercept [subject = patients]	Variance	1.191572[b]	.000000

a. Dependent Variable: sphere.

b. This covariance parameter is redundant.

Covariance Matrix for Estimates of Covariance Parameters[a]

Parameter		Repeated Measures CS diagonal offset	Repeated Measures CS covariance	Intercept [subject = patients] Variance
Repeated Measures	CS diagonal offset	.031941	-.020659	.000000
	CS covariance	-.020659	.307643	.000000
Intercept [subject = patients]	Variance	.000000	.000000	.000000

a. Dependent Variable: sphere.

```
 MIXED Km WITH OBL
  /CRITERIA=DFMETHOD(SATTERTHWAITE) CIN(95) MXITER(100) MXSTEP(10) SCORING(
1)
    SINGULAR(0.000000000001) HCONVERGE(0, ABSOLUTE) LCONVERGE(0, ABSOLUTE)
PCONVERGE(0.000001, ABSOLUTE)
  /FIXED=OBL | SSTYPE(3)
  /METHOD=ML
  /PRINT=COVB  SOLUTION
  /RANDOM=INTERCEPT | SUBJECT(patients) COVTYPE(VC)
  /REPEATED=eyes | SUBJECT(patients) COVTYPE(CS).
```

Mixed Model Analysis

Notes

Output Created		28-NOV-2023 15:50...
Comments		
Input	Data	/Users/yangshan/Desktop/2022-5-12/ /2023-10-OBL/2023-11-22 revised manuscript/OBL .sav
	Active Dataset	DataSet1
	Filter	<none>
	Weight	<none>
	Split File	<none>
	N of Rows in Working Data File	56
Missing Value Handling	Definition of Missing	User-defined missing values are treated as missing.
	Cases Used	Statistics are based on all cases with valid data for all variables in the model.

Notes

Syntax		MIXED Km WITH OBL<br><br>/CRITERIA=DFMETHOD (SATTERTHWAITE) CIN (95) MXITER(100) MXSTEP(10) SCORING(1) SINGULAR (0.000000000001) HCONVERGE(0, ABSOLUTE) LCONVERGE (0, ABSOLUTE) PCONVERGE(0.000001, ABSOLUTE)<br>/FIXED=OBL | SSTYPE (3)<br>/METHOD=ML<br>/PRINT=COVB SOLUTION<br><br>/RANDOM=INTERCEPT | SUBJECT(patients) COVTYPE(VC)<br>/REPEATED=eyes | SUBJECT(patients) COVTYPE(CS).
Resources	Processor Time	00:00:00.01
	Elapsed Time	00:00:00.00

Warnings

Iteration was terminated but convergence has not been achieved. The MIXED procedure continues despite this warning. Subsequent results produced are based on the last iteration. Validity of the model fit is uncertain.

Model Dimension[a]

		Number of Levels	Covariance Structure	Number of Parameters	Subject Variables
Fixed Effects	Intercept	1		1	
	OBL	1		1	
Random Effects	Intercept[b]	1	Variance Components	1	patients
Repeated Effects	eyes	2	Compound Symmetry	2	patients
Total		5		5	

Model Dimension[a]

		Number of Subjects
Fixed Effects	Intercept	
	OBL	
Random Effects	Intercept[b]	
Repeated Effects	eyes	32
Total		

a. Dependent Variable: Km.

b. As of version 11.5, the syntax rules for the RANDOM subcommand have changed. Your command syntax may yield results that differ from those produced by prior versions. If you are using version 11 syntax, please consult the current syntax reference guide for more...

Information Criteria[a]

-2 Log Likelihood	121.447
Akaike's Information Criterion (AIC)	131.447
Hurvich and Tsai's Criterion (AICC)	132.647
Bozdogan's Criterion (CAIC)	146.573
Schwarz's Bayesian Criterion (BIC)	141.573

The information criteria are displayed in smaller-is-better form.

a. Dependent Variable: Km.

Fixed Effects

Type III Tests of Fixed Effects[a]

Source	Numerator df	Denominator df	F	Sig.
Intercept	1	56.000	16969.746	.000
OBL	1	46.951	4.069	.049

a. Dependent Variable: Km.

Estimates of Fixed Effects[a]

Parameter	Estimate	Std. Error	df	t	Sig.	95% ...
						Lower Bound
Intercept	44.083036	.338403	56.000	130.268	.000	43.405134
OBL	-.203780	.101027	46.951	-2.017	.049	-.407025

Estimates of Fixed Effects[a]

Parameter	95% Confidence .
	Upper Bound
Intercept	44.760938
OBL	-.000535

a. Dependent Variable: Km.

Covariance Matrix for Estimates of Fixed Effects[a]

Parameter	Intercept	OBL
Intercept	.114516	-.028060
OBL	-.028060	.010206

a. Dependent Variable: Km.

Covariance Parameters

Estimates of Covariance Parameters[a]

Parameter		Estimate	Std. Error
Repeated Measures	CS diagonal offset	.082639	.024417
	CS covariance	.451199	.305248
Intercept [subject = patients]	Variance	.693332[b]	.000000

a. Dependent Variable: Km.

b. This covariance parameter is redundant.

Covariance Matrix for Estimates of Covariance Parameters[a]

Parameter		Repeated Measures		Intercept [subject = patients]
		CS diagonal offset	CS covariance	Variance
Repeated Measures	CS diagonal offset	.000596	-.000665	.000000
	CS covariance	-.000665	.093177	.000000
Intercept [subject = patients]	Variance	.000000	.000000	.000000

a. Dependent Variable: Km.

```
 MIXED CCT WITH OBL
  /CRITERIA=DFMETHOD(SATTERTHWAITE) CIN(95) MXITER(100) MXSTEP(10) SCORING(
1)
    SINGULAR(0.000000000001) HCONVERGE(0, ABSOLUTE) LCONVERGE(0, ABSOLUTE)
PCONVERGE(0.000001, ABSOLUTE)
  /FIXED=OBL | SSTYPE(3)
  /METHOD=ML
  /PRINT=COVB  SOLUTION
  /RANDOM=INTERCEPT | SUBJECT(patients) COVTYPE(VC)
  /REPEATED=eyes | SUBJECT(patients) COVTYPE(CS).
```

Mixed Model Analysis

Notes

Output Created		28-NOV-2023 15:51...
Comments		
Input	Data	/Users/yangshan/Desktop/2022-5-12//2023-10-OBL/2023-11-22 revised manuscript/OBL.sav
	Active Dataset	DataSet1
	Filter	<none>
	Weight	<none>
	Split File	<none>
	N of Rows in Working Data File	56
Missing Value Handling	Definition of Missing	User-defined missing values are treated as missing.
	Cases Used	Statistics are based on all cases with valid data for all variables in the model.

Notes

Syntax		MIXED CCT WITH OBL<br><br>/CRITERIA=DFMETHOD (SATTERTHWAITE) CIN (95) MXITER(100) MXSTEP(10) SCORING(1) SINGULAR (0.000000000001) HCONVERGE(0, ABSOLUTE) LCONVERGE (0, ABSOLUTE) PCONVERGE(0.000001, ABSOLUTE)<br>/FIXED=OBL | SSTYPE (3)<br>/METHOD=ML<br>/PRINT=COVB SOLUTION<br><br>/RANDOM=INTERCEPT | SUBJECT(patients) COVTYPE(VC)<br>/REPEATED=eyes | SUBJECT(patients) COVTYPE(CS).
Resources	Processor Time	00:00:00.01
	Elapsed Time	00:00:00.00

Warnings

Iteration was terminated but convergence has not been achieved. The MIXED procedure continues despite this warning. Subsequent results produced are based on the last iteration. Validity of the model fit is uncertain.

Model Dimension[a]

		Number of Levels	Covariance Structure	Number of Parameters	Subject Variables
Fixed Effects	Intercept	1		1	
	OBL	1		1	
Random Effects	Intercept[b]	1	Variance Components	1	patients
Repeated Effects	eyes	2	Compound Symmetry	2	patients
Total		5		5	

Model Dimension[a]

		Number of Subjects
Fixed Effects	Intercept	
	OBL	
Random Effects	Intercept[b]	
Repeated Effects	eyes	32
Total		

a. Dependent Variable: CCT.

b. As of version 11.5, the syntax rules for the RANDOM subcommand have changed. Your command syntax may yield results that differ from those produced by prior versions. If you are using version 11 syntax, please consult the current syntax reference guide for more...

Information Criteria[a]

-2 Log Likelihood	443.412
Akaike's Information Criterion (AIC)	453.412
Hurvich and Tsai's Criterion (AICC)	454.612
Bozdogan's Criterion (CAIC)	468.539
Schwarz's Bayesian Criterion (BIC)	463.539

The information criteria are displayed in smaller-is-better form.

a. Dependent Variable: CCT.

Fixed Effects

Type III Tests of Fixed Effects[a]

Source	Numerator df	Denominator df	F	Sig.
Intercept	1	55.968	7627.856	.000
OBL	1	38.739	.172	.681

a. Dependent Variable: CCT.

Estimates of Fixed Effects[a]

Parameter	Estimate	Std. Error	df	t	Sig.	95% ...
						Lower Bound
Intercept	522.066572	5.977569	55.968	87.338	.000	510.091912
OBL	.687518	1.657418	38.739	.415	.681	-2.665647

Estimates of Fixed Effects[a]

Parameter	95% Confidence .
	Upper Bound
Intercept	534.041232
OBL	4.040682

a. Dependent Variable: CCT.

Covariance Matrix for Estimates of Fixed Effects[a]

Parameter	Intercept	OBL
Intercept	35.731337	-7.551888
OBL	-7.551888	2.747033

a. Dependent Variable: CCT.

Covariance Parameters

Estimates of Covariance Parameters[a]

Parameter		Estimate	Std. Error
Repeated Measures	CS diagonal offset	18.756215	5.425964
	CS covariance	126.682792	120.014203
Intercept [subject = patients]	Variance	340.68115[b]	.000000

a. Dependent Variable: CCT.

b. This covariance parameter is redundant.

Covariance Matrix for Estimates of Covariance Parameters[a]

Parameter		Repeated Measures		Intercept [subject = patients]
		CS diagonal offset	CS covariance	Variance
Repeated Measures	CS diagonal offset	29.441089	-20.737601	.000000
	CS covariance	-20.737601	14403.4090	.000000
Intercept [subject = patients]	Variance	.000000	.000000	.000000

a. Dependent Variable: CCT.

```
 MIXED lenticule WITH OBL
  /CRITERIA=DFMETHOD(SATTERTHWAITE) CIN(95) MXITER(100) MXSTEP(10) SCORING(
1)
    SINGULAR(0.000000000001) HCONVERGE(0, ABSOLUTE) LCONVERGE(0, ABSOLUTE)
PCONVERGE(0.000001, ABSOLUTE)
  /FIXED=OBL | SSTYPE(3)
  /METHOD=ML
  /PRINT=COVB  SOLUTION
  /RANDOM=INTERCEPT | SUBJECT(patients) COVTYPE(VC)
  /REPEATED=eyes | SUBJECT(patients) COVTYPE(CS).
```

Mixed Model Analysis

Notes

Output Created		28-NOV-2023 15:51...
Comments		
Input	Data	/Users/yangshan/Desktop/2022-5-12/ /2023-10-OBL/2023-11-22 revised manuscript/OBL.sav
	Active Dataset	DataSet1
	Filter	<none>
	Weight	<none>
	Split File	<none>
	N of Rows in Working Data File	56
Missing Value Handling	Definition of Missing	User-defined missing values are treated as missing.
	Cases Used	Statistics are based on all cases with valid data for all variables in the model.

Notes

Syntax		MIXED lenticule WITH OBL<br><br>/CRITERIA=DFMETHOD (SATTERTHWAITE) CIN (95) MXITER(100) MXSTEP(10) SCORING(1) SINGULAR (0.000000000001) HCONVERGE(0, ABSOLUTE) LCONVERGE (0, ABSOLUTE) PCONVERGE(0.000001, ABSOLUTE)<br>/FIXED=OBL | SSTYPE (3)<br>/METHOD=ML<br>/PRINT=COVB SOLUTION<br><br>/RANDOM=INTERCEPT | SUBJECT(patients) COVTYPE(VC)<br>/REPEATED=eyes | SUBJECT(patients) COVTYPE(CS).
Resources	Processor Time	00:00:00.02
	Elapsed Time	00:00:00.00

Warnings

Iteration was terminated but convergence has not been achieved. The MIXED procedure continues despite this warning. Subsequent results produced are based on the last iteration. Validity of the model fit is uncertain.

Model Dimension[a]

		Number of Levels	Covariance Structure	Number of Parameters	Subject Variables
Fixed Effects	Intercept	1		1	
	OBL	1		1	
Random Effects	Intercept[b]	1	Variance Components	1	patients
Repeated Effects	eyes	2	Compound Symmetry	2	patients
Total		5		5	

Model Dimension[a]

		Number of Subjects
Fixed Effects	Intercept	
	OBL	
Random Effects	Intercept[b]	
Repeated Effects	eyes	32
Total		

a. Dependent Variable: lenticule.

b. As of version 11.5, the syntax rules for the RANDOM subcommand have changed. Your command syntax may yield results that differ from those produced by prior versions. If you are using version 11 syntax, please consult the current syntax reference guide for more...

Information Criteria[a]

-2 Log Likelihood	463.662
Akaike's Information Criterion (AIC)	473.662
Hurvich and Tsai's Criterion (AICC)	474.862
Bozdogan's Criterion (CAIC)	488.789
Schwarz's Bayesian Criterion (BIC)	483.789

The information criteria are displayed in smaller-is-better form.

a. Dependent Variable: lenticule.

Fixed Effects

Type III Tests of Fixed Effects[a]

Source	Numerator df	Denominator df	F	Sig.
Intercept	1	47.119	287.992	.000
OBL	1	50.403	2.170	.147

a. Dependent Variable: lenticule.

Estimates of Fixed Effects[a]

Parameter	Estimate	Std. Error	df	t	Sig.	95% ... Lower Bound
Intercept	113.408854	6.682778	47.119	16.970	.000	99.965737
OBL	-3.204999	2.175671	50.403	-1.473	.147	-7.574097

Estimates of Fixed Effects[a]

Parameter	95% Confidence . Upper Bound
Intercept	126.851970
OBL	1.164100

a. Dependent Variable: lenticule.

Covariance Matrix for Estimates of Fixed Effects[a]

Parameter	Intercept	OBL
Intercept	44.659523	-13.019082
OBL	-13.019082	4.733546

a. Dependent Variable: lenticule.

Covariance Parameters

Estimates of Covariance Parameters[a]

Parameter		Estimate	Std. Error
Repeated Measures	CS diagonal offset	87.635492	24.910591
	CS covariance	70.114673	71.240437
Intercept [subject = patients]	Variance	159.55263 [b]	.000000

a. Dependent Variable: lenticule.

b. This covariance parameter is redundant.

Covariance Matrix for Estimates of Covariance Parameters[a]

Parameter		Repeated Measures CS diagonal offset	Repeated Measures CS covariance	Intercept [subject = patients] Variance
Repeated Measures	CS diagonal offset	620.537548	-332.33901	.000000
	CS covariance	-332.33901	5075.19993	.000000
Intercept [subject = patients]	Variance	.000000	.000000	.000000

a. Dependent Variable: lenticule.

```
 MIXED RST WITH OBL
  /CRITERIA=DFMETHOD(SATTERTHWAITE) CIN(95) MXITER(100) MXSTEP(10) SCORING(
1)
    SINGULAR(0.000000000001) HCONVERGE(0, ABSOLUTE) LCONVERGE(0, ABSOLUTE)
PCONVERGE(0.000001, ABSOLUTE)
  /FIXED=OBL | SSTYPE(3)
  /METHOD=ML
  /PRINT=COVB  SOLUTION
  /RANDOM=INTERCEPT | SUBJECT(patients) COVTYPE(VC)
  /REPEATED=eyes | SUBJECT(patients) COVTYPE(CS).
```

Mixed Model Analysis

Notes

Output Created		28-NOV-2023 15:52...
Comments		
Input	Data	/Users/yangshan/Desktop/2022-5-12/ /2023-10-OBL/2023-11-22 revised manuscript/OBL.sav
	Active Dataset	DataSet1
	Filter	<none>
	Weight	<none>
	Split File	<none>
	N of Rows in Working Data File	56
Missing Value Handling	Definition of Missing	User-defined missing values are treated as missing.
	Cases Used	Statistics are based on all cases with valid data for all variables in the model.

Notes

Syntax		MIXED RST WITH OBL<br><br>/CRITERIA=DFMETHOD (SATTERTHWAITE) CIN (95) MXITER(100) MXSTEP(10) SCORING(1) SINGULAR (0.000000000001) HCONVERGE(0, ABSOLUTE) LCONVERGE (0, ABSOLUTE) PCONVERGE(0.000001, ABSOLUTE)<br>/FIXED=OBL | SSTYPE (3)<br>/METHOD=ML<br>/PRINT=COVB SOLUTION<br><br>/RANDOM=INTERCEPT | SUBJECT(patients) COVTYPE(VC)<br>/REPEATED=eyes | SUBJECT(patients) COVTYPE(CS).
Resources	Processor Time	00:00:00.02
	Elapsed Time	00:00:00.00

Warnings

Iteration was terminated but convergence has not been achieved. The MIXED procedure continues despite this warning. Subsequent results produced are based on the last iteration. Validity of the model fit is uncertain.

Model Dimension[a]

		Number of Levels	Covariance Structure	Number of Parameters	Subject Variables
Fixed Effects	Intercept	1		1	
	OBL	1		1	
Random Effects	Intercept[b]	1	Variance Components	1	patients
Repeated Effects	eyes	2	Compound Symmetry	2	patients
Total		5		5	

Model Dimension[a]

		Number of Subjects
Fixed Effects	Intercept	
	OBL	
Random Effects	Intercept[b]	
Repeated Effects	eyes	32
Total		

a. Dependent Variable: RST.

b. As of version 11.5, the syntax rules for the RANDOM subcommand have changed. Your command syntax may yield results that differ from those produced by prior versions. If you are using version 11 syntax, please consult the current syntax reference guide for more...

Information Criteria[a]

-2 Log Likelihood	480.735
Akaike's Information Criterion (AIC)	490.735
Hurvich and Tsai's Criterion (AICC)	491.935
Bozdogan's Criterion (CAIC)	505.862
Schwarz's Bayesian Criterion (BIC)	500.862

The information criteria are displayed in smaller-is-better form.

a. Dependent Variable: RST.

Fixed Effects

Type III Tests of Fixed Effects[a]

Source	Numerator df	Denominator df	F	Sig.
Intercept	1	54.377	1221.700	.000
OBL	1	55.867	2.818	.099

a. Dependent Variable: RST.

Estimates of Fixed Effects[a]

Parameter	Estimate	Std. Error	df	t	Sig.	95% ... Lower Bound	95% Confidence . Upper Bound
Intercept	288.537833	8.255064	54.377	34.953	.000	271.990052	305.085613
OBL	4.377173	2.607721	55.867	1.679	.099	-.846993	9.601339

a. Dependent Variable: RST.

Covariance Matrix for Estimates of Fixed Effects[a]

Parameter	Intercept	OBL
Intercept	68.146085	-18.697985
OBL	-18.697985	6.800208

a. Dependent Variable: RST.

Covariance Parameters

Estimates of Covariance Parameters[a]

Parameter		Estimate	Std. Error
Repeated Measures	CS diagonal offset	76.524139	21.915187
	CS covariance	203.576925	133.576068
Intercept [subject = patients]	Variance	284.56953[b]	.000000

a. Dependent Variable: RST.

b. This covariance parameter is redundant.

Covariance Matrix for Estimates of Covariance Parameters[a]

Parameter		Repeated Measures CS diagonal offset	Repeated Measures CS covariance	Intercept [subject = patients] Variance
Repeated Measures	CS diagonal offset	480.275432	-256.55410	.000000
	CS covariance	-256.55410	17842.5659	.000000
Intercept [subject = patients]	Variance	.000000	.000000	.000000

a. Dependent Variable: RST.
