# Supplementary material for: Possible risk factors of opaque bubble layer and its effect on high-order aberrations after small incision Lenticule extraction
Source: Front Med (Lausanne). 2023 Dec 20;10:1156677. doi: 10.3389/fmed.2023.1156677 (PMC10765512; doi:10.3389/fmed.2023.1156677)
Supplement: Supplementary file 2 [file Data_Sheet_2.PDF]

```
DATASET ACTIVATE DataSet1.

SAVE OUTFILE='/Users/yangshan/Desktop/2022-5-12/    /2023-10-OBL/2023-11-
22 revised '+
    'manuscript/OBL       .sav'
  /COMPRESSED.
* Generalized Estimating Equations.
GENLIN tHOAs3 BY group eyes (ORDER=ASCENDING)
  /MODEL group eyes group*eyes INTERCEPT=YES
 DISTRIBUTION=NORMAL LINK=IDENTITY
  /CRITERIA SCALE=MLE PCONVERGE=1E-006(ABSOLUTE) SINGULAR=1E-
012 ANALYSISTYPE=3(WALD) CILEVEL=95
    LIKELIHOOD=FULL
  /REPEATED SUBJECT=patients WITHINSUBJECT=eyes SORT=YES CORRTYPE=INDEPENDE
NT ADJUSTCORR=YES
    COVB=ROBUST
  /MISSING CLASSMISSING=EXCLUDE
  /PRINT CPS DESCRIPTIVES MODELINFO FIT SUMMARY SOLUTION.
```

Generalized Linear Models

Notes

Output Created		29-NOV-2023 19:51...
Comments		
Input	Data	/Users/yangshan/Desktop/2022-5-12/ /2023-10-OBL/2023-11-22 revised manuscript/OBL .sav
	Active Dataset	DataSet1
	Filter	<none>
	Weight	<none>
	Split File	<none>
	N of Rows in Working Data File	56
Missing Value Handling	Definition of Missing	User-defined missing values for factor, subject and within-subject variables are treated as missing.
	Cases Used	Statistics are based on cases with valid data for all variables in the model.
Weight Handling		not applicable

Notes

Syntax		GENLIN tHOAs3 BY group eyes (ORDER=ASCENDING)<br>/MODEL group eyes group*eyes INTERCEPT=YES<br><br>DISTRIBUTION=NORMAL LINK=IDENTITY<br>/CRITERIA SCALE=MLE PCONVERGE=1E-006 (ABSOLUTE) SINGULAR=1E-012 ANALYSISTYPE=3(WALD) CILEVEL=95<br>LIKELIHOOD=FULL<br>/REPEATED SUBJECT=patients WITHINSUBJECT=eyes SORT=YES CORRTYPE=INDEPENDENT ADJUSTCORR=YES<br>COVB=ROBUST<br>/MISSING CLASSMISSING=EXCLUDE<br>/PRINT CPS DESCRIPTIVES MODELINFO FIT SUMMARY SOLUTION.
Resources	Processor Time	00:00:00.05
	Elapsed Time	00:00:00.00

Model Information

Dependent Variable		tHOAs3
Probability Distribution		Normal
Link Function		Identity
Subject Effect	1	patients
Within-Subject Effect	1	eyes
Working Correlation Matrix Structure		Independent

Case Processing Summary

	N	Percent
Included	55	98.2%
Excluded	1	1.8%
Total	56	100.0%

Correlated Data Summary

Number of Levels	Subject Effect	patients	31
	Within-Subject Effect	eyes	2
Number of Subjects			31
Number of Measurements per Subject	Minimum		1
	Maximum		2
Correlation Matrix Dimension			2

Categorical Variable Information

			N	Percent
Factor	group	1	28	50.9%
		2	27	49.1%
		Total	55	100.0%
	eyes	1	31	56.4%
		2	24	43.6%
		Total	55	100.0%

Continuous Variable Information

		N	Minimum	Maximum	Mean	Std. Deviation
Dependent Variable	tHOAs3	55	.01	.14	.0525	.02898

Goodness of Fit[a]

	Value
Quasi Likelihood under Independence Model Criterion (QIC)[b]	8.210
Corrected Quasi Likelihood under Independence Model Criterion (QICC)[b]	8.043

Dependent Variable: tHOAs3
Model: (Intercept), group, eyes, group * eyes[a]

a. Information criteria are in smaller-is-better form.

b. Computed using the full log quasi-likelihood function.

Tests of Model Effects

Source	Type III		
	Wald Chi-Square	df	Sig.
(Intercept)	159.688	1	.000
group	1.178	1	.278
eyes	1.437	1	.231
group * eyes	1.053	1	.305

Dependent Variable: tHOAs3
Model: (Intercept), group, eyes, group * eyes

Parameter Estimates

Parameter	B	Std. Error	95% Wald Confidence Interval		Hypothesis ...
			Lower	Upper	Wald Chi-Square
(Intercept)	.049	.0085	.032	.066	33.613
[group=1]	.016	.0128	-.009	.041	1.639
[group=2]	0[a]	.	.	.	.
[eyes=1]	-.001	.0085	-.018	.015	.020
[eyes=2]	0[a]	.	.	.	.
[group=1] * [eyes=1]	-.014	.0141	-.042	.013	1.053
[group=1] * [eyes=2]	0[a]	.	.	.	.
[group=2] * [eyes=1]	0[a]	.	.	.	.
[group=2] * [eyes=2]	0[a]	.	.	.	.
(Scale)	.001				

Parameter Estimates

Parameter	Hypothesis Test	
	df	Sig.
(Intercept)	1	.000
[group=1]	1	.201
[group=2]	.	.
[eyes=1]	1	.887
[eyes=2]	.	.
[group=1] * [eyes=1]	1	.305
[group=1] * [eyes=2]	.	.
[group=2] * [eyes=1]	.	.
[group=2] * [eyes=2]	.	.
(Scale)		

Dependent Variable: tHOAs3
Model: (Intercept), group, eyes, group * eyes

a. Set to zero because this parameter is redundant.

```
 * Generalized Estimating Equations.
GENLIN Coma3 BY group eyes (ORDER=ASCENDING)
  /MODEL group eyes group*eyes INTERCEPT=YES
 DISTRIBUTION=NORMAL LINK=IDENTITY
  /CRITERIA SCALE=MLE PCONVERGE=1E-006(ABSOLUTE) SINGULAR=1E-012 ANALYSISTY
PE=3(WALD) CILEVEL=95
    LIKELIHOOD=FULL
  /REPEATED SUBJECT=patients WITHINSUBJECT=eyes SORT=YES CORRTYPE=INDEPENDE
NT ADJUSTCORR=YES
    COVB=ROBUST
  /MISSING CLASSMISSING=EXCLUDE
  /PRINT CPS DESCRIPTIVES MODELINFO FIT SUMMARY SOLUTION.
```

Generalized Linear Models

Notes

Output Created		29-NOV-2023 19:51...
Comments		
Input	Data	/Users/yangshan/Desktop/2022-5-12//2023-10-OBL/2023-11-22 revised manuscript/OBL.sav
	Active Dataset	DataSet1
	Filter	<none>
	Weight	<none>
	Split File	<none>
	N of Rows in Working Data File	56
Missing Value Handling	Definition of Missing	User-defined missing values for factor, subject and within-subject variables are treated as missing.
	Cases Used	Statistics are based on cases with valid data for all variables in the model.
Weight Handling		not applicable

Notes

Syntax		GENLIN Coma3 BY group eyes (ORDER=ASCENDING)<br>/MODEL group eyes group*eyes INTERCEPT=YES<br><br>DISTRIBUTION=NORMAL LINK=IDENTITY<br>/CRITERIA SCALE=MLE PCONVERGE=1E-006 (ABSOLUTE) SINGULAR=1E-012 ANALYSISTYPE=3(WALD) CILEVEL=95<br>LIKELIHOOD=FULL<br>/REPEATED SUBJECT=patients WITHINSUBJECT=eyes SORT=YES CORRTYPE=INDEPENDENT ADJUSTCORR=YES<br>COVB=ROBUST<br>/MISSING CLASSMISSING=EXCLUDE<br>/PRINT CPS DESCRIPTIVES MODELINFO FIT SUMMARY SOLUTION.
Resources	Processor Time	00:00:00.04
	Elapsed Time	00:00:00.00

Model Information

Dependent Variable		Coma3
Probability Distribution		Normal
Link Function		Identity
Subject Effect	1	patients
Within-Subject Effect	1	eyes
Working Correlation Matrix Structure		Independent

Case Processing Summary

	N	Percent
Included	55	98.2%
Excluded	1	1.8%
Total	56	100.0%

Correlated Data Summary

Number of Levels	Subject Effect	patients	31
	Within-Subject Effect	eyes	2
Number of Subjects			31
Number of Measurements per Subject	Minimum		1
	Maximum		2
Correlation Matrix Dimension			2

Categorical Variable Information

			N	Percent
Factor	group	1	28	50.9%
		2	27	49.1%
		Total	55	100.0%
	eyes	1	31	56.4%
		2	24	43.6%
		Total	55	100.0%

Continuous Variable Information

		N	Minimum	Maximum	Mean	Std. Deviation
Dependent Variable	Coma3	55	.00	.12	.0310	.02338

Goodness of Fit[a]

	Value
Quasi Likelihood under Independence Model Criterion (QIC)[b]	8.274
Corrected Quasi Likelihood under Independence Model Criterion (QICC)[b]	8.028

Dependent Variable: Coma3
Model: (Intercept), group, eyes, group * eyes[a]

a. Information criteria are in smaller-is-better form.

b. Computed using the full log quasi-likelihood function.

Tests of Model Effects

Source	Type III		
	Wald Chi-Square	df	Sig.
(Intercept)	83.006	1	.000
group	.643	1	.423
eyes	2.212	1	.137
group * eyes	.549	1	.459

Dependent Variable: Coma3
Model: (Intercept), group, eyes, group * eyes

Parameter Estimates

Parameter	B	Std. Error	95% Wald Confidence Interval		Hypothesis ...
			Lower	Upper	Wald Chi-Square
(Intercept)	.031	.0067	.018	.044	21.479
[group=1]	.010	.0106	-.011	.031	.845
[group=2]	0[a]	.	.	.	.
[eyes=1]	-.004	.0062	-.016	.008	.463
[eyes=2]	0[a]	.	.	.	.
[group=1] * [eyes=1]	-.008	.0113	-.031	.014	.549
[group=1] * [eyes=2]	0[a]	.	.	.	.
[group=2] * [eyes=1]	0[a]	.	.	.	.
[group=2] * [eyes=2]	0[a]	.	.	.	.
(Scale)	.001				

Parameter Estimates

Parameter	Hypothesis Test	
	df	Sig.
(Intercept)	1	.000
[group=1]	1	.358
[group=2]	.	.
[eyes=1]	1	.496
[eyes=2]	.	.
[group=1] * [eyes=1]	1	.459
[group=1] * [eyes=2]	.	.
[group=2] * [eyes=1]	.	.
[group=2] * [eyes=2]	.	.
(Scale)		

Dependent Variable: Coma3
Model: (Intercept), group, eyes, group * eyes

a. Set to zero because this parameter is redundant.

```
 * Generalized Estimating Equations.
GENLIN SA3 BY group eyes (ORDER=ASCENDING)
  /MODEL group eyes group*eyes INTERCEPT=YES
 DISTRIBUTION=NORMAL LINK=IDENTITY
  /CRITERIA SCALE=MLE PCONVERGE=1E-006(ABSOLUTE) SINGULAR=1E-012 ANALYSISTY
PE=3(WALD) CILEVEL=95
    LIKELIHOOD=FULL
  /REPEATED SUBJECT=patients WITHINSUBJECT=eyes SORT=YES CORRTYPE=INDEPENDE
NT ADJUSTCORR=YES
    COVB=ROBUST
  /MISSING CLASSMISSING=EXCLUDE
  /PRINT CPS DESCRIPTIVES MODELINFO FIT SUMMARY SOLUTION.
```

Generalized Linear Models

Notes

Output Created		29-NOV-2023 19:51...
Comments		
Input	Data	/Users/yangshan/Desktop/2022-5-12//2023-10-OBL/2023-11-22 revised manuscript/OBL.sav
	Active Dataset	DataSet1
	Filter	<none>
	Weight	<none>
	Split File	<none>
	N of Rows in Working Data File	56
Missing Value Handling	Definition of Missing	User-defined missing values for factor, subject and within-subject variables are treated as missing.
	Cases Used	Statistics are based on cases with valid data for all variables in the model.
Weight Handling		not applicable

Notes

Syntax		GENLIN SA3 BY group eyes (ORDER=ASCENDING)<br>/MODEL group eyes group*eyes INTERCEPT=YES<br><br>DISTRIBUTION=NORMAL LINK=IDENTITY<br>/CRITERIA SCALE=MLE PCONVERGE=1E-006 (ABSOLUTE) SINGULAR=1E-012 ANALYSISTYPE=3(WALD) CILEVEL=95<br>LIKELIHOOD=FULL<br>/REPEATED SUBJECT=patients WITHINSUBJECT=eyes SORT=YES CORRTYPE=INDEPENDENT ADJUSTCORR=YES<br>COVB=ROBUST<br>/MISSING CLASSMISSING=EXCLUDE<br>/PRINT CPS DESCRIPTIVES MODELINFO FIT SUMMARY SOLUTION.
Resources	Processor Time	00:00:00.04
	Elapsed Time	00:00:00.00

Model Information

Dependent Variable		SA3
Probability Distribution		Normal
Link Function		Identity
Subject Effect	1	patients
Within-Subject Effect	1	eyes
Working Correlation Matrix Structure		Independent

Case Processing Summary

	N	Percent
Included	52	92.9%
Excluded	4	7.1%
Total	56	100.0%

Correlated Data Summary

Number of Levels	Subject Effect	patients	32
	Within-Subject Effect	eyes	2
Number of Subjects			32
Number of Measurements per Subject	Minimum		1
	Maximum		2
Correlation Matrix Dimension			2

Categorical Variable Information

			N	Percent
Factor	group	1	26	50.0%
		2	26	50.0%
		Total	52	100.0%
	eyes	1	31	59.6%
		2	21	40.4%
		Total	52	100.0%

Continuous Variable Information

		N	Minimum	Maximum	Mean	Std. Deviation
Dependent Variable	SA3	52	.00	.11	.0439	.02634

Goodness of Fit[a]

	Value
Quasi Likelihood under Independence Model Criterion (QIC)[b]	8.179
Corrected Quasi Likelihood under Independence Model Criterion (QICC)[b]	8.034

Dependent Variable: SA3
Model: (Intercept), group, eyes, group * eyes[a]

a. Information criteria are in smaller-is-better form.

b. Computed using the full log quasi-likelihood function.

Tests of Model Effects

Source	Type III		
	Wald Chi-Square	df	Sig.
(Intercept)	148.228	1	.000
group	.021	1	.884
eyes	.025	1	.874
group * eyes	2.443	1	.118

Dependent Variable: SA3
Model: (Intercept), group, eyes, group * eyes

Parameter Estimates

Parameter	B	Std. Error	95% Wald Confidence Interval		Hypothesis ...
			Lower	Upper	Wald Chi-Square
(Intercept)	.051	.0081	.035	.067	39.293
[group=1]	-.013	.0119	-.036	.011	1.148
[group=2]	0[a]	.	.	.	.
[eyes=1]	-.013	.0091	-.031	.005	1.993
[eyes=2]	0[a]	.	.	.	.
[group=1] * [eyes=1]	.023	.0149	-.006	.053	2.443
[group=1] * [eyes=2]	0[a]	.	.	.	.
[group=2] * [eyes=1]	0[a]	.	.	.	.
[group=2] * [eyes=2]	0[a]	.	.	.	.
(Scale)	.001				

Parameter Estimates

Parameter	Hypothesis Test	
	df	Sig.
(Intercept)	1	.000
[group=1]	1	.284
[group=2]	.	.
[eyes=1]	1	.158
[eyes=2]	.	.
[group=1] * [eyes=1]	1	.118
[group=1] * [eyes=2]	.	.
[group=2] * [eyes=1]	.	.
[group=2] * [eyes=2]	.	.
(Scale)		

Dependent Variable: SA3
Model: (Intercept), group, eyes, group * eyes

a. Set to zero because this parameter is redundant.

```
 * Generalized Estimating Equations.
GENLIN Trefoil3 BY group eyes (ORDER=ASCENDING)
  /MODEL group eyes group*eyes INTERCEPT=YES
 DISTRIBUTION=NORMAL LINK=IDENTITY
  /CRITERIA SCALE=MLE PCONVERGE=1E-006(ABSOLUTE) SINGULAR=1E-012 ANALYSISTY
PE=3(WALD) CILEVEL=95
    LIKELIHOOD=FULL
  /REPEATED SUBJECT=patients WITHINSUBJECT=eyes SORT=YES CORRTYPE=INDEPENDE
NT ADJUSTCORR=YES
    COVB=ROBUST
  /MISSING CLASSMISSING=EXCLUDE
  /PRINT CPS DESCRIPTIVES MODELINFO FIT SUMMARY SOLUTION.
```

Generalized Linear Models

Notes

Output Created		29-NOV-2023 19:51...
Comments		
Input	Data	/Users/yangshan/Desktop/2022-5-12//2023-10-OBL/2023-11-22 revised manuscript/OBL.sav
	Active Dataset	DataSet1
	Filter	<none>
	Weight	<none>
	Split File	<none>
	N of Rows in Working Data File	56
Missing Value Handling	Definition of Missing	User-defined missing values for factor, subject and within-subject variables are treated as missing.
	Cases Used	Statistics are based on cases with valid data for all variables in the model.
Weight Handling		not applicable

Notes

Syntax		GENLIN Trefoil3 BY group eyes (ORDER=ASCENDING)<br>/MODEL group eyes group*eyes INTERCEPT=YES<br><br>DISTRIBUTION=NORMAL LINK=IDENTITY<br>/CRITERIA SCALE=MLE PCONVERGE=1E-006 (ABSOLUTE) SINGULAR=1E-012 ANALYSISTYPE=3(WALD) CILEVEL=95<br>LIKELIHOOD=FULL<br>/REPEATED SUBJECT=patients WITHINSUBJECT=eyes SORT=YES CORRTYPE=INDEPENDENT ADJUSTCORR=YES<br>COVB=ROBUST<br>/MISSING CLASSMISSING=EXCLUDE<br>/PRINT CPS DESCRIPTIVES MODELINFO FIT SUMMARY SOLUTION.
Resources	Processor Time	00:00:00.04
	Elapsed Time	00:00:00.00

Model Information

Dependent Variable		Trefoil3
Probability Distribution		Normal
Link Function		Identity
Subject Effect	1	patients
Within-Subject Effect	1	eyes
Working Correlation Matrix Structure		Independent

Case Processing Summary

	N	Percent
Included	55	98.2%
Excluded	1	1.8%
Total	56	100.0%

Correlated Data Summary

Number of Levels	Subject Effect	patients	31
	Within-Subject Effect	eyes	2
Number of Subjects			31
Number of Measurements per Subject	Minimum		1
	Maximum		2
Correlation Matrix Dimension			2

Categorical Variable Information

			N	Percent
Factor	group	1	28	50.9%
		2	27	49.1%
		Total	55	100.0%
	eyes	1	31	56.4%
		2	24	43.6%
		Total	55	100.0%

Continuous Variable Information

		N	Minimum	Maximum	Mean	Std. Deviation
Dependent Variable	Trefoil3	55	.00	.10	.0341	.02074

Goodness of Fit[a]

	Value
Quasi Likelihood under Independence Model Criterion (QIC)[b]	8.150
Corrected Quasi Likelihood under Independence Model Criterion (QICC)[b]	8.023

Dependent Variable: Trefoil3
Model: (Intercept), group, eyes, group * eyes[a]

a. Information criteria are in smaller-is-better form.

b. Computed using the full log quasi-likelihood function.

Tests of Model Effects

Source	Type III		
	Wald Chi-Square	df	Sig.
(Intercept)	118.759	1	.000
group	.606	1	.436
eyes	.051	1	.821
group * eyes	.216	1	.642

Dependent Variable: Trefoil3
Model: (Intercept), group, eyes, group * eyes

Parameter Estimates

Parameter	B	Std. Error	95% Wald Confidence Interval		Hypothesis ...
			Lower	Upper	Wald Chi-Square
(Intercept)	.031	.0061	.019	.043	25.953
[group=1]	.007	.0092	-.011	.025	.611
[group=2]	0[a]	.	.	.	.
[eyes=1]	.001	.0060	-.011	.013	.038
[eyes=2]	0[a]	.	.	.	.
[group=1] * [eyes=1]	-.005	.0098	-.024	.015	.216
[group=1] * [eyes=2]	0[a]	.	.	.	.
[group=2] * [eyes=1]	0[a]	.	.	.	.
[group=2] * [eyes=2]	0[a]	.	.	.	.
(Scale)	.000				

Parameter Estimates

Parameter	Hypothesis Test	
	df	Sig.
(Intercept)	1	.000
[group=1]	1	.435
[group=2]	.	.
[eyes=1]	1	.846
[eyes=2]	.	.
[group=1] * [eyes=1]	1	.642
[group=1] * [eyes=2]	.	.
[group=2] * [eyes=1]	.	.
[group=2] * [eyes=2]	.	.
(Scale)		

Dependent Variable: Trefoil3
Model: (Intercept), group, eyes, group * eyes

a. Set to zero because this parameter is redundant.

```
 * Generalized Estimating Equations.
GENLIN tHOA1W3 BY group eyes (ORDER=ASCENDING)
  /MODEL group eyes group*eyes INTERCEPT=YES
 DISTRIBUTION=NORMAL LINK=IDENTITY
  /CRITERIA SCALE=MLE PCONVERGE=1E-006(ABSOLUTE) SINGULAR=1E-012 ANALYSISTY
PE=3(WALD) CILEVEL=95
    LIKELIHOOD=FULL
  /REPEATED SUBJECT=patients WITHINSUBJECT=eyes SORT=YES CORRTYPE=INDEPENDE
NT ADJUSTCORR=YES
    COVB=ROBUST
  /MISSING CLASSMISSING=EXCLUDE
  /PRINT CPS DESCRIPTIVES MODELINFO FIT SUMMARY SOLUTION.
```

Generalized Linear Models

Notes

Output Created		29-NOV-2023 19:51...
Comments		
Input	Data	/Users/yangshan/Desktop/2022-5-12//2023-10-OBL/2023-11-22 revised manuscript/OBL.sav
	Active Dataset	DataSet1
	Filter	<none>
	Weight	<none>
	Split File	<none>
	N of Rows in Working Data File	56
Missing Value Handling	Definition of Missing	User-defined missing values for factor, subject and within-subject variables are treated as missing.
	Cases Used	Statistics are based on cases with valid data for all variables in the model.
Weight Handling		not applicable

Notes

Syntax		GENLIN tHOA1W3 BY group eyes (ORDER=ASCENDING)<br>/MODEL group eyes group*eyes INTERCEPT=YES<br><br>DISTRIBUTION=NORMAL LINK=IDENTITY<br>/CRITERIA SCALE=MLE PCONVERGE=1E-006 (ABSOLUTE) SINGULAR=1E-012 ANALYSISTYPE=3(WALD) CILEVEL=95<br>LIKELIHOOD=FULL<br>/REPEATED SUBJECT=patients WITHINSUBJECT=eyes SORT=YES CORRTYPE=INDEPENDENT ADJUSTCORR=YES<br>COVB=ROBUST<br>/MISSING CLASSMISSING=EXCLUDE<br>/PRINT CPS DESCRIPTIVES MODELINFO FIT SUMMARY SOLUTION.
Resources	Processor Time	00:00:00.05
	Elapsed Time	00:00:01.00

Model Information

Dependent Variable		tHOA1W3
Probability Distribution		Normal
Link Function		Identity
Subject Effect	1	patients
Within-Subject Effect	1	eyes
Working Correlation Matrix Structure		Independent

Case Processing Summary

	N	Percent
Included	53	94.6%
Excluded	3	5.4%
Total	56	100.0%

Correlated Data Summary

Number of Levels	Subject Effect	patients	30
	Within-Subject Effect	eyes	2
Number of Subjects			30
Number of Measurements per Subject	Minimum		1
	Maximum		2
Correlation Matrix Dimension			2

Categorical Variable Information

			N	Percent
Factor	group	1	26	49.1%
		2	27	50.9%
		Total	53	100.0%
	eyes	1	30	56.6%
		2	23	43.4%
		Total	53	100.0%

Continuous Variable Information

		N	Minimum	Maximum	Mean	Std. Deviation
Dependent Variable	tHOA1W3	53	.02	.22	.0956	.04965

Goodness of Fit[a]

	Value
Quasi Likelihood under Independence Model Criterion (QIC)[b]	7.880
Corrected Quasi Likelihood under Independence Model Criterion (QICC)[b]	8.126

Dependent Variable: tHOA1W3
Model: (Intercept), group, eyes, group * eyes[a]

a. Information criteria are in smaller-is-better form.

b. Computed using the full log quasi-likelihood function.

Tests of Model Effects

Source	Type III		
	Wald Chi-Square	df	Sig.
(Intercept)	139.110	1	.000
group	.108	1	.743
eyes	.562	1	.454
group * eyes	.581	1	.446

Dependent Variable: tHOA1W3
Model: (Intercept), group, eyes, group * eyes

Parameter Estimates

Parameter	B	Std. Error	95% Wald Confidence Interval		Hypothesis ...
			Lower	Upper	Wald Chi-Square
(Intercept)	.093	.0123	.068	.117	56.390
[group=1]	-.002	.0175	-.036	.033	.009
[group=2]	0[a]	.	.	.	.
[eyes=1]	.000	.0143	-.028	.028	.000
[eyes=2]	0[a]	.	.	.	.
[group=1] * [eyes=1]	.014	.0183	-.022	.050	.581
[group=1] * [eyes=2]	0[a]	.	.	.	.
[group=2] * [eyes=1]	0[a]	.	.	.	.
[group=2] * [eyes=2]	0[a]	.	.	.	.
(Scale)	.003				

Parameter Estimates

Parameter	Hypothesis Test	
	df	Sig.
(Intercept)	1	.000
[group=1]	1	.924
[group=2]	.	.
[eyes=1]	1	.993
[eyes=2]	.	.
[group=1] * [eyes=1]	1	.446
[group=1] * [eyes=2]	.	.
[group=2] * [eyes=1]	.	.
[group=2] * [eyes=2]	.	.
(Scale)		

Dependent Variable: tHOA1W3
Model: (Intercept), group, eyes, group * eyes

a. Set to zero because this parameter is redundant.

```
* Generalized Estimating Equations.
GENLIN Coma1w3 BY group eyes (ORDER=ASCENDING)
  /MODEL group eyes group*eyes INTERCEPT=YES
 DISTRIBUTION=NORMAL LINK=IDENTITY
  /CRITERIA SCALE=MLE PCONVERGE=1E-006(ABSOLUTE) SINGULAR=1E-012 ANALYSISTYPE=3(WALD) CILEVEL=95
    LIKELIHOOD=FULL
  /REPEATED SUBJECT=patients WITHINSUBJECT=eyes SORT=YES CORRTYPE=INDEPENDENT ADJUSTCORR=YES
    COVB=ROBUST
  /MISSING CLASSMISSING=EXCLUDE
  /PRINT CPS DESCRIPTIVES MODELINFO FIT SUMMARY SOLUTION.
```

Generalized Linear Models

Notes

Output Created		29-NOV-2023 19:52...
Comments		
Input	Data	/Users/yangshan/Desktop/2022-5-12//2023-10-OBL/2023-11-22 revised manuscript/OBL.sav
	Active Dataset	DataSet1
	Filter	<none>
	Weight	<none>
	Split File	<none>
	N of Rows in Working Data File	56
Missing Value Handling	Definition of Missing	User-defined missing values for factor, subject and within-subject variables are treated as missing.
	Cases Used	Statistics are based on cases with valid data for all variables in the model.
Weight Handling		not applicable

Notes

Syntax		GENLIN Coma1w3 BY group eyes (ORDER=ASCENDING)<br>/MODEL group eyes group*eyes INTERCEPT=YES<br><br>DISTRIBUTION=NORMAL LINK=IDENTITY<br>/CRITERIA SCALE=MLE PCONVERGE=1E-006 (ABSOLUTE) SINGULAR=1E-012 ANALYSISTYPE=3(WALD) CILEVEL=95<br>LIKELIHOOD=FULL<br>/REPEATED SUBJECT=patients WITHINSUBJECT=eyes SORT=YES CORRTYPE=INDEPENDENT ADJUSTCORR=YES<br>COVB=ROBUST<br>/MISSING CLASSMISSING=EXCLUDE<br>/PRINT CPS DESCRIPTIVES MODELINFO FIT SUMMARY SOLUTION.
Resources	Processor Time	00:00:00.05
	Elapsed Time	00:00:01.00

Model Information

Dependent Variable		Coma1w3
Probability Distribution		Normal
Link Function		Identity
Subject Effect	1	patients
Within-Subject Effect	1	eyes
Working Correlation Matrix Structure		Independent

Case Processing Summary

	N	Percent
Included	53	94.6%
Excluded	3	5.4%
Total	56	100.0%

Correlated Data Summary

Number of Levels	Subject Effect	patients	30
	Within-Subject Effect	eyes	2
Number of Subjects			30
Number of Measurements per Subject	Minimum		1
	Maximum		2
Correlation Matrix Dimension			2

Categorical Variable Information

			N	Percent
Factor	group	1	26	49.1%
		2	27	50.9%
		Total	53	100.0%
	eyes	1	30	56.6%
		2	23	43.4%
		Total	53	100.0%

Continuous Variable Information

		N	Minimum	Maximum	Mean	Std. Deviation
Dependent Variable	Coma1w3	53	.01	.22	.0717	.05369

Goodness of Fit[a]

	Value
Quasi Likelihood under Independence Model Criterion (QIC)[b]	7.896
Corrected Quasi Likelihood under Independence Model Criterion (QICC)[b]	8.144

Dependent Variable: Coma1w3
Model: (Intercept), group, eyes, group * eyes[a]

a. Information criteria are in smaller-is-better form.

b. Computed using the full log quasi-likelihood function.

Tests of Model Effects

Source	Type III Wald Chi-Square	df	Sig.
(Intercept)	76.630	1	.000
group	.362	1	.547
eyes	1.321	1	.250
group * eyes	1.118	1	.290

Dependent Variable: Coma1w3
Model: (Intercept), group, eyes, group * eyes

Parameter Estimates

Parameter	B	Std. Error	95% Wald Confidence Interval Lower	95% Wald Confidence Interval Upper	Hypothesis ... Wald Chi-Square
(Intercept)	.065	.0134	.039	.092	23.899
[group=1]	-.002	.0187	-.039	.034	.015
[group=2]	0[a]	.	.	.	.
[eyes=1]	.001	.0161	-.030	.033	.004
[eyes=2]	0[a]	.	.	.	.
[group=1] * [eyes=1]	.024	.0228	-.021	.069	1.118
[group=1] * [eyes=2]	0[a]	.	.	.	.
[group=2] * [eyes=1]	0[a]	.	.	.	.
[group=2] * [eyes=2]	0[a]	.	.	.	.
(Scale)	.003				

Parameter Estimates

Parameter	Hypothesis Test df	Sig.
(Intercept)	1	.000
[group=1]	1	.901
[group=2]	.	.
[eyes=1]	1	.948
[eyes=2]	.	.
[group=1] * [eyes=1]	1	.290
[group=1] * [eyes=2]	.	.
[group=2] * [eyes=1]	.	.
[group=2] * [eyes=2]	.	.
(Scale)		

Dependent Variable: Coma1w3
Model: (Intercept), group, eyes, group * eyes

a. Set to zero because this parameter is redundant.

```
* Generalized Estimating Equations.
GENLIN SA1w3 BY group eyes (ORDER=ASCENDING)
  /MODEL group eyes group*eyes INTERCEPT=YES
 DISTRIBUTION=NORMAL LINK=IDENTITY
  /CRITERIA SCALE=MLE PCONVERGE=1E-006(ABSOLUTE) SINGULAR=1E-012 ANALYSISTYPE=3(WALD) CILEVEL=95
    LIKELIHOOD=FULL
  /REPEATED SUBJECT=patients WITHINSUBJECT=eyes SORT=YES CORRTYPE=INDEPENDENT ADJUSTCORR=YES
    COVB=ROBUST
  /MISSING CLASSMISSING=EXCLUDE
  /PRINT CPS DESCRIPTIVES MODELINFO FIT SUMMARY SOLUTION.
```

Generalized Linear Models

Notes

Output Created		29-NOV-2023 19:52...
Comments		
Input	Data	/Users/yangshan/Desktop/2022-5-12/ /2023-10-OBL/2023-11-22 revised manuscript/OBL.sav
	Active Dataset	DataSet1
	Filter	<none>
	Weight	<none>
	Split File	<none>
	N of Rows in Working Data File	56
Missing Value Handling	Definition of Missing	User-defined missing values for factor, subject and within-subject variables are treated as missing.
	Cases Used	Statistics are based on cases with valid data for all variables in the model.
Weight Handling		not applicable

Notes

Syntax		GENLIN SA1w3 BY group eyes (ORDER=ASCENDING)<br>/MODEL group eyes group*eyes INTERCEPT=YES<br><br>DISTRIBUTION=NORMAL LINK=IDENTITY<br>/CRITERIA SCALE=MLE PCONVERGE=1E-006 (ABSOLUTE) SINGULAR=1E-012 ANALYSISTYPE=3(WALD) CILEVEL=95<br>LIKELIHOOD=FULL<br>/REPEATED SUBJECT=patients WITHINSUBJECT=eyes SORT=YES CORRTYPE=INDEPENDENT ADJUSTCORR=YES<br>COVB=ROBUST<br>/MISSING CLASSMISSING=EXCLUDE<br>/PRINT CPS DESCRIPTIVES MODELINFO FIT SUMMARY SOLUTION.
Resources	Processor Time	00:00:00.04
	Elapsed Time	00:00:00.00

Model Information

Dependent Variable		SA1w3
Probability Distribution		Normal
Link Function		Identity
Subject Effect	1	patients
Within-Subject Effect	1	eyes
Working Correlation Matrix Structure		Independent

Case Processing Summary

	N	Percent
Included	53	94.6%
Excluded	3	5.4%
Total	56	100.0%

Correlated Data Summary

Number of Levels	Subject Effect	patients	30
	Within-Subject Effect	eyes	2
Number of Subjects			30
Number of Measurements per Subject	Minimum		1
	Maximum		2
Correlation Matrix Dimension			2

Categorical Variable Information

			N	Percent
Factor	group	1	26	49.1%
		2	27	50.9%
		Total	53	100.0%
	eyes	1	30	56.6%
		2	23	43.4%
		Total	53	100.0%

Continuous Variable Information

		N	Minimum	Maximum	Mean	Std. Deviation
Dependent Variable	SA1w3	53	-.09	.05	-.0056	.03384

Goodness of Fit[a]

	Value
Quasi Likelihood under Independence Model Criterion (QIC)[b]	7.803
Corrected Quasi Likelihood under Independence Model Criterion (QICC)[b]	8.056

Dependent Variable: SA1w3
Model: (Intercept), group, eyes, group * eyes[a]

a. Information criteria are in smaller-is-better form.

b. Computed using the full log quasi-likelihood function.

Tests of Model Effects

Source	Type III		
	Wald Chi-Square	df	Sig.
(Intercept)	1.628	1	.202
group	.002	1	.969
eyes	2.424	1	.119
group * eyes	2.337	1	.126

Dependent Variable: SA1w3
Model: (Intercept), group, eyes, group * eyes

Parameter Estimates

Parameter	B	Std. Error	95% Wald Confidence Interval		Hypothesis ...
			Lower	Upper	Wald Chi-Square
(Intercept)	-.018	.0100	-.037	.002	3.088
[group=1]	.011	.0119	-.012	.034	.866
[group=2]	0[a]	.	.	.	.
[eyes=1]	.023	.0131	-.003	.049	3.118
[eyes=2]	0[a]	.	.	.	.
[group=1] * [eyes=1]	-.023	.0149	-.052	.006	2.337
[group=1] * [eyes=2]	0[a]	.	.	.	.
[group=2] * [eyes=1]	0[a]	.	.	.	.
[group=2] * [eyes=2]	0[a]	.	.	.	.
(Scale)	.001				

Parameter Estimates

Parameter	Hypothesis Test	
	df	Sig.
(Intercept)	1	.079
[group=1]	1	.352
[group=2]	.	.
[eyes=1]	1	.077
[eyes=2]	.	.
[group=1] * [eyes=1]	1	.126
[group=1] * [eyes=2]	.	.
[group=2] * [eyes=1]	.	.
[group=2] * [eyes=2]	.	.
(Scale)		

Dependent Variable: SA1w3
Model: (Intercept), group, eyes, group * eyes

a. Set to zero because this parameter is redundant.

```
 * Generalized Estimating Equations.
GENLIN Trefoil1w3 BY group eyes (ORDER=ASCENDING)
  /MODEL group eyes group*eyes INTERCEPT=YES
 DISTRIBUTION=NORMAL LINK=IDENTITY
  /CRITERIA SCALE=MLE PCONVERGE=1E-006(ABSOLUTE) SINGULAR=1E-012 ANALYSISTYPE=3(WALD) CILEVEL=95
    LIKELIHOOD=FULL
  /REPEATED SUBJECT=patients WITHINSUBJECT=eyes SORT=YES CORRTYPE=INDEPENDENT ADJUSTCORR=YES
    COVB=ROBUST
  /MISSING CLASSMISSING=EXCLUDE
  /PRINT CPS DESCRIPTIVES MODELINFO FIT SUMMARY SOLUTION.
```

Generalized Linear Models

Notes

Output Created		29-NOV-2023 19:52...
Comments		
Input	Data	/Users/yangshan/Desktop/2022-5-12//2023-10-OBL/2023-11-22 revised manuscript/OBL.sav
	Active Dataset	DataSet1
	Filter	<none>
	Weight	<none>
	Split File	<none>
	N of Rows in Working Data File	56
Missing Value Handling	Definition of Missing	User-defined missing values for factor, subject and within-subject variables are treated as missing.
	Cases Used	Statistics are based on cases with valid data for all variables in the model.
Weight Handling		not applicable

Notes

Syntax		GENLIN Trefoil1w3 BY group eyes (ORDER=ASCENDING)<br>/MODEL group eyes group*eyes INTERCEPT=YES<br><br>DISTRIBUTION=NORMAL LINK=IDENTITY<br>/CRITERIA SCALE=MLE PCONVERGE=1E-006 (ABSOLUTE) SINGULAR=1E-012 ANALYSISTYPE=3(WALD) CILEVEL=95<br>LIKELIHOOD=FULL<br>/REPEATED SUBJECT=patients WITHINSUBJECT=eyes SORT=YES CORRTYPE=INDEPENDENT ADJUSTCORR=YES<br>COVB=ROBUST<br>/MISSING CLASSMISSING=EXCLUDE<br>/PRINT CPS DESCRIPTIVES MODELINFO FIT SUMMARY SOLUTION.
Resources	Processor Time	00:00:00.04
	Elapsed Time	00:00:00.00

Model Information

Dependent Variable		Trefoil1w3
Probability Distribution		Normal
Link Function		Identity
Subject Effect	1	patients
Within-Subject Effect	1	eyes
Working Correlation Matrix Structure		Independent

Case Processing Summary

	N	Percent
Included	55	98.2%
Excluded	1	1.8%
Total	56	100.0%

Correlated Data Summary

Number of Levels	Subject Effect	patients	31
	Within-Subject Effect	eyes	2
Number of Subjects			31
Number of Measurements per Subject	Minimum		1
	Maximum		2
Correlation Matrix Dimension			2

Categorical Variable Information

			N	Percent
Factor	group	1	28	50.9%
		2	27	49.1%
		Total	55	100.0%
	eyes	1	31	56.4%
		2	24	43.6%
		Total	55	100.0%

Continuous Variable Information

		N	Minimum	Maximum	Mean	Std. Deviation
Dependent Variable	Trefoil1w3	55	-.04	.04	.0055	.01671

Goodness of Fit[a]

	Value
Quasi Likelihood under Independence Model Criterion (QIC)[b]	8.054
Corrected Quasi Likelihood under Independence Model Criterion (QICC)[b]	8.012

Dependent Variable: Trefoil1w3
Model: (Intercept), group, eyes, group * eyes[a]

a. Information criteria are in smaller-is-better form.

b. Computed using the full log quasi-likelihood function.

Tests of Model Effects

Source	Type III		
	Wald Chi-Square	df	Sig.
(Intercept)	8.995	1	.003
group	10.207	1	.001
eyes	2.511	1	.113
group * eyes	2.692	1	.101

Dependent Variable: Trefoil1w3
Model: (Intercept), group, eyes, group * eyes

Parameter Estimates

Parameter	B	Std. Error	95% Wald Confidence Interval		Hypothesis ...
			Lower	Upper	Wald Chi-Square
(Intercept)	-.001	.0047	-.010	.009	.011
[group=1]	.019	.0061	.007	.031	10.067
[group=2]	0[a]	.	.	.	.
[eyes=1]	.000	.0061	-.012	.012	.001
[eyes=2]	0[a]	.	.	.	.
[group=1] * [eyes=1]	-.014	.0083	-.030	.003	2.692
[group=1] * [eyes=2]	0[a]	.	.	.	.
[group=2] * [eyes=1]	0[a]	.	.	.	.
[group=2] * [eyes=2]	0[a]	.	.	.	.
(Scale)	.000				

Parameter Estimates

Parameter	Hypothesis Test	
	df	Sig.
(Intercept)	1	.915
[group=1]	1	.002
[group=2]	.	.
[eyes=1]	1	.970
[eyes=2]	.	.
[group=1] * [eyes=1]	1	.101
[group=1] * [eyes=2]	.	.
[group=2] * [eyes=1]	.	.
[group=2] * [eyes=2]	.	.
(Scale)		

Dependent Variable: Trefoil1w3
Model: (Intercept), group, eyes, group * eyes

a. Set to zero because this parameter is redundant.

```
* Generalized Estimating Equations.
GENLIN tHOAs1m3 BY group eyes (ORDER=ASCENDING)
  /MODEL group eyes group*eyes INTERCEPT=YES
 DISTRIBUTION=NORMAL LINK=IDENTITY
  /CRITERIA SCALE=MLE PCONVERGE=1E-006(ABSOLUTE) SINGULAR=1E-012 ANALYSISTYPE=3(WALD) CILEVEL=95
    LIKELIHOOD=FULL
  /REPEATED SUBJECT=patients WITHINSUBJECT=eyes SORT=YES CORRTYPE=INDEPENDENT ADJUSTCORR=YES
    COVB=ROBUST
  /MISSING CLASSMISSING=EXCLUDE
  /PRINT CPS DESCRIPTIVES MODELINFO FIT SUMMARY SOLUTION.
```

Generalized Linear Models

Notes

Output Created		29-NOV-2023 19:52...
Comments		
Input	Data	/Users/yangshan/Desktop/2022-5-12/ /2023-10-OBL/2023-11-22 revised manuscript/OBL .sav
	Active Dataset	DataSet1
	Filter	<none>
	Weight	<none>
	Split File	<none>
	N of Rows in Working Data File	56
Missing Value Handling	Definition of Missing	User-defined missing values for factor, subject and within-subject variables are treated as missing.
	Cases Used	Statistics are based on cases with valid data for all variables in the model.
Weight Handling		not applicable

Notes

Syntax		GENLIN tHOAs1m3 BY group eyes (ORDER=ASCENDING)<br>/MODEL group eyes group*eyes INTERCEPT=YES<br><br>DISTRIBUTION=NORMAL LINK=IDENTITY<br>/CRITERIA SCALE=MLE PCONVERGE=1E-006 (ABSOLUTE) SINGULAR=1E-012 ANALYSISTYPE=3(WALD) CILEVEL=95<br>LIKELIHOOD=FULL<br>/REPEATED SUBJECT=patients WITHINSUBJECT=eyes SORT=YES CORRTYPE=INDEPENDENT ADJUSTCORR=YES<br>COVB=ROBUST<br>/MISSING CLASSMISSING=EXCLUDE<br>/PRINT CPS DESCRIPTIVES MODELINFO FIT SUMMARY SOLUTION.
Resources	Processor Time	00:00:00.04
	Elapsed Time	00:00:00.00

Model Information

Dependent Variable		tHOAs1m3
Probability Distribution		Normal
Link Function		Identity
Subject Effect	1	patients
Within-Subject Effect	1	eyes
Working Correlation Matrix Structure		Independent

Case Processing Summary

	N	Percent
Included	54	96.4%
Excluded	2	3.6%
Total	56	100.0%

Correlated Data Summary

Number of Levels	Subject Effect	patients	31
	Within-Subject Effect	eyes	2
Number of Subjects			31
Number of Measurements per Subject	Minimum		1
	Maximum		2
Correlation Matrix Dimension			2

Categorical Variable Information

			N	Percent
Factor	group	1	27	50.0%
		2	27	50.0%
		Total	54	100.0%
	eyes	1	31	57.4%
		2	23	42.6%
		Total	54	100.0%

Continuous Variable Information

		N	Minimum	Maximum	Mean	Std. Deviation
Dependent Variable	tHOAs1m3	54	.03	.34	.1060	.05921

Goodness of Fit[a]

	Value
Quasi Likelihood under Independence Model Criterion (QIC)[b]	8.576
Corrected Quasi Likelihood under Independence Model Criterion (QICC)[b]	8.175

Dependent Variable: tHOAs1m3
Model: (Intercept), group, eyes, group * eyes[a]

a. Information criteria are in smaller-is-better form.

b. Computed using the full log quasi-likelihood function.

Tests of Model Effects

Source	Type III		
	Wald Chi-Square	df	Sig.
(Intercept)	104.776	1	.000
group	1.701	1	.192
eyes	.100	1	.752
group * eyes	.403	1	.526

Dependent Variable: tHOAs1m3
Model: (Intercept), group, eyes, group * eyes

Parameter Estimates

Parameter	B	Std. Error	95% Wald Confidence Interval		Hypothesis ...
			Lower	Upper	Wald Chi-Square
(Intercept)	.098	.0131	.072	.123	55.872
[group=1]	.020	.0277	-.034	.075	.540
[group=2]	0[a]	.	.	.	.
[eyes=1]	-.010	.0100	-.030	.009	1.023
[eyes=2]	0[a]	.	.	.	.
[group=1] * [eyes=1]	.013	.0212	-.028	.055	.403
[group=1] * [eyes=2]	0[a]	.	.	.	.
[group=2] * [eyes=1]	0[a]	.	.	.	.
[group=2] * [eyes=2]	0[a]	.	.	.	.
(Scale)	.003				

Parameter Estimates

Parameter	Hypothesis Test	
	df	Sig.
(Intercept)	1	.000
[group=1]	1	.463
[group=2]	.	.
[eyes=1]	1	.312
[eyes=2]	.	.
[group=1] * [eyes=1]	1	.526
[group=1] * [eyes=2]	.	.
[group=2] * [eyes=1]	.	.
[group=2] * [eyes=2]	.	.
(Scale)		

Dependent Variable: tHOAs1m3
Model: (Intercept), group, eyes, group * eyes

a. Set to zero because this parameter is redundant.

```
 * Generalized Estimating Equations.
GENLIN Coma1m3 BY group eyes (ORDER=ASCENDING)
  /MODEL group eyes group*eyes INTERCEPT=YES
 DISTRIBUTION=NORMAL LINK=IDENTITY
  /CRITERIA SCALE=MLE PCONVERGE=1E-006(ABSOLUTE) SINGULAR=1E-012 ANALYSISTYPE=3(WALD) CILEVEL=95
    LIKELIHOOD=FULL
  /REPEATED SUBJECT=patients WITHINSUBJECT=eyes SORT=YES CORRTYPE=INDEPENDENT ADJUSTCORR=YES
    COVB=ROBUST
  /MISSING CLASSMISSING=EXCLUDE
  /PRINT CPS DESCRIPTIVES MODELINFO FIT SUMMARY SOLUTION.
```

Generalized Linear Models

Notes

Output Created		29-NOV-2023 19:53...
Comments		
Input	Data	/Users/yangshan/Desktop/2022-5-12/ /2023-10-OBL/2023-11-22 revised manuscript/OBL .sav
	Active Dataset	DataSet1
	Filter	<none>
	Weight	<none>
	Split File	<none>
	N of Rows in Working Data File	56
Missing Value Handling	Definition of Missing	User-defined missing values for factor, subject and within-subject variables are treated as missing.
	Cases Used	Statistics are based on cases with valid data for all variables in the model.
Weight Handling		not applicable

Notes

Syntax		GENLIN Coma1m3 BY group eyes (ORDER=ASCENDING)<br>/MODEL group eyes group*eyes INTERCEPT=YES<br><br>DISTRIBUTION=NORMAL LINK=IDENTITY<br>/CRITERIA SCALE=MLE PCONVERGE=1E-006 (ABSOLUTE) SINGULAR=1E-012 ANALYSISTYPE=3(WALD) CILEVEL=95<br>LIKELIHOOD=FULL<br>/REPEATED SUBJECT=patients WITHINSUBJECT=eyes SORT=YES CORRTYPE=INDEPENDENT ADJUSTCORR=YES<br>COVB=ROBUST<br>/MISSING CLASSMISSING=EXCLUDE<br>/PRINT CPS DESCRIPTIVES MODELINFO FIT SUMMARY SOLUTION.
Resources	Processor Time	00:00:00.04
	Elapsed Time	00:00:00.00

Model Information

Dependent Variable		Coma1m3
Probability Distribution		Normal
Link Function		Identity
Subject Effect	1	patients
Within-Subject Effect	1	eyes
Working Correlation Matrix Structure		Independent

Case Processing Summary

	N	Percent
Included	54	96.4%
Excluded	2	3.6%
Total	56	100.0%

Correlated Data Summary

Number of Levels	Subject Effect	patients	31
	Within-Subject Effect	eyes	2
Number of Subjects			31
Number of Measurements per Subject	Minimum		1
	Maximum		2
Correlation Matrix Dimension			2

Categorical Variable Information

			N	Percent
Factor	group	1	27	50.0%
		2	27	50.0%
		Total	54	100.0%
	eyes	1	31	57.4%
		2	23	42.6%
		Total	54	100.0%

Continuous Variable Information

		N	Minimum	Maximum	Mean	Std. Deviation
Dependent Variable	Coma1m3	54	.01	.17	.0688	.04358

Goodness of Fit[a]

	Value
Quasi Likelihood under Independence Model Criterion (QIC)[b]	7.887
Corrected Quasi Likelihood under Independence Model Criterion (QICC)[b]	8.092

Dependent Variable: Coma1m3
Model: (Intercept), group, eyes, group * eyes[a]

a. Information criteria are in smaller-is-better form.

b. Computed using the full log quasi-likelihood function.

Tests of Model Effects

Source	Type III		
	Wald Chi-Square	df	Sig.
(Intercept)	109.236	1	.000
group	.028	1	.866
eyes	1.008	1	.315
group * eyes	6.886	1	.009

Dependent Variable: Coma1m3
Model: (Intercept), group, eyes, group * eyes

Parameter Estimates

Parameter	B	Std. Error	95% Wald Confidence Interval		Hypothesis ...
			Lower	Upper	Wald Chi-Square
(Intercept)	.074	.0143	.046	.102	26.444
[group=1]	-.021	.0160	-.052	.011	1.690
[group=2]	0[a]	.	.	.	.
[eyes=1]	-.014	.0127	-.039	.011	1.248
[eyes=2]	0[a]	.	.	.	.
[group=1] * [eyes=1]	.046	.0176	.012	.080	6.886
[group=1] * [eyes=2]	0[a]	.	.	.	.
[group=2] * [eyes=1]	0[a]	.	.	.	.
[group=2] * [eyes=2]	0[a]	.	.	.	.
(Scale)	.002				

Parameter Estimates

Parameter	Hypothesis Test	
	df	Sig.
(Intercept)	1	.000
[group=1]	1	.194
[group=2]	.	.
[eyes=1]	1	.264
[eyes=2]	.	.
[group=1] * [eyes=1]	1	.009
[group=1] * [eyes=2]	.	.
[group=2] * [eyes=1]	.	.
[group=2] * [eyes=2]	.	.
(Scale)		

Dependent Variable: Coma1m3
Model: (Intercept), group, eyes, group * eyes

a. Set to zero because this parameter is redundant.

```
* Generalized Estimating Equations.
GENLIN SA1m3 BY group eyes (ORDER=ASCENDING)
  /MODEL group eyes group*eyes INTERCEPT=YES
 DISTRIBUTION=NORMAL LINK=IDENTITY
  /CRITERIA SCALE=MLE PCONVERGE=1E-006(ABSOLUTE) SINGULAR=1E-012 ANALYSISTY
PE=3(WALD) CILEVEL=95
    LIKELIHOOD=FULL
  /REPEATED SUBJECT=patients WITHINSUBJECT=eyes SORT=YES CORRTYPE=INDEPENDE
NT ADJUSTCORR=YES
    COVB=ROBUST
  /MISSING CLASSMISSING=EXCLUDE
  /PRINT CPS DESCRIPTIVES MODELINFO FIT SUMMARY SOLUTION.
```

Generalized Linear Models

Notes

Output Created		29-NOV-2023 19:53...
Comments		
Input	Data	/Users/yangshan/Desktop/2022-5-12/ /2023-10-OBL/2023-11-22 revised manuscript/OBL .sav
	Active Dataset	DataSet1
	Filter	<none>
	Weight	<none>
	Split File	<none>
	N of Rows in Working Data File	56
Missing Value Handling	Definition of Missing	User-defined missing values for factor, subject and within-subject variables are treated as missing.
	Cases Used	Statistics are based on cases with valid data for all variables in the model.
Weight Handling		not applicable

Notes

Syntax		GENLIN SA1m3 BY group eyes (ORDER=ASCENDING)<br>/MODEL group eyes group*eyes INTERCEPT=YES<br><br>DISTRIBUTION=NORMAL LINK=IDENTITY<br>/CRITERIA SCALE=MLE PCONVERGE=1E-006 (ABSOLUTE) SINGULAR=1E-012 ANALYSISTYPE=3(WALD) CILEVEL=95<br>LIKELIHOOD=FULL<br>/REPEATED SUBJECT=patients WITHINSUBJECT=eyes SORT=YES CORRTYPE=INDEPENDENT ADJUSTCORR=YES<br>COVB=ROBUST<br>/MISSING CLASSMISSING=EXCLUDE<br>/PRINT CPS DESCRIPTIVES MODELINFO FIT SUMMARY SOLUTION.
Resources	Processor Time	00:00:00.04
	Elapsed Time	00:00:00.00

Model Information

Dependent Variable		SA1m3
Probability Distribution		Normal
Link Function		Identity
Subject Effect	1	patients
Within-Subject Effect	1	eyes
Working Correlation Matrix Structure		Independent

Case Processing Summary

	N	Percent
Included	54	96.4%
Excluded	2	3.6%
Total	56	100.0%

Correlated Data Summary

Number of Levels	Subject Effect	patients	31
	Within-Subject Effect	eyes	2
Number of Subjects			31
Number of Measurements per Subject	Minimum		1
	Maximum		2
Correlation Matrix Dimension			2

Categorical Variable Information

			N	Percent
Factor	group	1	27	50.0%
		2	27	50.0%
		Total	54	100.0%
	eyes	1	31	57.4%
		2	23	42.6%
		Total	54	100.0%

Continuous Variable Information

		N	Minimum	Maximum	Mean	Std. Deviation
Dependent Variable	SA1m3	54	-.08	.07	.0027	.03117

Goodness of Fit[a]

	Value
Quasi Likelihood under Independence Model Criterion (QIC)[b]	7.783
Corrected Quasi Likelihood under Independence Model Criterion (QICC)[b]	8.048

Dependent Variable: SA1m3
Model: (Intercept), group, eyes, group * eyes[a]

a. Information criteria are in smaller-is-better form.

b. Computed using the full log quasi-likelihood function.

Tests of Model Effects

Source	Type III		
	Wald Chi-Square	df	Sig.
(Intercept)	.333	1	.564
group	1.336	1	.248
eyes	.328	1	.567
group * eyes	2.473	1	.116

Dependent Variable: SA1m3
Model: (Intercept), group, eyes, group * eyes

Parameter Estimates

Parameter	B	Std. Error	95% Wald Confidence Interval		Hypothesis ...
			Lower	Upper	Wald Chi-Square
(Intercept)	.001	.0090	-.017	.019	.015
[group=1]	-.001	.0112	-.023	.021	.002
[group=2]	0[a]	.	.	.	.
[eyes=1]	.014	.0072	.000	.028	3.665
[eyes=2]	0[a]	.	.	.	.
[group=1] * [eyes=1]	-.020	.0128	-.045	.005	2.473
[group=1] * [eyes=2]	0[a]	.	.	.	.
[group=2] * [eyes=1]	0[a]	.	.	.	.
[group=2] * [eyes=2]	0[a]	.	.	.	.
(Scale)	.001				

Parameter Estimates

Parameter	Hypothesis Test	
	df	Sig.
(Intercept)	1	.904
[group=1]	1	.962
[group=2]	.	.
[eyes=1]	1	.056
[eyes=2]	.	.
[group=1] * [eyes=1]	1	.116
[group=1] * [eyes=2]	.	.
[group=2] * [eyes=1]	.	.
[group=2] * [eyes=2]	.	.
(Scale)		

Dependent Variable: SA1m3
Model: (Intercept), group, eyes, group * eyes

a. Set to zero because this parameter is redundant.

```
* Generalized Estimating Equations.
GENLIN Trefoil1m3 BY group eyes (ORDER=ASCENDING)
  /MODEL group eyes group*eyes INTERCEPT=YES
 DISTRIBUTION=NORMAL LINK=IDENTITY
  /CRITERIA SCALE=MLE PCONVERGE=1E-006(ABSOLUTE) SINGULAR=1E-012 ANALYSISTYPE=3(WALD) CILEVEL=95
    LIKELIHOOD=FULL
  /REPEATED SUBJECT=patients WITHINSUBJECT=eyes SORT=YES CORRTYPE=INDEPENDENT ADJUSTCORR=YES
    COVB=ROBUST
  /MISSING CLASSMISSING=EXCLUDE
  /PRINT CPS DESCRIPTIVES MODELINFO FIT SUMMARY SOLUTION.
```

Generalized Linear Models

Notes

Output Created		29-NOV-2023 19:53...
Comments		
Input	Data	/Users/yangshan/Desktop/2022-5-12/ /2023-10-OBL/2023-11-22 revised manuscript/OBL.sav
	Active Dataset	DataSet1
	Filter	<none>
	Weight	<none>
	Split File	<none>
	N of Rows in Working Data File	56
Missing Value Handling	Definition of Missing	User-defined missing values for factor, subject and within-subject variables are treated as missing.
	Cases Used	Statistics are based on cases with valid data for all variables in the model.
Weight Handling		not applicable

Notes

Syntax		GENLIN Trefoil1m3 BY group eyes (ORDER=ASCENDING)<br>/MODEL group eyes group*eyes INTERCEPT=YES<br><br>DISTRIBUTION=NORMAL LINK=IDENTITY<br>/CRITERIA SCALE=MLE PCONVERGE=1E-006 (ABSOLUTE) SINGULAR=1E-012 ANALYSISTYPE=3(WALD) CILEVEL=95<br>LIKELIHOOD=FULL<br>/REPEATED SUBJECT=patients WITHINSUBJECT=eyes SORT=YES CORRTYPE=INDEPENDENT ADJUSTCORR=YES<br>COVB=ROBUST<br>/MISSING CLASSMISSING=EXCLUDE<br>/PRINT CPS DESCRIPTIVES MODELINFO FIT SUMMARY SOLUTION.
Resources	Processor Time	00:00:00.04
	Elapsed Time	00:00:00.00

Model Information

Dependent Variable		Trefoil1m3
Probability Distribution		Normal
Link Function		Identity
Subject Effect	1	patients
Within-Subject Effect	1	eyes
Working Correlation Matrix Structure		Independent

Case Processing Summary

	N	Percent
Included	53	94.6%
Excluded	3	5.4%
Total	56	100.0%

Correlated Data Summary

Number of Levels	Subject Effect	patients	31
	Within-Subject Effect	eyes	2
Number of Subjects			31
Number of Measurements per Subject	Minimum		1
	Maximum		2
Correlation Matrix Dimension			2

Categorical Variable Information

			N	Percent
Factor	group	1	28	52.8%
		2	25	47.2%
		Total	53	100.0%
	eyes	1	29	54.7%
		2	24	45.3%
		Total	53	100.0%

Continuous Variable Information

		N	Minimum	Maximum	Mean	Std. Deviation
Dependent Variable	Trefoil1m3	53	.03	.26	.1075	.05997

Goodness of Fit[a]

	Value
Quasi Likelihood under Independence Model Criterion (QIC)[b]	8.181
Corrected Quasi Likelihood under Independence Model Criterion (QICC)[b]	8.181

Dependent Variable: Trefoil1m3
Model: (Intercept), group, eyes, group * eyes[a]

a. Information criteria are in smaller-is-better form.

b. Computed using the full log quasi-likelihood function.

Tests of Model Effects

Source	Type III		
	Wald Chi-Square	df	Sig.
(Intercept)	132.134	1	.000
group	.464	1	.496
eyes	1.132	1	.287
group * eyes	.460	1	.497

Dependent Variable: Trefoil1m3
Model: (Intercept), group, eyes, group * eyes

Parameter Estimates

Parameter	B	Std. Error	95% Wald Confidence Interval		Hypothesis ...
			Lower	Upper	Wald Chi-Square
(Intercept)	.126	.0187	.089	.163	45.302
[group=1]	-.022	.0243	-.069	.026	.794
[group=2]	0[a]	.	.	.	.
[eyes=1]	-.023	.0230	-.068	.022	.974
[eyes=2]	0[a]	.	.	.	.
[group=1] * [eyes=1]	.018	.0260	-.033	.069	.460
[group=1] * [eyes=2]	0[a]	.	.	.	.
[group=2] * [eyes=1]	0[a]	.	.	.	.
[group=2] * [eyes=2]	0[a]	.	.	.	.
(Scale)	.004				

Parameter Estimates

Parameter	Hypothesis Test	
	df	Sig.
(Intercept)	1	.000
[group=1]	1	.373
[group=2]	.	.
[eyes=1]	1	.324
[eyes=2]	.	.
[group=1] * [eyes=1]	1	.497
[group=1] * [eyes=2]	.	.
[group=2] * [eyes=1]	.	.
[group=2] * [eyes=2]	.	.
(Scale)		

Dependent Variable: Trefoil1m3
Model: (Intercept), group, eyes, group * eyes

a. Set to zero because this parameter is redundant.

```
* Generalized Estimating Equations.
GENLIN tHOAs3m3 BY group eyes (ORDER=ASCENDING)
  /MODEL group eyes group*eyes INTERCEPT=YES
 DISTRIBUTION=NORMAL LINK=IDENTITY
  /CRITERIA SCALE=MLE PCONVERGE=1E-006(ABSOLUTE) SINGULAR=1E-012 ANALYSISTY
PE=3(WALD) CILEVEL=95
    LIKELIHOOD=FULL
  /REPEATED SUBJECT=patients WITHINSUBJECT=eyes SORT=YES CORRTYPE=INDEPENDE
NT ADJUSTCORR=YES
    COVB=ROBUST
  /MISSING CLASSMISSING=EXCLUDE
  /PRINT CPS DESCRIPTIVES MODELINFO FIT SUMMARY SOLUTION.
```

Generalized Linear Models

Notes

Output Created		29-NOV-2023 19:53...
Comments		
Input	Data	/Users/yangshan/Desktop/2022-5-12//2023-10-OBL/2023-11-22 revised manuscript/OBL.sav
	Active Dataset	DataSet1
	Filter	<none>
	Weight	<none>
	Split File	<none>
	N of Rows in Working Data File	56
Missing Value Handling	Definition of Missing	User-defined missing values for factor, subject and within-subject variables are treated as missing.
	Cases Used	Statistics are based on cases with valid data for all variables in the model.
Weight Handling		not applicable

Notes

Syntax		GENLIN tHOAs3m3 BY group eyes (ORDER=ASCENDING)<br>/MODEL group eyes group*eyes INTERCEPT=YES<br><br>DISTRIBUTION=NORMAL LINK=IDENTITY<br>/CRITERIA SCALE=MLE PCONVERGE=1E-006 (ABSOLUTE) SINGULAR=1E-012 ANALYSISTYPE=3(WALD) CILEVEL=95<br>LIKELIHOOD=FULL<br>/REPEATED SUBJECT=patients WITHINSUBJECT=eyes SORT=YES CORRTYPE=INDEPENDENT ADJUSTCORR=YES<br>COVB=ROBUST<br>/MISSING CLASSMISSING=EXCLUDE<br>/PRINT CPS DESCRIPTIVES MODELINFO FIT SUMMARY SOLUTION.
Resources	Processor Time	00:00:00.04
	Elapsed Time	00:00:00.00

Model Information

Dependent Variable		tHOAs3m3
Probability Distribution		Normal
Link Function		Identity
Subject Effect	1	patients
Within-Subject Effect	1	eyes
Working Correlation Matrix Structure		Independent

Case Processing Summary

	N	Percent
Included	53	94.6%
Excluded	3	5.4%
Total	56	100.0%

Correlated Data Summary

Number of Levels	Subject Effect	patients	31
	Within-Subject Effect	eyes	2
Number of Subjects			31
Number of Measurements per Subject	Minimum		1
	Maximum		2
Correlation Matrix Dimension			2

Categorical Variable Information

			N	Percent
Factor	group	1	28	52.8%
		2	25	47.2%
		Total	53	100.0%
	eyes	1	29	54.7%
		2	24	45.3%
		Total	53	100.0%

Continuous Variable Information

		N	Minimum	Maximum	Mean	Std. Deviation
Dependent Variable	tHOAs3m3	53	.03	.26	.1075	.05997

Goodness of Fit[a]

	Value
Quasi Likelihood under Independence Model Criterion (QIC)[b]	8.181
Corrected Quasi Likelihood under Independence Model Criterion (QICC)[b]	8.181

Dependent Variable: tHOAs3m3
Model: (Intercept), group, eyes, group * eyes[a]

a. Information criteria are in smaller-is-better form.

b. Computed using the full log quasi-likelihood function.

Tests of Model Effects

Source	Type III		
	Wald Chi-Square	df	Sig.
(Intercept)	132.134	1	.000
group	.464	1	.496
eyes	1.132	1	.287
group * eyes	.460	1	.497

Dependent Variable: tHOAs3m3
Model: (Intercept), group, eyes, group * eyes

Parameter Estimates

Parameter	B	Std. Error	95% Wald Confidence Interval		Hypothesis ...
			Lower	Upper	Wald Chi-Square
(Intercept)	.126	.0187	.089	.163	45.302
[group=1]	-.022	.0243	-.069	.026	.794
[group=2]	0[a]	.	.	.	.
[eyes=1]	-.023	.0230	-.068	.022	.974
[eyes=2]	0[a]	.	.	.	.
[group=1] * [eyes=1]	.018	.0260	-.033	.069	.460
[group=1] * [eyes=2]	0[a]	.	.	.	.
[group=2] * [eyes=1]	0[a]	.	.	.	.
[group=2] * [eyes=2]	0[a]	.	.	.	.
(Scale)	.004				

Parameter Estimates

Parameter	Hypothesis Test	
	df	Sig.
(Intercept)	1	.000
[group=1]	1	.373
[group=2]	.	.
[eyes=1]	1	.324
[eyes=2]	.	.
[group=1] * [eyes=1]	1	.497
[group=1] * [eyes=2]	.	.
[group=2] * [eyes=1]	.	.
[group=2] * [eyes=2]	.	.
(Scale)		

Dependent Variable: tHOAs3m3
Model: (Intercept), group, eyes, group * eyes

a. Set to zero because this parameter is redundant.

```
 * Generalized Estimating Equations.
GENLIN Coma3m3 BY group eyes (ORDER=ASCENDING)
  /MODEL group eyes group*eyes INTERCEPT=YES
 DISTRIBUTION=NORMAL LINK=IDENTITY
  /CRITERIA SCALE=MLE PCONVERGE=1E-006(ABSOLUTE) SINGULAR=1E-012 ANALYSISTY
PE=3(WALD) CILEVEL=95
    LIKELIHOOD=FULL
  /REPEATED SUBJECT=patients WITHINSUBJECT=eyes SORT=YES CORRTYPE=INDEPENDE
NT ADJUSTCORR=YES
    COVB=ROBUST
  /MISSING CLASSMISSING=EXCLUDE
  /PRINT CPS DESCRIPTIVES MODELINFO FIT SUMMARY SOLUTION.
```

Generalized Linear Models

Notes

Output Created		29-NOV-2023 19:54...
Comments		
Input	Data	/Users/yangshan/Desktop/2022-5-12/ /2023-10-OBL/2023-11-22 revised manuscript/OBL .sav
	Active Dataset	DataSet1
	Filter	<none>
	Weight	<none>
	Split File	<none>
	N of Rows in Working Data File	56
Missing Value Handling	Definition of Missing	User-defined missing values for factor, subject and within-subject variables are treated as missing.
	Cases Used	Statistics are based on cases with valid data for all variables in the model.
Weight Handling		not applicable

Notes

Syntax		GENLIN Coma3m3 BY group eyes (ORDER=ASCENDING)<br>/MODEL group eyes group*eyes INTERCEPT=YES<br><br>DISTRIBUTION=NORMAL LINK=IDENTITY<br>/CRITERIA SCALE=MLE PCONVERGE=1E-006 (ABSOLUTE) SINGULAR=1E-012 ANALYSISTYPE=3(WALD) CILEVEL=95<br>LIKELIHOOD=FULL<br>/REPEATED SUBJECT=patients WITHINSUBJECT=eyes SORT=YES CORRTYPE=INDEPENDENT ADJUSTCORR=YES<br>COVB=ROBUST<br>/MISSING CLASSMISSING=EXCLUDE<br>/PRINT CPS DESCRIPTIVES MODELINFO FIT SUMMARY SOLUTION.
Resources	Processor Time	00:00:00.04
	Elapsed Time	00:00:00.00

Model Information

Dependent Variable		Coma3m3
Probability Distribution		Normal
Link Function		Identity
Subject Effect	1	patients
Within-Subject Effect	1	eyes
Working Correlation Matrix Structure		Independent

Case Processing Summary

	N	Percent
Included	53	94.6%
Excluded	3	5.4%
Total	56	100.0%

Correlated Data Summary

Number of Levels	Subject Effect	patients	31
	Within-Subject Effect	eyes	2
Number of Subjects			31
Number of Measurements per Subject	Minimum		1
	Maximum		2
Correlation Matrix Dimension			2

Categorical Variable Information

			N	Percent
Factor	group	1	28	52.8%
		2	25	47.2%
		Total	53	100.0%
	eyes	1	29	54.7%
		2	24	45.3%
		Total	53	100.0%

Continuous Variable Information

		N	Minimum	Maximum	Mean	Std. Deviation
Dependent Variable	Coma3m3	53	.01	.24	.0811	.06092

Goodness of Fit[a]

	Value
Quasi Likelihood under Independence Model Criterion (QIC)[b]	8.193
Corrected Quasi Likelihood under Independence Model Criterion (QICC)[b]	8.183

Dependent Variable: Coma3m3
Model: (Intercept), group, eyes, group * eyes[a]

a. Information criteria are in smaller-is-better form.

b. Computed using the full log quasi-likelihood function.

Tests of Model Effects

Source	Type III		
	Wald Chi-Square	df	Sig.
(Intercept)	77.556	1	.000
group	1.342	1	.247
eyes	.399	1	.527
group * eyes	1.609	1	.205

Dependent Variable: Coma3m3
Model: (Intercept), group, eyes, group * eyes

Parameter Estimates

Parameter	B	Std. Error	95% Wald Confidence Interval		Hypothesis ...
			Lower	Upper	Wald Chi-Square
(Intercept)	.105	.0188	.068	.142	31.345
[group=1]	-.039	.0241	-.086	.009	2.583
[group=2]	0[a]	.	.	.	.
[eyes=1]	-.026	.0243	-.073	.022	1.137
[eyes=2]	0[a]	.	.	.	.
[group=1] * [eyes=1]	.035	.0272	-.019	.088	1.609
[group=1] * [eyes=2]	0[a]	.	.	.	.
[group=2] * [eyes=1]	0[a]	.	.	.	.
[group=2] * [eyes=2]	0[a]	.	.	.	.
(Scale)	.004				

Parameter Estimates

Parameter	Hypothesis Test	
	df	Sig.
(Intercept)	1	.000
[group=1]	1	.108
[group=2]	.	.
[eyes=1]	1	.286
[eyes=2]	.	.
[group=1] * [eyes=1]	1	.205
[group=1] * [eyes=2]	.	.
[group=2] * [eyes=1]	.	.
[group=2] * [eyes=2]	.	.
(Scale)		

Dependent Variable: Coma3m3
Model: (Intercept), group, eyes, group * eyes

a. Set to zero because this parameter is redundant.

```
 * Generalized Estimating Equations.
GENLIN SA3m3 BY group eyes (ORDER=ASCENDING)
  /MODEL group eyes group*eyes INTERCEPT=YES
 DISTRIBUTION=NORMAL LINK=IDENTITY
  /CRITERIA SCALE=MLE PCONVERGE=1E-006(ABSOLUTE) SINGULAR=1E-012 ANALYSISTYPE=3(WALD) CILEVEL=95
    LIKELIHOOD=FULL
  /REPEATED SUBJECT=patients WITHINSUBJECT=eyes SORT=YES CORRTYPE=INDEPENDENT ADJUSTCORR=YES
    COVB=ROBUST
  /MISSING CLASSMISSING=EXCLUDE
  /PRINT CPS DESCRIPTIVES MODELINFO FIT SUMMARY SOLUTION.
```

Generalized Linear Models

Notes

Output Created		29-NOV-2023 19:54...
Comments		
Input	Data	/Users/yangshan/Desktop/2022-5-12//2023-10-OBL/2023-11-22 revised manuscript/OBL.sav
	Active Dataset	DataSet1
	Filter	<none>
	Weight	<none>
	Split File	<none>
	N of Rows in Working Data File	56
Missing Value Handling	Definition of Missing	User-defined missing values for factor, subject and within-subject variables are treated as missing.
	Cases Used	Statistics are based on cases with valid data for all variables in the model.
Weight Handling		not applicable

Notes

Syntax		GENLIN SA3m3 BY group eyes (ORDER=ASCENDING)<br>/MODEL group eyes group*eyes INTERCEPT=YES<br><br>DISTRIBUTION=NORMAL LINK=IDENTITY<br>/CRITERIA SCALE=MLE PCONVERGE=1E-006 (ABSOLUTE) SINGULAR=1E-012 ANALYSISTYPE=3(WALD) CILEVEL=95<br>LIKELIHOOD=FULL<br>/REPEATED SUBJECT=patients WITHINSUBJECT=eyes SORT=YES CORRTYPE=INDEPENDENT ADJUSTCORR=YES<br>COVB=ROBUST<br>/MISSING CLASSMISSING=EXCLUDE<br>/PRINT CPS DESCRIPTIVES MODELINFO FIT SUMMARY SOLUTION.
Resources	Processor Time	00:00:00.04
	Elapsed Time	00:00:00.00

Model Information

Dependent Variable		SA3m3
Probability Distribution		Normal
Link Function		Identity
Subject Effect	1	patients
Within-Subject Effect	1	eyes
Working Correlation Matrix Structure		Independent

Case Processing Summary

	N	Percent
Included	53	94.6%
Excluded	3	5.4%
Total	56	100.0%

Correlated Data Summary

Number of Levels	Subject Effect	patients	31
	Within-Subject Effect	eyes	2
Number of Subjects			31
Number of Measurements per Subject	Minimum		1
	Maximum		2
Correlation Matrix Dimension			2

Categorical Variable Information

			N	Percent
Factor	group	1	28	52.8%
		2	25	47.2%
		Total	53	100.0%
	eyes	1	29	54.7%
		2	24	45.3%
		Total	53	100.0%

Continuous Variable Information

		N	Minimum	Maximum	Mean	Std. Deviation
Dependent Variable	SA3m3	53	-.09	.07	.0014	.03141

Goodness of Fit[a]

	Value
Quasi Likelihood under Independence Model Criterion (QIC)[b]	8.227
Corrected Quasi Likelihood under Independence Model Criterion (QICC)[b]	8.050

Dependent Variable: SA3m3
Model: (Intercept), group, eyes, group * eyes[a]

a. Information criteria are in smaller-is-better form.

b. Computed using the full log quasi-likelihood function.

Tests of Model Effects

Source	Type III		
	Wald Chi-Square	df	Sig.
(Intercept)	.145	1	.703
group	.940	1	.332
eyes	.005	1	.943
group * eyes	.410	1	.522

Dependent Variable: SA3m3
Model: (Intercept), group, eyes, group * eyes

Parameter Estimates

Parameter	B	Std. Error	95% Wald Confidence Interval		Hypothesis ...
			Lower	Upper	Wald Chi-Square
(Intercept)	.005	.0087	-.013	.022	.267
[group=1]	-.005	.0143	-.033	.023	.111
[group=2]	0[a]	.	.	.	.
[eyes=1]	.004	.0108	-.017	.025	.150
[eyes=2]	0[a]	.	.	.	.
[group=1] * [eyes=1]	-.009	.0147	-.038	.019	.410
[group=1] * [eyes=2]	0[a]	.	.	.	.
[group=2] * [eyes=1]	0[a]	.	.	.	.
[group=2] * [eyes=2]	0[a]	.	.	.	.
(Scale)	.001				

Parameter Estimates

Parameter	Hypothesis Test	
	df	Sig.
(Intercept)	1	.605
[group=1]	1	.739
[group=2]	.	.
[eyes=1]	1	.699
[eyes=2]	.	.
[group=1] * [eyes=1]	1	.522
[group=1] * [eyes=2]	.	.
[group=2] * [eyes=1]	.	.
[group=2] * [eyes=2]	.	.
(Scale)		

Dependent Variable: SA3m3
Model: (Intercept), group, eyes, group * eyes

a. Set to zero because this parameter is redundant.

```
 * Generalized Estimating Equations.
GENLIN Trefoil3m3 BY group eyes (ORDER=ASCENDING)
  /MODEL group eyes group*eyes INTERCEPT=YES
 DISTRIBUTION=NORMAL LINK=IDENTITY
  /CRITERIA SCALE=MLE PCONVERGE=1E-006(ABSOLUTE) SINGULAR=1E-012 ANALYSISTYPE=3(WALD) CILEVEL=95
    LIKELIHOOD=FULL
  /REPEATED SUBJECT=patients WITHINSUBJECT=eyes SORT=YES CORRTYPE=INDEPENDENT ADJUSTCORR=YES
    COVB=ROBUST
  /MISSING CLASSMISSING=EXCLUDE
  /PRINT CPS DESCRIPTIVES MODELINFO FIT SUMMARY SOLUTION.
```

Generalized Linear Models

Notes

Output Created		29-NOV-2023 19:54...
Comments		
Input	Data	/Users/yangshan/Desktop/2022-5-12//2023-10-OBL/2023-11-22 revised manuscript/OBL.sav
	Active Dataset	DataSet1
	Filter	<none>
	Weight	<none>
	Split File	<none>
	N of Rows in Working Data File	56
Missing Value Handling	Definition of Missing	User-defined missing values for factor, subject and within-subject variables are treated as missing.
	Cases Used	Statistics are based on cases with valid data for all variables in the model.
Weight Handling		not applicable

Notes

Syntax		GENLIN Trefoil3m3 BY group eyes (ORDER=ASCENDING)<br>/MODEL group eyes group*eyes INTERCEPT=YES<br><br>DISTRIBUTION=NORMAL LINK=IDENTITY<br>/CRITERIA SCALE=MLE PCONVERGE=1E-006 (ABSOLUTE) SINGULAR=1E-012 ANALYSISTYPE=3(WALD) CILEVEL=95<br>LIKELIHOOD=FULL<br>/REPEATED SUBJECT=patients WITHINSUBJECT=eyes SORT=YES CORRTYPE=INDEPENDENT ADJUSTCORR=YES<br>COVB=ROBUST<br>/MISSING CLASSMISSING=EXCLUDE<br>/PRINT CPS DESCRIPTIVES MODELINFO FIT SUMMARY SOLUTION.
Resources	Processor Time	00:00:00.04
	Elapsed Time	00:00:00.00

Model Information

Dependent Variable		Trefoil3m3
Probability Distribution		Normal
Link Function		Identity
Subject Effect	1	patients
Within-Subject Effect	1	eyes
Working Correlation Matrix Structure		Independent

Case Processing Summary

	N	Percent
Included	53	94.6%
Excluded	3	5.4%
Total	56	100.0%

Correlated Data Summary

Number of Levels	Subject Effect	patients	31
	Within-Subject Effect	eyes	2
Number of Subjects			31
Number of Measurements per Subject	Minimum		1
	Maximum		2
Correlation Matrix Dimension			2

Categorical Variable Information

			N	Percent
Factor	group	1	28	52.8%
		2	25	47.2%
		Total	53	100.0%
	eyes	1	29	54.7%
		2	24	45.3%
		Total	53	100.0%

Continuous Variable Information

		N	Minimum	Maximum	Mean	Std. Deviation
Dependent Variable	Trefoil3m3	53	.00	.15	.0485	.03195

Goodness of Fit[a]

	Value
Quasi Likelihood under Independence Model Criterion (QIC)[b]	7.994
Corrected Quasi Likelihood under Independence Model Criterion (QICC)[b]	8.050

Dependent Variable: Trefoil3m3
Model: (Intercept), group, eyes, group * eyes[a]

a. Information criteria are in smaller-is-better form.

b. Computed using the full log quasi-likelihood function.

Tests of Model Effects

Source	Type III		
	Wald Chi-Square	df	Sig.
(Intercept)	108.967	1	.000
group	.960	1	.327
eyes	1.376	1	.241
group * eyes	1.523	1	.217

Dependent Variable: Trefoil3m3
Model: (Intercept), group, eyes, group * eyes

Parameter Estimates

Parameter	B	Std. Error	95% Wald Confidence Interval		Hypothesis ...
			Lower	Upper	Wald Chi-Square
(Intercept)	.044	.0091	.026	.062	23.373
[group=1]	.018	.0128	-.007	.044	2.038
[group=2]	0[a]	.	.	.	.
[eyes=1]	.000	.0121	-.023	.024	.001
[eyes=2]	0[a]	.	.	.	.
[group=1] * [eyes=1]	-.018	.0148	-.047	.011	1.523
[group=1] * [eyes=2]	0[a]	.	.	.	.
[group=2] * [eyes=1]	0[a]	.	.	.	.
[group=2] * [eyes=2]	0[a]	.	.	.	.
(Scale)	.001				

Parameter Estimates

Parameter	Hypothesis Test	
	df	Sig.
(Intercept)	1	.000
[group=1]	1	.153
[group=2]	.	.
[eyes=1]	1	.970
[eyes=2]	.	.
[group=1] * [eyes=1]	1	.217
[group=1] * [eyes=2]	.	.
[group=2] * [eyes=1]	.	.
[group=2] * [eyes=2]	.	.
(Scale)		

Dependent Variable: Trefoil3m3
Model: (Intercept), group, eyes, group * eyes

a. Set to zero because this parameter is redundant.

```
* Generalized Estimating Equations.
GENLIN PSF5 BY group eyes (ORDER=ASCENDING)
  /MODEL group eyes group*eyes INTERCEPT=YES
 DISTRIBUTION=NORMAL LINK=IDENTITY
  /CRITERIA SCALE=MLE PCONVERGE=1E-006(ABSOLUTE) SINGULAR=1E-012 ANALYSISTYPE=3(WALD) CILEVEL=95
    LIKELIHOOD=FULL
  /REPEATED SUBJECT=patients WITHINSUBJECT=eyes SORT=YES CORRTYPE=INDEPENDENT ADJUSTCORR=YES
    COVB=ROBUST
  /MISSING CLASSMISSING=EXCLUDE
  /PRINT CPS DESCRIPTIVES MODELINFO FIT SUMMARY SOLUTION.
```

Generalized Linear Models

Notes

Output Created		29-NOV-2023 19:54...
Comments		
Input	Data	/Users/yangshan/Desktop/2022-5-12//2023-10-OBL/2023-11-22 revised manuscript/OBL.sav
	Active Dataset	DataSet1
	Filter	<none>
	Weight	<none>
	Split File	<none>
	N of Rows in Working Data File	56
Missing Value Handling	Definition of Missing	User-defined missing values for factor, subject and within-subject variables are treated as missing.
	Cases Used	Statistics are based on cases with valid data for all variables in the model.
Weight Handling		not applicable

Notes

Syntax		GENLIN PSF5 BY group eyes (ORDER=ASCENDING)<br>/MODEL group eyes group*eyes INTERCEPT=YES<br><br>DISTRIBUTION=NORMAL LINK=IDENTITY<br>/CRITERIA SCALE=MLE PCONVERGE=1E-006 (ABSOLUTE) SINGULAR=1E-012 ANALYSISTYPE=3(WALD) CILEVEL=95<br>LIKELIHOOD=FULL<br>/REPEATED SUBJECT=patients WITHINSUBJECT=eyes SORT=YES CORRTYPE=INDEPENDENT ADJUSTCORR=YES<br>COVB=ROBUST<br>/MISSING CLASSMISSING=EXCLUDE<br>/PRINT CPS DESCRIPTIVES MODELINFO FIT SUMMARY SOLUTION.
Resources	Processor Time	00:00:00.04
	Elapsed Time	00:00:00.00

Model Information

Dependent Variable		PSF5
Probability Distribution		Normal
Link Function		Identity
Subject Effect	1	patients
Within-Subject Effect	1	eyes
Working Correlation Matrix Structure		Independent

Case Processing Summary

	N	Percent
Included	45	80.4%
Excluded	11	19.6%
Total	56	100.0%

Correlated Data Summary

Number of Levels	Subject Effect	patients	26
	Within-Subject Effect	eyes	2
Number of Subjects			26
Number of Measurements per Subject	Minimum		1
	Maximum		2
Correlation Matrix Dimension			2

Categorical Variable Information

			N	Percent
Factor	group	1	22	48.9%
		2	23	51.1%
		Total	45	100.0%
	eyes	1	26	57.8%
		2	19	42.2%
		Total	45	100.0%

Continuous Variable Information

		N	Minimum	Maximum	Mean	Std. Deviation
Dependent Variable	PSF5	45	.00	.77	.1056	.16958

Goodness of Fit[a]

	Value
Quasi Likelihood under Independence Model Criterion (QIC)[b]	9.390
Corrected Quasi Likelihood under Independence Model Criterion (QICC)[b]	9.254

Dependent Variable: PSF5
Model: (Intercept), group, eyes, group * eyes[a]

a. Information criteria are in smaller-is-better form.

b. Computed using the full log quasi-likelihood function.

Tests of Model Effects

Source	Type III		
	Wald Chi-Square	df	Sig.
(Intercept)	10.194	1	.001
group	.189	1	.663
eyes	.004	1	.947
group * eyes	.455	1	.500

Dependent Variable: PSF5
Model: (Intercept), group, eyes, group * eyes

Parameter Estimates

Parameter	B	Std. Error	95% Wald Confidence Interval		Hypothesis ...
			Lower	Upper	Wald Chi-Square
(Intercept)	.083	.0191	.045	.120	18.768
[group=1]	.049	.0785	-.105	.203	.386
[group=2]	0[a]	.	.	.	.
[eyes=1]	.018	.0395	-.059	.095	.205
[eyes=2]	0[a]	.	.	.	.
[group=1] * [eyes=1]	-.040	.0586	-.155	.075	.455
[group=1] * [eyes=2]	0[a]	.	.	.	.
[group=2] * [eyes=1]	0[a]	.	.	.	.
[group=2] * [eyes=2]	0[a]	.	.	.	.
(Scale)	.031				

Parameter Estimates

Parameter	Hypothesis Test	
	df	Sig.
(Intercept)	1	.000
[group=1]	1	.534
[group=2]	.	.
[eyes=1]	1	.651
[eyes=2]	.	.
[group=1] * [eyes=1]	1	.500
[group=1] * [eyes=2]	.	.
[group=2] * [eyes=1]	.	.
[group=2] * [eyes=2]	.	.
(Scale)		

Dependent Variable: PSF5
Model: (Intercept), group, eyes, group * eyes

a. Set to zero because this parameter is redundant.

```
 * Generalized Estimating Equations.
GENLIN MTF5 BY group eyes (ORDER=ASCENDING)
  /MODEL group eyes group*eyes INTERCEPT=YES
 DISTRIBUTION=NORMAL LINK=IDENTITY
  /CRITERIA SCALE=MLE PCONVERGE=1E-006(ABSOLUTE) SINGULAR=1E-012 ANALYSISTY
PE=3(WALD) CILEVEL=95
    LIKELIHOOD=FULL
  /REPEATED SUBJECT=patients WITHINSUBJECT=eyes SORT=YES CORRTYPE=INDEPENDE
NT ADJUSTCORR=YES
    COVB=ROBUST
  /MISSING CLASSMISSING=EXCLUDE
  /PRINT CPS DESCRIPTIVES MODELINFO FIT SUMMARY SOLUTION.
```

Generalized Linear Models

Notes

Output Created		29-NOV-2023 19:55...
Comments		
Input	Data	/Users/yangshan/Desktop/2022-5-12/ /2023-10-OBL/2023-11-22 revised manuscript/OBL .sav
	Active Dataset	DataSet1
	Filter	<none>
	Weight	<none>
	Split File	<none>
	N of Rows in Working Data File	56
Missing Value Handling	Definition of Missing	User-defined missing values for factor, subject and within-subject variables are treated as missing.
	Cases Used	Statistics are based on cases with valid data for all variables in the model.
Weight Handling		not applicable

Notes

Syntax		GENLIN MTF5 BY group eyes (ORDER=ASCENDING)<br>/MODEL group eyes group*eyes INTERCEPT=YES<br><br>DISTRIBUTION=NORMAL LINK=IDENTITY<br>/CRITERIA SCALE=MLE PCONVERGE=1E-006 (ABSOLUTE) SINGULAR=1E-012 ANALYSISTYPE=3(WALD) CILEVEL=95<br>LIKELIHOOD=FULL<br>/REPEATED SUBJECT=patients WITHINSUBJECT=eyes SORT=YES CORRTYPE=INDEPENDENT ADJUSTCORR=YES<br>COVB=ROBUST<br>/MISSING CLASSMISSING=EXCLUDE<br>/PRINT CPS DESCRIPTIVES MODELINFO FIT SUMMARY SOLUTION.
Resources	Processor Time	00:00:00.04
	Elapsed Time	00:00:00.00

Model Information

Dependent Variable		MTF5
Probability Distribution		Normal
Link Function		Identity
Subject Effect	1	patients
Within-Subject Effect	1	eyes
Working Correlation Matrix Structure		Independent

Case Processing Summary

	N	Percent
Included	41	73.2%
Excluded	15	26.8%
Total	56	100.0%

Correlated Data Summary

Number of Levels	Subject Effect	patients	23
	Within-Subject Effect	eyes	2
Number of Subjects			23
Number of Measurements per Subject	Minimum		1
	Maximum		2
Correlation Matrix Dimension			2

Categorical Variable Information

			N	Percent
Factor	group	1	19	46.3%
		2	22	53.7%
		Total	41	100.0%
	eyes	1	23	56.1%
		2	18	43.9%
		Total	41	100.0%

Continuous Variable Information

		N	Minimum	Maximum	Mean	Std. Deviation
Dependent Variable	MTF5	41	.14	.60	.3073	.11103

Goodness of Fit[a]

	Value
Quasi Likelihood under Independence Model Criterion (QIC)[b]	8.730
Corrected Quasi Likelihood under Independence Model Criterion (QICC)[b]	8.487

Dependent Variable: MTF5
Model: (Intercept), group, eyes, group * eyes[a]

a. Information criteria are in smaller-is-better form.

b. Computed using the full log quasi-likelihood function.

Tests of Model Effects

Source	Type III		
	Wald Chi-Square	df	Sig.
(Intercept)	185.262	1	.000
group	.001	1	.970
eyes	.010	1	.921
group * eyes	1.132	1	.287

Dependent Variable: MTF5
Model: (Intercept), group, eyes, group * eyes

Parameter Estimates

Parameter	B	Std. Error	95% Wald Confidence Interval		Hypothesis ...
			Lower	Upper	Wald Chi-Square
(Intercept)	.295	.0239	.249	.342	153.050
[group=1]	.022	.0545	-.085	.129	.162
[group=2]	0[a]	.	.	.	.
[eyes=1]	.026	.0277	-.028	.080	.874
[eyes=2]	0[a]	.	.	.	.
[group=1] * [eyes=1]	-.047	.0445	-.134	.040	1.132
[group=1] * [eyes=2]	0[a]	.	.	.	.
[group=2] * [eyes=1]	0[a]	.	.	.	.
[group=2] * [eyes=2]	0[a]	.	.	.	.
(Scale)	.013				

Parameter Estimates

Parameter	Hypothesis Test	
	df	Sig.
(Intercept)	1	.000
[group=1]	1	.687
[group=2]	.	.
[eyes=1]	1	.350
[eyes=2]	.	.
[group=1] * [eyes=1]	1	.287
[group=1] * [eyes=2]	.	.
[group=2] * [eyes=1]	.	.
[group=2] * [eyes=2]	.	.
(Scale)		

Dependent Variable: MTF5
Model: (Intercept), group, eyes, group * eyes

a. Set to zero because this parameter is redundant.

```
 * Generalized Estimating Equations.
GENLIN PSF3 BY group eyes (ORDER=ASCENDING)
  /MODEL group eyes group*eyes INTERCEPT=YES
 DISTRIBUTION=NORMAL LINK=IDENTITY
  /CRITERIA SCALE=MLE PCONVERGE=1E-006(ABSOLUTE) SINGULAR=1E-012 ANALYSISTY
PE=3(WALD) CILEVEL=95
    LIKELIHOOD=FULL
  /REPEATED SUBJECT=patients WITHINSUBJECT=eyes SORT=YES CORRTYPE=INDEPENDE
NT ADJUSTCORR=YES
    COVB=ROBUST
  /MISSING CLASSMISSING=EXCLUDE
  /PRINT CPS DESCRIPTIVES MODELINFO FIT SUMMARY SOLUTION.
```

Generalized Linear Models

Notes

Output Created		29-NOV-2023 19:55...
Comments		
Input	Data	/Users/yangshan/Desktop/2022-5-12/ /2023-10-OBL/2023-11-22 revised manuscript/OBL .sav
	Active Dataset	DataSet1
	Filter	<none>
	Weight	<none>
	Split File	<none>
	N of Rows in Working Data File	56
Missing Value Handling	Definition of Missing	User-defined missing values for factor, subject and within-subject variables are treated as missing.
	Cases Used	Statistics are based on cases with valid data for all variables in the model.
Weight Handling		not applicable

Notes

Syntax		GENLIN PSF3 BY group eyes (ORDER=ASCENDING)<br>/MODEL group eyes group*eyes INTERCEPT=YES<br><br>DISTRIBUTION=NORMAL LINK=IDENTITY<br>/CRITERIA SCALE=MLE PCONVERGE=1E-006 (ABSOLUTE) SINGULAR=1E-012 ANALYSISTYPE=3(WALD) CILEVEL=95<br>LIKELIHOOD=FULL<br>/REPEATED SUBJECT=patients WITHINSUBJECT=eyes SORT=YES CORRTYPE=INDEPENDENT ADJUSTCORR=YES<br>COVB=ROBUST<br>/MISSING CLASSMISSING=EXCLUDE<br>/PRINT CPS DESCRIPTIVES MODELINFO FIT SUMMARY SOLUTION.
Resources	Processor Time	00:00:00.04
	Elapsed Time	00:00:00.00

Model Information

Dependent Variable		PSF3
Probability Distribution		Normal
Link Function		Identity
Subject Effect	1	patients
Within-Subject Effect	1	eyes
Working Correlation Matrix Structure		Independent

Case Processing Summary

	N	Percent
Included	45	80.4%
Excluded	11	19.6%
Total	56	100.0%

Correlated Data Summary

Number of Levels	Subject Effect	patients	26
	Within-Subject Effect	eyes	2
Number of Subjects			26
Number of Measurements per Subject	Minimum		1
	Maximum		2
Correlation Matrix Dimension			2

Categorical Variable Information

			N	Percent
Factor	group	1	22	48.9%
		2	23	51.1%
		Total	45	100.0%
	eyes	1	26	57.8%
		2	19	42.2%
		Total	45	100.0%

Continuous Variable Information

		N	Minimum	Maximum	Mean	Std. Deviation
Dependent Variable	PSF3	45	.01	.64	.1398	.12494

Goodness of Fit[a]

	Value
Quasi Likelihood under Independence Model Criterion (QIC)[b]	9.187
Corrected Quasi Likelihood under Independence Model Criterion (QICC)[b]	8.663

Dependent Variable: PSF3
Model: (Intercept), group, eyes, group * eyes[a]

a. Information criteria are in smaller-is-better form.

b. Computed using the full log quasi-likelihood function.

Tests of Model Effects

Source	Type III		
	Wald Chi-Square	df	Sig.
(Intercept)	37.874	1	.000
group	.995	1	.319
eyes	.001	1	.970
group * eyes	.365	1	.546

Dependent Variable: PSF3
Model: (Intercept), group, eyes, group * eyes

Parameter Estimates

Parameter	B	Std. Error	95% Wald Confidence Interval		Hypothesis ...
			Lower	Upper	Wald Chi-Square
(Intercept)	.109	.0188	.072	.146	33.429
[group=1]	.064	.0657	-.064	.193	.962
[group=2]	0[a]	.	.	.	.
[eyes=1]	.018	.0250	-.031	.067	.500
[eyes=2]	0[a]	.	.	.	.
[group=1] * [eyes=1]	-.038	.0625	-.160	.085	.365
[group=1] * [eyes=2]	0[a]	.	.	.	.
[group=2] * [eyes=1]	0[a]	.	.	.	.
[group=2] * [eyes=2]	0[a]	.	.	.	.
(Scale)	.016				

Parameter Estimates

Parameter	Hypothesis Test	
	df	Sig.
(Intercept)	1	.000
[group=1]	1	.327
[group=2]	.	.
[eyes=1]	1	.479
[eyes=2]	.	.
[group=1] * [eyes=1]	1	.546
[group=1] * [eyes=2]	.	.
[group=2] * [eyes=1]	.	.
[group=2] * [eyes=2]	.	.
(Scale)		

Dependent Variable: PSF3
Model: (Intercept), group, eyes, group * eyes

a. Set to zero because this parameter is redundant.

```
 * Generalized Estimating Equations.
GENLIN MTF3 BY group eyes (ORDER=ASCENDING)
  /MODEL group eyes group*eyes INTERCEPT=YES
 DISTRIBUTION=NORMAL LINK=IDENTITY
  /CRITERIA SCALE=MLE PCONVERGE=1E-006(ABSOLUTE) SINGULAR=1E-012 ANALYSISTY
PE=3(WALD) CILEVEL=95
    LIKELIHOOD=FULL
  /REPEATED SUBJECT=patients WITHINSUBJECT=eyes SORT=YES CORRTYPE=INDEPENDE
NT ADJUSTCORR=YES
    COVB=ROBUST
  /MISSING CLASSMISSING=EXCLUDE
  /PRINT CPS DESCRIPTIVES MODELINFO FIT SUMMARY SOLUTION.
```

Generalized Linear Models

Notes

Output Created		29-NOV-2023 19:55...
Comments		
Input	Data	/Users/yangshan/Desktop/2022-5-12//2023-10-OBL/2023-11-22 revised manuscript/OBL.sav
	Active Dataset	DataSet1
	Filter	<none>
	Weight	<none>
	Split File	<none>
	N of Rows in Working Data File	56
Missing Value Handling	Definition of Missing	User-defined missing values for factor, subject and within-subject variables are treated as missing.
	Cases Used	Statistics are based on cases with valid data for all variables in the model.
Weight Handling		not applicable

Notes

Syntax		GENLIN MTF3 BY group eyes (ORDER=ASCENDING)<br>/MODEL group eyes group*eyes INTERCEPT=YES<br><br>DISTRIBUTION=NORMAL LINK=IDENTITY<br>/CRITERIA SCALE=MLE PCONVERGE=1E-006 (ABSOLUTE) SINGULAR=1E-012 ANALYSISTYPE=3(WALD) CILEVEL=95<br>LIKELIHOOD=FULL<br>/REPEATED SUBJECT=patients WITHINSUBJECT=eyes SORT=YES CORRTYPE=INDEPENDENT ADJUSTCORR=YES<br>COVB=ROBUST<br>/MISSING CLASSMISSING=EXCLUDE<br>/PRINT CPS DESCRIPTIVES MODELINFO FIT SUMMARY SOLUTION.
Resources	Processor Time	00:00:00.03
	Elapsed Time	00:00:00.00

Model Information

Dependent Variable		MTF3
Probability Distribution		Normal
Link Function		Identity
Subject Effect	1	patients
Within-Subject Effect	1	eyes
Working Correlation Matrix Structure		Independent

Case Processing Summary

	N	Percent
Included	41	73.2%
Excluded	15	26.8%
Total	56	100.0%

Correlated Data Summary

Number of Levels	Subject Effect	patients	23
	Within-Subject Effect	eyes	2
Number of Subjects			23
Number of Measurements per Subject	Minimum		1
	Maximum		2
Correlation Matrix Dimension			2

Categorical Variable Information

			N	Percent
Factor	group	1	19	46.3%
		2	22	53.7%
		Total	41	100.0%
	eyes	1	23	56.1%
		2	18	43.9%
		Total	41	100.0%

Continuous Variable Information

		N	Minimum	Maximum	Mean	Std. Deviation
Dependent Variable	MTF3	41	.15	.60	.3419	.10717

Goodness of Fit[a]

	Value
Quasi Likelihood under Independence Model Criterion (QIC)[b]	9.011
Corrected Quasi Likelihood under Independence Model Criterion (QICC)[b]	8.449

Dependent Variable: MTF3
Model: (Intercept), group, eyes, group * eyes[a]

a. Information criteria are in smaller-is-better form.

b. Computed using the full log quasi-likelihood function.

Tests of Model Effects

Source	Type III		
	Wald Chi-Square	df	Sig.
(Intercept)	256.862	1	.000
group	.269	1	.604
eyes	.124	1	.725
group * eyes	.671	1	.413

Dependent Variable: MTF3
Model: (Intercept), group, eyes, group * eyes

Parameter Estimates

Parameter	B	Std. Error	95% Wald Confidence Interval		Hypothesis ...
			Lower	Upper	Wald Chi-Square
(Intercept)	.316	.0240	.269	.363	172.484
[group=1]	.044	.0593	-.072	.160	.546
[group=2]	0[a]	.	.	.	.
[eyes=1]	.031	.0386	-.045	.107	.644
[eyes=2]	0[a]	.	.	.	.
[group=1] * [eyes=1]	-.043	.0529	-.147	.060	.671
[group=1] * [eyes=2]	0[a]	.	.	.	.
[group=2] * [eyes=1]	0[a]	.	.	.	.
[group=2] * [eyes=2]	0[a]	.	.	.	.
(Scale)	.012				

Parameter Estimates

Parameter	Hypothesis Test	
	df	Sig.
(Intercept)	1	.000
[group=1]	1	.460
[group=2]	.	.
[eyes=1]	1	.422
[eyes=2]	.	.
[group=1] * [eyes=1]	1	.413
[group=1] * [eyes=2]	.	.
[group=2] * [eyes=1]	.	.
[group=2] * [eyes=2]	.	.
(Scale)		

Dependent Variable: MTF3
Model: (Intercept), group, eyes, group * eyes

a. Set to zero because this parameter is redundant.

```
 * Generalized Estimating Equations.
GENLIN PSF51w BY group eyes (ORDER=ASCENDING)
  /MODEL group eyes group*eyes INTERCEPT=YES
 DISTRIBUTION=NORMAL LINK=IDENTITY
  /CRITERIA SCALE=MLE PCONVERGE=1E-006(ABSOLUTE) SINGULAR=1E-012 ANALYSISTY
PE=3(WALD) CILEVEL=95
    LIKELIHOOD=FULL
  /REPEATED SUBJECT=patients WITHINSUBJECT=eyes SORT=YES CORRTYPE=INDEPENDE
NT ADJUSTCORR=YES
    COVB=ROBUST
  /MISSING CLASSMISSING=EXCLUDE
  /PRINT CPS DESCRIPTIVES MODELINFO FIT SUMMARY SOLUTION.
```

Generalized Linear Models

Notes

Output Created		29-NOV-2023 19:55...
Comments		
Input	Data	/Users/yangshan/Desktop/2022-5-12/ /2023-10-OBL/2023-11-22 revised manuscript/OBL .sav
	Active Dataset	DataSet1
	Filter	<none>
	Weight	<none>
	Split File	<none>
	N of Rows in Working Data File	56
Missing Value Handling	Definition of Missing	User-defined missing values for factor, subject and within-subject variables are treated as missing.
	Cases Used	Statistics are based on cases with valid data for all variables in the model.
Weight Handling		not applicable

Notes

Syntax		GENLIN PSF51w BY group eyes (ORDER=ASCENDING)<br>/MODEL group eyes group*eyes INTERCEPT=YES<br><br>DISTRIBUTION=NORMAL LINK=IDENTITY<br>/CRITERIA SCALE=MLE PCONVERGE=1E-006 (ABSOLUTE) SINGULAR=1E-012 ANALYSISTYPE=3(WALD) CILEVEL=95<br>LIKELIHOOD=FULL<br>/REPEATED SUBJECT=patients WITHINSUBJECT=eyes SORT=YES CORRTYPE=INDEPENDENT ADJUSTCORR=YES<br>COVB=ROBUST<br>/MISSING CLASSMISSING=EXCLUDE<br>/PRINT CPS DESCRIPTIVES MODELINFO FIT SUMMARY SOLUTION.
Resources	Processor Time	00:00:00.04
	Elapsed Time	00:00:00.00

Model Information

Dependent Variable		PSF51w
Probability Distribution		Normal
Link Function		Identity
Subject Effect	1	patients
Within-Subject Effect	1	eyes
Working Correlation Matrix Structure		Independent

Case Processing Summary

	N	Percent
Included	43	76.8%
Excluded	13	23.2%
Total	56	100.0%

Correlated Data Summary

Number of Levels	Subject Effect	patients	25
	Within-Subject Effect	eyes	2
Number of Subjects			25
Number of Measurements per Subject	Minimum		1
	Maximum		2
Correlation Matrix Dimension			2

Categorical Variable Information

			N	Percent
Factor	group	1	20	46.5%
		2	23	53.5%
		Total	43	100.0%
	eyes	1	25	58.1%
		2	18	41.9%
		Total	43	100.0%

Continuous Variable Information

		N	Minimum	Maximum	Mean	Std. Deviation
Dependent Variable	PSF51w	43	.01	.52	.1031	.09685

Goodness of Fit[a]

	Value
Quasi Likelihood under Independence Model Criterion (QIC)[b]	8.397
Corrected Quasi Likelihood under Independence Model Criterion (QICC)[b]	8.388

Dependent Variable: PSF51w
Model: (Intercept), group, eyes, group * eyes[a]

a. Information criteria are in smaller-is-better form.

b. Computed using the full log quasi-likelihood function.

Tests of Model Effects

Source	Type III		
	Wald Chi-Square	df	Sig.
(Intercept)	40.700	1	.000
group	.129	1	.719
eyes	.143	1	.706
group * eyes	.648	1	.421

Dependent Variable: PSF51w
Model: (Intercept), group, eyes, group * eyes

Parameter Estimates

Parameter	B	Std. Error	95% Wald Confidence Interval		Hypothesis ...
			Lower	Upper	Wald Chi-Square
(Intercept)	.113	.0446	.026	.200	6.423
[group=1]	-.033	.0479	-.127	.061	.464
[group=2]	0[a]	.	.	.	.
[eyes=1]	-.011	.0444	-.098	.076	.064
[eyes=2]	0[a]	.	.	.	.
[group=1] * [eyes=1]	.042	.0527	-.061	.146	.648
[group=1] * [eyes=2]	0[a]	.	.	.	.
[group=2] * [eyes=1]	0[a]	.	.	.	.
[group=2] * [eyes=2]	0[a]	.	.	.	.
(Scale)	.010				

Parameter Estimates

Parameter	Hypothesis Test	
	df	Sig.
(Intercept)	1	.011
[group=1]	1	.496
[group=2]	.	.
[eyes=1]	1	.800
[eyes=2]	.	.
[group=1] * [eyes=1]	1	.421
[group=1] * [eyes=2]	.	.
[group=2] * [eyes=1]	.	.
[group=2] * [eyes=2]	.	.
(Scale)		

Dependent Variable: PSF51w
Model: (Intercept), group, eyes, group * eyes

a. Set to zero because this parameter is redundant.

```
 * Generalized Estimating Equations.
GENLIN MTF51w BY group eyes (ORDER=ASCENDING)
  /MODEL group eyes group*eyes INTERCEPT=YES
 DISTRIBUTION=NORMAL LINK=IDENTITY
  /CRITERIA SCALE=MLE PCONVERGE=1E-006(ABSOLUTE) SINGULAR=1E-012 ANALYSISTY
PE=3(WALD) CILEVEL=95
    LIKELIHOOD=FULL
  /REPEATED SUBJECT=patients WITHINSUBJECT=eyes SORT=YES CORRTYPE=INDEPENDE
NT ADJUSTCORR=YES
    COVB=ROBUST
  /MISSING CLASSMISSING=EXCLUDE
  /PRINT CPS DESCRIPTIVES MODELINFO FIT SUMMARY SOLUTION.
```

Generalized Linear Models

Notes

Output Created		29-NOV-2023 19:55...
Comments		
Input	Data	/Users/yangshan/Desktop/2022-5-12/ /2023-10-OBL/2023-11-22 revised manuscript/OBL.sav
	Active Dataset	DataSet1
	Filter	<none>
	Weight	<none>
	Split File	<none>
	N of Rows in Working Data File	56
Missing Value Handling	Definition of Missing	User-defined missing values for factor, subject and within-subject variables are treated as missing.
	Cases Used	Statistics are based on cases with valid data for all variables in the model.
Weight Handling		not applicable

Notes

Syntax		GENLIN MTF51w BY group eyes (ORDER=ASCENDING)<br>/MODEL group eyes group*eyes INTERCEPT=YES<br><br>DISTRIBUTION=NORMAL LINK=IDENTITY<br>/CRITERIA SCALE=MLE PCONVERGE=1E-006 (ABSOLUTE) SINGULAR=1E-012 ANALYSISTYPE=3(WALD) CILEVEL=95<br>LIKELIHOOD=FULL<br>/REPEATED SUBJECT=patients WITHINSUBJECT=eyes SORT=YES CORRTYPE=INDEPENDENT ADJUSTCORR=YES<br>COVB=ROBUST<br>/MISSING CLASSMISSING=EXCLUDE<br>/PRINT CPS DESCRIPTIVES MODELINFO FIT SUMMARY SOLUTION.
Resources	Processor Time	00:00:00.03
	Elapsed Time	00:00:00.00

Model Information

Dependent Variable		MTF51w
Probability Distribution		Normal
Link Function		Identity
Subject Effect	1	patients
Within-Subject Effect	1	eyes
Working Correlation Matrix Structure		Independent

Case Processing Summary

	N	Percent
Included	43	76.8%
Excluded	13	23.2%
Total	56	100.0%

Correlated Data Summary

Number of Levels	Subject Effect	patients	25
	Within-Subject Effect	eyes	2
Number of Subjects			25
Number of Measurements per Subject	Minimum		1
	Maximum		2
Correlation Matrix Dimension			2

Categorical Variable Information

			N	Percent
Factor	group	1	20	46.5%
		2	23	53.5%
		Total	43	100.0%
	eyes	1	25	58.1%
		2	18	41.9%
		Total	43	100.0%

Continuous Variable Information

		N	Minimum	Maximum	Mean	Std. Deviation
Dependent Variable	MTF51w	43	.22	.63	.3632	.09516

Goodness of Fit[a]

	Value
Quasi Likelihood under Independence Model Criterion (QIC)[b]	8.517
Corrected Quasi Likelihood under Independence Model Criterion (QICC)[b]	8.365

Dependent Variable: MTF51w
Model: (Intercept), group, eyes, group * eyes[a]

a. Information criteria are in smaller-is-better form.

b. Computed using the full log quasi-likelihood function.

Tests of Model Effects

Source	Type III		
	Wald Chi-Square	df	Sig.
(Intercept)	451.901	1	.000
group	.751	1	.386
eyes	.453	1	.501
group * eyes	.362	1	.547

Dependent Variable: MTF51w
Model: (Intercept), group, eyes, group * eyes

Parameter Estimates

Parameter	B	Std. Error	95% Wald Confidence Interval		Hypothesis ...
			Lower	Upper	Wald Chi-Square
(Intercept)	.347	.0244	.299	.395	202.154
[group=1]	.016	.0439	-.070	.102	.128
[group=2]	0[a]	.	.	.	.
[eyes=1]	.002	.0317	-.061	.064	.003
[eyes=2]	0[a]	.	.	.	.
[group=1] * [eyes=1]	.028	.0462	-.063	.118	.362
[group=1] * [eyes=2]	0[a]	.	.	.	.
[group=2] * [eyes=1]	0[a]	.	.	.	.
[group=2] * [eyes=2]	0[a]	.	.	.	.
(Scale)	.009				

Parameter Estimates

Parameter	Hypothesis Test	
	df	Sig.
(Intercept)	1	.000
[group=1]	1	.721
[group=2]	.	.
[eyes=1]	1	.959
[eyes=2]	.	.
[group=1] * [eyes=1]	1	.547
[group=1] * [eyes=2]	.	.
[group=2] * [eyes=1]	.	.
[group=2] * [eyes=2]	.	.
(Scale)		

Dependent Variable: MTF51w
Model: (Intercept), group, eyes, group * eyes

a. Set to zero because this parameter is redundant.

```
 * Generalized Estimating Equations.
GENLIN PSF31w BY group eyes (ORDER=ASCENDING)
  /MODEL group eyes group*eyes INTERCEPT=YES
 DISTRIBUTION=NORMAL LINK=IDENTITY
  /CRITERIA SCALE=MLE PCONVERGE=1E-006(ABSOLUTE) SINGULAR=1E-012 ANALYSISTY
PE=3(WALD) CILEVEL=95
    LIKELIHOOD=FULL
  /REPEATED SUBJECT=patients WITHINSUBJECT=eyes SORT=YES CORRTYPE=INDEPENDE
NT ADJUSTCORR=YES
    COVB=ROBUST
  /MISSING CLASSMISSING=EXCLUDE
  /PRINT CPS DESCRIPTIVES MODELINFO FIT SUMMARY SOLUTION.
```

Generalized Linear Models

Notes

Output Created		29-NOV-2023 19:56...
Comments		
Input	Data	/Users/yangshan/Desktop/2022-5-12//2023-10-OBL/2023-11-22 revised manuscript/OBL.sav
	Active Dataset	DataSet1
	Filter	<none>
	Weight	<none>
	Split File	<none>
	N of Rows in Working Data File	56
Missing Value Handling	Definition of Missing	User-defined missing values for factor, subject and within-subject variables are treated as missing.
	Cases Used	Statistics are based on cases with valid data for all variables in the model.
Weight Handling		not applicable

Notes

Syntax		GENLIN PSF31w BY group eyes (ORDER=ASCENDING)<br>/MODEL group eyes group*eyes INTERCEPT=YES<br>DISTRIBUTION=NORMAL LINK=IDENTITY<br>/CRITERIA SCALE=MLE PCONVERGE=1E-006 (ABSOLUTE) SINGULAR=1E-012 ANALYSISTYPE=3(WALD) CILEVEL=95<br>LIKELIHOOD=FULL<br>/REPEATED SUBJECT=patients WITHINSUBJECT=eyes SORT=YES CORRTYPE=INDEPENDENT ADJUSTCORR=YES<br>COVB=ROBUST<br>/MISSING CLASSMISSING=EXCLUDE<br>/PRINT CPS DESCRIPTIVES MODELINFO FIT SUMMARY SOLUTION.
Resources	Processor Time	00:00:00.04
	Elapsed Time	00:00:01.00

Model Information

Dependent Variable		PSF31w
Probability Distribution		Normal
Link Function		Identity
Subject Effect	1	patients
Within-Subject Effect	1	eyes
Working Correlation Matrix Structure		Independent

Case Processing Summary

	N	Percent
Included	43	76.8%
Excluded	13	23.2%
Total	56	100.0%

Correlated Data Summary

Number of Levels	Subject Effect	patients	25
	Within-Subject Effect	eyes	2
Number of Subjects			25
Number of Measurements per Subject	Minimum		1
	Maximum		2
Correlation Matrix Dimension			2

Categorical Variable Information

			N	Percent
Factor	group	1	20	46.5%
		2	23	53.5%
		Total	43	100.0%
	eyes	1	25	58.1%
		2	18	41.9%
		Total	43	100.0%

Continuous Variable Information

		N	Minimum	Maximum	Mean	Std. Deviation
Dependent Variable	PSF31w	43	.05	.56	.2159	.14048

Goodness of Fit[a]

	Value
Quasi Likelihood under Independence Model Criterion (QIC)[b]	8.565
Corrected Quasi Likelihood under Independence Model Criterion (QICC)[b]	8.814

Dependent Variable: PSF31w
Model: (Intercept), group, eyes, group * eyes[a]

a. Information criteria are in smaller-is-better form.

b. Computed using the full log quasi-likelihood function.

Tests of Model Effects

Source	Type III		
	Wald Chi-Square	df	Sig.
(Intercept)	93.267	1	.000
group	.347	1	.556
eyes	.319	1	.572
group * eyes	.103	1	.748

Dependent Variable: PSF31w
Model: (Intercept), group, eyes, group * eyes

Parameter Estimates

Parameter	B	Std. Error	95% Wald Confidence Interval		Hypothesis ...
			Lower	Upper	Wald Chi-Square
(Intercept)	.197	.0369	.125	.270	28.573
[group=1]	.014	.0568	-.097	.125	.061
[group=2]	0[a]	.	.	.	.
[eyes=1]	.009	.0545	-.098	.116	.029
[eyes=2]	0[a]	.	.	.	.
[group=1] * [eyes=1]	.024	.0757	-.124	.173	.103
[group=1] * [eyes=2]	0[a]	.	.	.	.
[group=2] * [eyes=1]	0[a]	.	.	.	.
[group=2] * [eyes=2]	0[a]	.	.	.	.
(Scale)	.021				

Parameter Estimates

Parameter	Hypothesis Test	
	df	Sig.
(Intercept)	1	.000
[group=1]	1	.804
[group=2]	.	.
[eyes=1]	1	.866
[eyes=2]	.	.
[group=1] * [eyes=1]	1	.748
[group=1] * [eyes=2]	.	.
[group=2] * [eyes=1]	.	.
[group=2] * [eyes=2]	.	.
(Scale)		

Dependent Variable: PSF31w
Model: (Intercept), group, eyes, group * eyes

a. Set to zero because this parameter is redundant.

```
 * Generalized Estimating Equations.
GENLIN MTF31w BY group eyes (ORDER=ASCENDING)
  /MODEL group eyes group*eyes INTERCEPT=YES
 DISTRIBUTION=NORMAL LINK=IDENTITY
  /CRITERIA SCALE=MLE PCONVERGE=1E-006(ABSOLUTE) SINGULAR=1E-012 ANALYSISTY
PE=3(WALD) CILEVEL=95
    LIKELIHOOD=FULL
  /REPEATED SUBJECT=patients WITHINSUBJECT=eyes SORT=YES CORRTYPE=INDEPENDE
NT ADJUSTCORR=YES
    COVB=ROBUST
  /MISSING CLASSMISSING=EXCLUDE
  /PRINT CPS DESCRIPTIVES MODELINFO FIT SUMMARY SOLUTION.
```

Generalized Linear Models

Notes

Output Created		29-NOV-2023 19:56...
Comments		
Input	Data	/Users/yangshan/Desktop/2022-5-12/ /2023-10-OBL/2023-11-22 revised manuscript/OBL .sav
	Active Dataset	DataSet1
	Filter	<none>
	Weight	<none>
	Split File	<none>
	N of Rows in Working Data File	56
Missing Value Handling	Definition of Missing	User-defined missing values for factor, subject and within-subject variables are treated as missing.
	Cases Used	Statistics are based on cases with valid data for all variables in the model.
Weight Handling		not applicable

Notes

Syntax		GENLIN MTF31w BY group eyes (ORDER=ASCENDING)<br>/MODEL group eyes group*eyes INTERCEPT=YES<br><br>DISTRIBUTION=NORMAL LINK=IDENTITY<br>/CRITERIA SCALE=MLE PCONVERGE=1E-006 (ABSOLUTE) SINGULAR=1E-012 ANALYSISTYPE=3(WALD) CILEVEL=95<br>LIKELIHOOD=FULL<br>/REPEATED SUBJECT=patients WITHINSUBJECT=eyes SORT=YES CORRTYPE=INDEPENDENT ADJUSTCORR=YES<br>COVB=ROBUST<br>/MISSING CLASSMISSING=EXCLUDE<br>/PRINT CPS DESCRIPTIVES MODELINFO FIT SUMMARY SOLUTION.
Resources	Processor Time	00:00:00.04
	Elapsed Time	00:00:00.00

Model Information

Dependent Variable		MTF31w
Probability Distribution		Normal
Link Function		Identity
Subject Effect	1	patients
Within-Subject Effect	1	eyes
Working Correlation Matrix Structure		Independent

Case Processing Summary

	N	Percent
Included	43	76.8%
Excluded	13	23.2%
Total	56	100.0%

Correlated Data Summary

Number of Levels	Subject Effect	patients	25
	Within-Subject Effect	eyes	2
Number of Subjects			25
Number of Measurements per Subject	Minimum		1
	Maximum		2
Correlation Matrix Dimension			2

Categorical Variable Information

			N	Percent
Factor	group	1	20	46.5%
		2	23	53.5%
		Total	43	100.0%
	eyes	1	25	58.1%
		2	18	41.9%
		Total	43	100.0%

Continuous Variable Information

		N	Minimum	Maximum	Mean	Std. Deviation
Dependent Variable	MTF31w	43	.03	.72	.3201	.17718

Goodness of Fit[a]

	Value
Quasi Likelihood under Independence Model Criterion (QIC)[b]	8.476
Corrected Quasi Likelihood under Independence Model Criterion (QICC)[b]	8.717

Dependent Variable: MTF31w
Model: (Intercept), group, eyes, group * eyes[a]

a. Information criteria are in smaller-is-better form.

b. Computed using the full log quasi-likelihood function.

Tests of Model Effects

Source	Type III		
	Wald Chi-Square	df	Sig.
(Intercept)	234.868	1	.000
group	29.748	1	.000
eyes	1.195	1	.274
group * eyes	.053	1	.818

Dependent Variable: MTF31w
Model: (Intercept), group, eyes, group * eyes

Parameter Estimates

Parameter	B	Std. Error	95% Wald Confidence Interval		Hypothesis ...
			Lower	Upper	Wald Chi-Square
(Intercept)	.194	.0340	.128	.261	32.613
[group=1]	.223	.0551	.115	.331	16.390
[group=2]	0[a]	.	.	.	.
[eyes=1]	.030	.0576	-.083	.143	.276
[eyes=2]	0[a]	.	.	.	.
[group=1] * [eyes=1]	.016	.0702	-.121	.154	.053
[group=1] * [eyes=2]	0[a]	.	.	.	.
[group=2] * [eyes=1]	0[a]	.	.	.	.
[group=2] * [eyes=2]	0[a]	.	.	.	.
(Scale)	.018				

Parameter Estimates

Parameter	Hypothesis Test	
	df	Sig.
(Intercept)	1	.000
[group=1]	1	.000
[group=2]	.	.
[eyes=1]	1	.599
[eyes=2]	.	.
[group=1] * [eyes=1]	1	.818
[group=1] * [eyes=2]	.	.
[group=2] * [eyes=1]	.	.
[group=2] * [eyes=2]	.	.
(Scale)		

Dependent Variable: MTF31w
Model: (Intercept), group, eyes, group * eyes

a. Set to zero because this parameter is redundant.

```
 * Generalized Estimating Equations.
GENLIN PSF51m BY group eyes (ORDER=ASCENDING)
  /MODEL group eyes group*eyes INTERCEPT=YES
 DISTRIBUTION=NORMAL LINK=IDENTITY
  /CRITERIA SCALE=MLE PCONVERGE=1E-006(ABSOLUTE) SINGULAR=1E-012 ANALYSISTY
PE=3(WALD) CILEVEL=95
    LIKELIHOOD=FULL
  /REPEATED SUBJECT=patients WITHINSUBJECT=eyes SORT=YES CORRTYPE=INDEPENDE
NT ADJUSTCORR=YES
    COVB=ROBUST
  /MISSING CLASSMISSING=EXCLUDE
  /PRINT CPS DESCRIPTIVES MODELINFO FIT SUMMARY SOLUTION.
```

Generalized Linear Models

Notes

Output Created		29-NOV-2023 19:56...
Comments		
Input	Data	/Users/yangshan/Desktop/2022-5-12/ /2023-10-OBL/2023-11-22 revised manuscript/OBL .sav
	Active Dataset	DataSet1
	Filter	<none>
	Weight	<none>
	Split File	<none>
	N of Rows in Working Data File	56
Missing Value Handling	Definition of Missing	User-defined missing values for factor, subject and within-subject variables are treated as missing.
	Cases Used	Statistics are based on cases with valid data for all variables in the model.
Weight Handling		not applicable

Notes

Syntax		GENLIN PSF51m BY group eyes (ORDER=ASCENDING)<br>/MODEL group eyes group*eyes INTERCEPT=YES<br><br>DISTRIBUTION=NORMAL LINK=IDENTITY<br>/CRITERIA SCALE=MLE PCONVERGE=1E-006 (ABSOLUTE) SINGULAR=1E-012 ANALYSISTYPE=3(WALD) CILEVEL=95<br>LIKELIHOOD=FULL<br>/REPEATED SUBJECT=patients WITHINSUBJECT=eyes SORT=YES CORRTYPE=INDEPENDENT ADJUSTCORR=YES<br>COVB=ROBUST<br>/MISSING CLASSMISSING=EXCLUDE<br>/PRINT CPS DESCRIPTIVES MODELINFO FIT SUMMARY SOLUTION.
Resources	Processor Time	00:00:00.04
	Elapsed Time	00:00:00.00

Model Information

Dependent Variable		PSF51m
Probability Distribution		Normal
Link Function		Identity
Subject Effect	1	patients
Within-Subject Effect	1	eyes
Working Correlation Matrix Structure		Independent

Case Processing Summary

	N	Percent
Included	42	75.0%
Excluded	14	25.0%
Total	56	100.0%

Correlated Data Summary

Number of Levels	Subject Effect	patients	25
	Within-Subject Effect	eyes	2
Number of Subjects			25
Number of Measurements per Subject	Minimum		1
	Maximum		2
Correlation Matrix Dimension			2

Categorical Variable Information

			N	Percent
Factor	group	1	19	45.2%
		2	23	54.8%
		Total	42	100.0%
	eyes	1	24	57.1%
		2	18	42.9%
		Total	42	100.0%

Continuous Variable Information

		N	Minimum	Maximum	Mean	Std. Deviation
Dependent Variable	PSF51m	42	.02	.64	.1467	.13545

Goodness of Fit[a]

	Value
Quasi Likelihood under Independence Model Criterion (QIC)[b]	8.686
Corrected Quasi Likelihood under Independence Model Criterion (QICC)[b]	8.698

Dependent Variable: PSF51m
Model: (Intercept), group, eyes, group * eyes[a]

a. Information criteria are in smaller-is-better form.

b. Computed using the full log quasi-likelihood function.

Tests of Model Effects

Source	Type III		
	Wald Chi-Square	df	Sig.
(Intercept)	50.242	1	.000
group	1.749	1	.186
eyes	.275	1	.600
group * eyes	1.564	1	.211

Dependent Variable: PSF51m
Model: (Intercept), group, eyes, group * eyes

Parameter Estimates

Parameter	B	Std. Error	95% Wald Confidence Interval		Hypothesis ...
			Lower	Upper	Wald Chi-Square
(Intercept)	.207	.0644	.081	.333	10.338
[group=1]	-.103	.0659	-.232	.026	2.431
[group=2]	0[a]	.	.	.	.
[eyes=1]	-.069	.0692	-.204	.067	.985
[eyes=2]	0[a]	.	.	.	.
[group=1] * [eyes=1]	.097	.0774	-.055	.248	1.564
[group=1] * [eyes=2]	0[a]	.	.	.	.
[group=2] * [eyes=1]	0[a]	.	.	.	.
[group=2] * [eyes=2]	0[a]	.	.	.	.
(Scale)	.018				

Parameter Estimates

Parameter	Hypothesis Test	
	df	Sig.
(Intercept)	1	.001
[group=1]	1	.119
[group=2]	.	.
[eyes=1]	1	.321
[eyes=2]	.	.
[group=1] * [eyes=1]	1	.211
[group=1] * [eyes=2]	.	.
[group=2] * [eyes=1]	.	.
[group=2] * [eyes=2]	.	.
(Scale)		

Dependent Variable: PSF51m
Model: (Intercept), group, eyes, group * eyes

a. Set to zero because this parameter is redundant.

```
 * Generalized Estimating Equations.
GENLIN MTF51m BY group eyes (ORDER=ASCENDING)
  /MODEL group eyes group*eyes INTERCEPT=YES
 DISTRIBUTION=NORMAL LINK=IDENTITY
  /CRITERIA SCALE=MLE PCONVERGE=1E-006(ABSOLUTE) SINGULAR=1E-012 ANALYSISTYPE=3(WALD) CILEVEL=95
    LIKELIHOOD=FULL
  /REPEATED SUBJECT=patients WITHINSUBJECT=eyes SORT=YES CORRTYPE=INDEPENDENT ADJUSTCORR=YES
    COVB=ROBUST
  /MISSING CLASSMISSING=EXCLUDE
  /PRINT CPS DESCRIPTIVES MODELINFO FIT SUMMARY SOLUTION.
```

Generalized Linear Models

Notes

Output Created		29-NOV-2023 19:56...
Comments		
Input	Data	/Users/yangshan/Desktop/2022-5-12/ /2023-10-OBL/2023-11-22 revised manuscript/OBL .sav
	Active Dataset	DataSet1
	Filter	<none>
	Weight	<none>
	Split File	<none>
	N of Rows in Working Data File	56
Missing Value Handling	Definition of Missing	User-defined missing values for factor, subject and within-subject variables are treated as missing.
	Cases Used	Statistics are based on cases with valid data for all variables in the model.
Weight Handling		not applicable

Notes

Syntax		GENLIN MTF51m BY group eyes (ORDER=ASCENDING)<br>/MODEL group eyes group*eyes INTERCEPT=YES<br><br>DISTRIBUTION=NORMAL LINK=IDENTITY<br>/CRITERIA SCALE=MLE PCONVERGE=1E-006 (ABSOLUTE) SINGULAR=1E-012 ANALYSISTYPE=3(WALD) CILEVEL=95<br>LIKELIHOOD=FULL<br>/REPEATED SUBJECT=patients WITHINSUBJECT=eyes SORT=YES CORRTYPE=INDEPENDENT ADJUSTCORR=YES<br>COVB=ROBUST<br>/MISSING CLASSMISSING=EXCLUDE<br>/PRINT CPS DESCRIPTIVES MODELINFO FIT SUMMARY SOLUTION.
Resources	Processor Time	00:00:00.04
	Elapsed Time	00:00:00.00

Model Information

Dependent Variable		MTF51m
Probability Distribution		Normal
Link Function		Identity
Subject Effect	1	patients
Within-Subject Effect	1	eyes
Working Correlation Matrix Structure		Independent

Case Processing Summary

	N	Percent
Included	42	75.0%
Excluded	14	25.0%
Total	56	100.0%

Correlated Data Summary

Number of Levels	Subject Effect	patients	25
	Within-Subject Effect	eyes	2
Number of Subjects			25
Number of Measurements per Subject	Minimum		1
	Maximum		2
Correlation Matrix Dimension			2

Categorical Variable Information

			N	Percent
Factor	group	1	19	45.2%
		2	23	54.8%
		Total	42	100.0%
	eyes	1	24	57.1%
		2	18	42.9%
		Total	42	100.0%

Continuous Variable Information

		N	Minimum	Maximum	Mean	Std. Deviation
Dependent Variable	MTF51m	42	.21	.63	.3693	.09595

Goodness of Fit[a]

	Value
Quasi Likelihood under Independence Model Criterion (QIC)[b]	8.228
Corrected Quasi Likelihood under Independence Model Criterion (QICC)[b]	8.373

Dependent Variable: MTF51m
Model: (Intercept), group, eyes, group * eyes[a]

a. Information criteria are in smaller-is-better form.

b. Computed using the full log quasi-likelihood function.

Tests of Model Effects

Source	Type III		
	Wald Chi-Square	df	Sig.
(Intercept)	513.214	1	.000
group	.010	1	.921
eyes	.638	1	.424
group * eyes	.069	1	.792

Dependent Variable: MTF51m
Model: (Intercept), group, eyes, group * eyes

Parameter Estimates

Parameter	B	Std. Error	95% Wald Confidence Interval		Hypothesis ...
			Lower	Upper	Wald Chi-Square
(Intercept)	.360	.0304	.300	.419	140.141
[group=1]	-.003	.0404	-.083	.076	.007
[group=2]	0[a]	.	.	.	.
[eyes=1]	.013	.0354	-.056	.083	.141
[eyes=2]	0[a]	.	.	.	.
[group=1] * [eyes=1]	.013	.0496	-.084	.110	.069
[group=1] * [eyes=2]	0[a]	.	.	.	.
[group=2] * [eyes=1]	0[a]	.	.	.	.
[group=2] * [eyes=2]	0[a]	.	.	.	.
(Scale)	.010				

Parameter Estimates

Parameter	Hypothesis Test	
	df	Sig.
(Intercept)	1	.000
[group=1]	1	.935
[group=2]	.	.
[eyes=1]	1	.708
[eyes=2]	.	.
[group=1] * [eyes=1]	1	.792
[group=1] * [eyes=2]	.	.
[group=2] * [eyes=1]	.	.
[group=2] * [eyes=2]	.	.
(Scale)		

Dependent Variable: MTF51m
Model: (Intercept), group, eyes, group * eyes

a. Set to zero because this parameter is redundant.

```
 * Generalized Estimating Equations.
GENLIN PSF31m BY group eyes (ORDER=ASCENDING)
  /MODEL group eyes group*eyes INTERCEPT=YES
 DISTRIBUTION=NORMAL LINK=IDENTITY
  /CRITERIA SCALE=MLE PCONVERGE=1E-006(ABSOLUTE) SINGULAR=1E-012 ANALYSISTYPE=3(WALD) CILEVEL=95
    LIKELIHOOD=FULL
  /REPEATED SUBJECT=patients WITHINSUBJECT=eyes SORT=YES CORRTYPE=INDEPENDENT ADJUSTCORR=YES
    COVB=ROBUST
  /MISSING CLASSMISSING=EXCLUDE
  /PRINT CPS DESCRIPTIVES MODELINFO FIT SUMMARY SOLUTION.
```

Generalized Linear Models

Notes

Output Created		29-NOV-2023 19:56...
Comments		
Input	Data	/Users/yangshan/Desktop/2022-5-12/ /2023-10-OBL/2023-11-22 revised manuscript/OBL .sav
	Active Dataset	DataSet1
	Filter	<none>
	Weight	<none>
	Split File	<none>
	N of Rows in Working Data File	56
Missing Value Handling	Definition of Missing	User-defined missing values for factor, subject and within-subject variables are treated as missing.
	Cases Used	Statistics are based on cases with valid data for all variables in the model.
Weight Handling		not applicable

Notes

Syntax		GENLIN PSF31m BY group eyes (ORDER=ASCENDING)<br>/MODEL group eyes group*eyes INTERCEPT=YES<br><br>DISTRIBUTION=NORMAL LINK=IDENTITY<br>/CRITERIA SCALE=MLE PCONVERGE=1E-006 (ABSOLUTE) SINGULAR=1E-012 ANALYSISTYPE=3(WALD) CILEVEL=95<br>LIKELIHOOD=FULL<br>/REPEATED SUBJECT=patients WITHINSUBJECT=eyes SORT=YES CORRTYPE=INDEPENDENT ADJUSTCORR=YES<br>COVB=ROBUST<br>/MISSING CLASSMISSING=EXCLUDE<br>/PRINT CPS DESCRIPTIVES MODELINFO FIT SUMMARY SOLUTION.
Resources	Processor Time	00:00:00.05
	Elapsed Time	00:00:00.00

Model Information

Dependent Variable		PSF31m
Probability Distribution		Normal
Link Function		Identity
Subject Effect	1	patients
Within-Subject Effect	1	eyes
Working Correlation Matrix Structure		Independent

Case Processing Summary

	N	Percent
Included	43	76.8%
Excluded	13	23.2%
Total	56	100.0%

Correlated Data Summary

Number of Levels	Subject Effect	patients	25
	Within-Subject Effect	eyes	2
Number of Subjects			25
Number of Measurements per Subject	Minimum		1
	Maximum		2
Correlation Matrix Dimension			2

Categorical Variable Information

			N	Percent
Factor	group	1	20	46.5%
		2	23	53.5%
		Total	43	100.0%
	eyes	1	25	58.1%
		2	18	41.9%
		Total	43	100.0%

Continuous Variable Information

		N	Minimum	Maximum	Mean	Std. Deviation
Dependent Variable	PSF31m	43	.03	.77	.2257	.16554

Goodness of Fit[a]

	Value
Quasi Likelihood under Independence Model Criterion (QIC)[b]	8.771
Corrected Quasi Likelihood under Independence Model Criterion (QICC)[b]	9.121

Dependent Variable: PSF31m
Model: (Intercept), group, eyes, group * eyes[a]

a. Information criteria are in smaller-is-better form.

b. Computed using the full log quasi-likelihood function.

Tests of Model Effects

Source	Type III		
	Wald Chi-Square	df	Sig.
(Intercept)	65.724	1	.000
group	.251	1	.616
eyes	1.163	1	.281
group * eyes	.095	1	.758

Dependent Variable: PSF31m
Model: (Intercept), group, eyes, group * eyes

Parameter Estimates

Parameter	B	Std. Error	95% Wald Confidence Interval		Hypothesis ...
			Lower	Upper	Wald Chi-Square
(Intercept)	.194	.0340	.128	.261	32.613
[group=1]	.015	.0596	-.101	.132	.067
[group=2]	0[a]	.	.	.	.
[eyes=1]	.030	.0576	-.083	.143	.276
[eyes=2]	0[a]	.	.	.	.
[group=1] * [eyes=1]	.024	.0786	-.130	.178	.095
[group=1] * [eyes=2]	0[a]	.	.	.	.
[group=2] * [eyes=1]	0[a]	.	.	.	.
[group=2] * [eyes=2]	0[a]	.	.	.	.
(Scale)	.029				

Parameter Estimates

Parameter	Hypothesis Test	
	df	Sig.
(Intercept)	1	.000
[group=1]	1	.795
[group=2]	.	.
[eyes=1]	1	.599
[eyes=2]	.	.
[group=1] * [eyes=1]	1	.758
[group=1] * [eyes=2]	.	.
[group=2] * [eyes=1]	.	.
[group=2] * [eyes=2]	.	.
(Scale)		

Dependent Variable: PSF31m
Model: (Intercept), group, eyes, group * eyes

a. Set to zero because this parameter is redundant.

```
 * Generalized Estimating Equations.
GENLIN MTF31m BY group eyes (ORDER=ASCENDING)
  /MODEL group eyes group*eyes INTERCEPT=YES
 DISTRIBUTION=NORMAL LINK=IDENTITY
  /CRITERIA SCALE=MLE PCONVERGE=1E-006(ABSOLUTE) SINGULAR=1E-012 ANALYSISTY
PE=3(WALD) CILEVEL=95
    LIKELIHOOD=FULL
  /REPEATED SUBJECT=patients WITHINSUBJECT=eyes SORT=YES CORRTYPE=INDEPENDE
NT ADJUSTCORR=YES
    COVB=ROBUST
  /MISSING CLASSMISSING=EXCLUDE
  /PRINT CPS DESCRIPTIVES MODELINFO FIT SUMMARY SOLUTION.
```

Generalized Linear Models

Notes

Output Created		29-NOV-2023 19:57...
Comments		
Input	Data	/Users/yangshan/Desktop/2022-5-12//2023-10-OBL/2023-11-22 revised manuscript/OBL.sav
	Active Dataset	DataSet1
	Filter	<none>
	Weight	<none>
	Split File	<none>
	N of Rows in Working Data File	56
Missing Value Handling	Definition of Missing	User-defined missing values for factor, subject and within-subject variables are treated as missing.
	Cases Used	Statistics are based on cases with valid data for all variables in the model.
Weight Handling		not applicable

Notes

Syntax		GENLIN MTF31m BY group eyes (ORDER=ASCENDING)<br>/MODEL group eyes group*eyes INTERCEPT=YES<br><br>DISTRIBUTION=NORMAL LINK=IDENTITY<br>/CRITERIA SCALE=MLE PCONVERGE=1E-006 (ABSOLUTE) SINGULAR=1E-012 ANALYSISTYPE=3(WALD) CILEVEL=95<br>LIKELIHOOD=FULL<br>/REPEATED SUBJECT=patients WITHINSUBJECT=eyes SORT=YES CORRTYPE=INDEPENDENT ADJUSTCORR=YES<br>COVB=ROBUST<br>/MISSING CLASSMISSING=EXCLUDE<br>/PRINT CPS DESCRIPTIVES MODELINFO FIT SUMMARY SOLUTION.
Resources	Processor Time	00:00:00.04
	Elapsed Time	00:00:01.00

Model Information

Dependent Variable		MTF31m
Probability Distribution		Normal
Link Function		Identity
Subject Effect	1	patients
Within-Subject Effect	1	eyes
Working Correlation Matrix Structure		Independent

Case Processing Summary

	N	Percent
Included	41	73.2%
Excluded	15	26.8%
Total	56	100.0%

Correlated Data Summary

Number of Levels	Subject Effect	patients	24
	Within-Subject Effect	eyes	2
Number of Subjects			24
Number of Measurements per Subject	Minimum		1
	Maximum		2
Correlation Matrix Dimension			2

Categorical Variable Information

			N	Percent
Factor	group	1	20	48.8%
		2	21	51.2%
		Total	41	100.0%
	eyes	1	24	58.5%
		2	17	41.5%
		Total	41	100.0%

Continuous Variable Information

		N	Minimum	Maximum	Mean	Std. Deviation
Dependent Variable	MTF31m	41	.03	.89	.3258	.22142

Goodness of Fit[a]

	Value
Quasi Likelihood under Independence Model Criterion (QIC)[b]	9.844
Corrected Quasi Likelihood under Independence Model Criterion (QICC)[b]	9.785

Dependent Variable: MTF31m
Model: (Intercept), group, eyes, group * eyes[a]

a. Information criteria are in smaller-is-better form.

b. Computed using the full log quasi-likelihood function.

Tests of Model Effects

Source	Type III		
	Wald Chi-Square	df	Sig.
(Intercept)	94.732	1	.000
group	2.953	1	.086
eyes	1.186	1	.276
group * eyes	.027	1	.870

Dependent Variable: MTF31m
Model: (Intercept), group, eyes, group * eyes

Parameter Estimates

Parameter	B	Std. Error	95% Wald Confidence Interval		Hypothesis ...
			Lower	Upper	Wald Chi-Square
(Intercept)	.342	.0204	.302	.382	282.047
[group=1]	-.123	.1003	-.320	.073	1.514
[group=2]	0[a]	.	.	.	.
[eyes=1]	.064	.0492	-.033	.160	1.668
[eyes=2]	0[a]	.	.	.	.
[group=1] * [eyes=1]	.022	.1371	-.246	.291	.027
[group=1] * [eyes=2]	0[a]	.	.	.	.
[group=2] * [eyes=1]	0[a]	.	.	.	.
[group=2] * [eyes=2]	0[a]	.	.	.	.
(Scale)	.048				

Parameter Estimates

Parameter	Hypothesis Test	
	df	Sig.
(Intercept)	1	.000
[group=1]	1	.219
[group=2]	.	.
[eyes=1]	1	.197
[eyes=2]	.	.
[group=1] * [eyes=1]	1	.870
[group=1] * [eyes=2]	.	.
[group=2] * [eyes=1]	.	.
[group=2] * [eyes=2]	.	.
(Scale)		

Dependent Variable: MTF31m
Model: (Intercept), group, eyes, group * eyes

a. Set to zero because this parameter is redundant.

```
 * Generalized Estimating Equations.
GENLIN PSF53m BY group eyes (ORDER=ASCENDING)
  /MODEL group eyes group*eyes INTERCEPT=YES
 DISTRIBUTION=NORMAL LINK=IDENTITY
  /CRITERIA SCALE=MLE PCONVERGE=1E-006(ABSOLUTE) SINGULAR=1E-012 ANALYSISTY
PE=3(WALD) CILEVEL=95
    LIKELIHOOD=FULL
  /REPEATED SUBJECT=patients WITHINSUBJECT=eyes SORT=YES CORRTYPE=INDEPENDE
NT ADJUSTCORR=YES
    COVB=ROBUST
  /MISSING CLASSMISSING=EXCLUDE
  /PRINT CPS DESCRIPTIVES MODELINFO FIT SUMMARY SOLUTION.
```

Generalized Linear Models

Notes

Output Created		29-NOV-2023 19:57...
Comments		
Input	Data	/Users/yangshan/Desktop/2022-5-12/ /2023-10-OBL/2023-11-22 revised manuscript/OBL.sav
	Active Dataset	DataSet1
	Filter	<none>
	Weight	<none>
	Split File	<none>
	N of Rows in Working Data File	56
Missing Value Handling	Definition of Missing	User-defined missing values for factor, subject and within-subject variables are treated as missing.
	Cases Used	Statistics are based on cases with valid data for all variables in the model.
Weight Handling		not applicable

Notes

Syntax		GENLIN PSF53m BY group eyes (ORDER=ASCENDING)<br>/MODEL group eyes group*eyes INTERCEPT=YES<br><br>DISTRIBUTION=NORMAL LINK=IDENTITY<br>/CRITERIA SCALE=MLE PCONVERGE=1E-006 (ABSOLUTE) SINGULAR=1E-012 ANALYSISTYPE=3(WALD) CILEVEL=95<br>LIKELIHOOD=FULL<br>/REPEATED SUBJECT=patients WITHINSUBJECT=eyes SORT=YES CORRTYPE=INDEPENDENT ADJUSTCORR=YES<br>COVB=ROBUST<br>/MISSING CLASSMISSING=EXCLUDE<br>/PRINT CPS DESCRIPTIVES MODELINFO FIT SUMMARY SOLUTION.
Resources	Processor Time	00:00:00.04
	Elapsed Time	00:00:00.00

Model Information

Dependent Variable		PSF53m
Probability Distribution		Normal
Link Function		Identity
Subject Effect	1	patients
Within-Subject Effect	1	eyes
Working Correlation Matrix Structure		Independent

Case Processing Summary

	N	Percent
Included	41	73.2%
Excluded	15	26.8%
Total	56	100.0%

Correlated Data Summary

Number of Levels	Subject Effect	patients	24
	Within-Subject Effect	eyes	2
Number of Subjects			24
Number of Measurements per Subject	Minimum		1
	Maximum		2
Correlation Matrix Dimension			2

Categorical Variable Information

			N	Percent
Factor	group	1	20	48.8%
		2	21	51.2%
		Total	41	100.0%
	eyes	1	24	58.5%
		2	17	41.5%
		Total	41	100.0%

Continuous Variable Information

		N	Minimum	Maximum	Mean	Std. Deviation
Dependent Variable	PSF53m	41	.02	.89	.1996	.24261

Goodness of Fit[a]

	Value
Quasi Likelihood under Independence Model Criterion (QIC)[b]	10.099
Corrected Quasi Likelihood under Independence Model Criterion (QICC)[b]	10.116

Dependent Variable: PSF53m
Model: (Intercept), group, eyes, group * eyes[a]

a. Information criteria are in smaller-is-better form.

b. Computed using the full log quasi-likelihood function.

Tests of Model Effects

Source	Type III		
	Wald Chi-Square	df	Sig.
(Intercept)	30.256	1	.000
group	3.457	1	.063
eyes	.696	1	.404
group * eyes	.115	1	.734

Dependent Variable: PSF53m
Model: (Intercept), group, eyes, group * eyes

Parameter Estimates

Parameter	B	Std. Error	95% Wald Confidence Interval		Hypothesis ...
			Lower	Upper	Wald Chi-Square
(Intercept)	.111	.0417	.030	.193	7.142
[group=1]	.107	.1067	-.102	.317	1.014
[group=2]	0[a]	.	.	.	.
[eyes=1]	.036	.0708	-.102	.175	.262
[eyes=2]	0[a]	.	.	.	.
[group=1] * [eyes=1]	.050	.1463	-.237	.336	.115
[group=1] * [eyes=2]	0[a]	.	.	.	.
[group=2] * [eyes=1]	0[a]	.	.	.	.
[group=2] * [eyes=2]	0[a]	.	.	.	.
(Scale)	.057				

Parameter Estimates

Parameter	Hypothesis Test	
	df	Sig.
(Intercept)	1	.008
[group=1]	1	.314
[group=2]	.	.
[eyes=1]	1	.609
[eyes=2]	.	.
[group=1] * [eyes=1]	1	.734
[group=1] * [eyes=2]	.	.
[group=2] * [eyes=1]	.	.
[group=2] * [eyes=2]	.	.
(Scale)		

Dependent Variable: PSF53m
Model: (Intercept), group, eyes, group * eyes

a. Set to zero because this parameter is redundant.

```
 * Generalized Estimating Equations.
GENLIN MTF53m BY group eyes (ORDER=ASCENDING)
  /MODEL group eyes group*eyes INTERCEPT=YES
 DISTRIBUTION=NORMAL LINK=IDENTITY
  /CRITERIA SCALE=MLE PCONVERGE=1E-006(ABSOLUTE) SINGULAR=1E-012 ANALYSISTY
PE=3(WALD) CILEVEL=95
    LIKELIHOOD=FULL
  /REPEATED SUBJECT=patients WITHINSUBJECT=eyes SORT=YES CORRTYPE=INDEPENDE
NT ADJUSTCORR=YES
    COVB=ROBUST
  /MISSING CLASSMISSING=EXCLUDE
  /PRINT CPS DESCRIPTIVES MODELINFO FIT SUMMARY SOLUTION.
```

Generalized Linear Models

Notes

Output Created		29-NOV-2023 19:57...
Comments		
Input	Data	/Users/yangshan/Desktop/2022-5-12//2023-10-OBL/2023-11-22 revised manuscript/OBL.sav
	Active Dataset	DataSet1
	Filter	<none>
	Weight	<none>
	Split File	<none>
	N of Rows in Working Data File	56
Missing Value Handling	Definition of Missing	User-defined missing values for factor, subject and within-subject variables are treated as missing.
	Cases Used	Statistics are based on cases with valid data for all variables in the model.
Weight Handling		not applicable

Notes

Syntax		GENLIN MTF53m BY group eyes (ORDER=ASCENDING)<br>/MODEL group eyes group*eyes INTERCEPT=YES<br><br>DISTRIBUTION=NORMAL LINK=IDENTITY<br>/CRITERIA SCALE=MLE PCONVERGE=1E-006 (ABSOLUTE) SINGULAR=1E-012 ANALYSISTYPE=3(WALD) CILEVEL=95<br>LIKELIHOOD=FULL<br>/REPEATED SUBJECT=patients WITHINSUBJECT=eyes SORT=YES CORRTYPE=INDEPENDENT ADJUSTCORR=YES<br>COVB=ROBUST<br>/MISSING CLASSMISSING=EXCLUDE<br>/PRINT CPS DESCRIPTIVES MODELINFO FIT SUMMARY SOLUTION.
Resources	Processor Time	00:00:00.04
	Elapsed Time	00:00:00.00

Model Information

Dependent Variable		MTF53m
Probability Distribution		Normal
Link Function		Identity
Subject Effect	1	patients
Within-Subject Effect	1	eyes
Working Correlation Matrix Structure		Independent

Case Processing Summary

	N	Percent
Included	41	73.2%
Excluded	15	26.8%
Total	56	100.0%

Correlated Data Summary

Number of Levels	Subject Effect	patients	24
	Within-Subject Effect	eyes	2
Number of Subjects			24
Number of Measurements per Subject	Minimum		1
	Maximum		2
Correlation Matrix Dimension			2

Categorical Variable Information

			N	Percent
Factor	group	1	20	48.8%
		2	21	51.2%
		Total	41	100.0%
	eyes	1	24	58.5%
		2	17	41.5%
		Total	41	100.0%

Continuous Variable Information

		N	Minimum	Maximum	Mean	Std. Deviation
Dependent Variable	MTF53m	41	.31	.84	.5047	.11551

Goodness of Fit[a]

	Value
Quasi Likelihood under Independence Model Criterion (QIC)[b]	8.324
Corrected Quasi Likelihood under Independence Model Criterion (QICC)[b]	8.456

Dependent Variable: MTF53m
Model: (Intercept), group, eyes, group * eyes[a]

a. Information criteria are in smaller-is-better form.

b. Computed using the full log quasi-likelihood function.

Tests of Model Effects

Source	Type III		
	Wald Chi-Square	df	Sig.
(Intercept)	874.869	1	.000
group	2.956	1	.086
eyes	4.373	1	.037
group * eyes	.597	1	.440

Dependent Variable: MTF53m
Model: (Intercept), group, eyes, group * eyes

Parameter Estimates

Parameter	B	Std. Error	95% Wald Confidence Interval		Hypothesis ...
			Lower	Upper	Wald Chi-Square
(Intercept)	.506	.0264	.454	.557	367.827
[group=1]	-.083	.0476	-.176	.011	3.024
[group=2]	0[a]	.	.	.	.
[eyes=1]	.042	.0438	-.043	.128	.942
[eyes=2]	0[a]	.	.	.	.
[group=1] * [eyes=1]	.050	.0645	-.077	.176	.597
[group=1] * [eyes=2]	0[a]	.	.	.	.
[group=2] * [eyes=1]	0[a]	.	.	.	.
[group=2] * [eyes=2]	0[a]	.	.	.	.
(Scale)	.012				

Parameter Estimates

Parameter	Hypothesis Test	
	df	Sig.
(Intercept)	1	.000
[group=1]	1	.082
[group=2]	.	.
[eyes=1]	1	.332
[eyes=2]	.	.
[group=1] * [eyes=1]	1	.440
[group=1] * [eyes=2]	.	.
[group=2] * [eyes=1]	.	.
[group=2] * [eyes=2]	.	.
(Scale)		

Dependent Variable: MTF53m
Model: (Intercept), group, eyes, group * eyes

a. Set to zero because this parameter is redundant.

```
 * Generalized Estimating Equations.
GENLIN PSF33m BY group eyes (ORDER=ASCENDING)
  /MODEL group eyes group*eyes INTERCEPT=YES
 DISTRIBUTION=NORMAL LINK=IDENTITY
  /CRITERIA SCALE=MLE PCONVERGE=1E-006(ABSOLUTE) SINGULAR=1E-012 ANALYSISTY
PE=3(WALD) CILEVEL=95
    LIKELIHOOD=FULL
  /REPEATED SUBJECT=patients WITHINSUBJECT=eyes SORT=YES CORRTYPE=INDEPENDE
NT ADJUSTCORR=YES
    COVB=ROBUST
  /MISSING CLASSMISSING=EXCLUDE
  /PRINT CPS DESCRIPTIVES MODELINFO FIT SUMMARY SOLUTION.
```

Generalized Linear Models

Notes

Output Created		29-NOV-2023 19:57...
Comments		
Input	Data	/Users/yangshan/Desktop/2022-5-12/ /2023-10-OBL/2023-11-22 revised manuscript/OBL .sav
	Active Dataset	DataSet1
	Filter	<none>
	Weight	<none>
	Split File	<none>
	N of Rows in Working Data File	56
Missing Value Handling	Definition of Missing	User-defined missing values for factor, subject and within-subject variables are treated as missing.
	Cases Used	Statistics are based on cases with valid data for all variables in the model.
Weight Handling		not applicable

Notes

Syntax		GENLIN PSF33m BY group eyes (ORDER=ASCENDING)<br>/MODEL group eyes group*eyes INTERCEPT=YES<br><br>DISTRIBUTION=NORMAL LINK=IDENTITY<br>/CRITERIA SCALE=MLE PCONVERGE=1E-006 (ABSOLUTE) SINGULAR=1E-012 ANALYSISTYPE=3(WALD) CILEVEL=95<br>LIKELIHOOD=FULL<br>/REPEATED SUBJECT=patients WITHINSUBJECT=eyes SORT=YES CORRTYPE=INDEPENDENT ADJUSTCORR=YES<br>COVB=ROBUST<br>/MISSING CLASSMISSING=EXCLUDE<br>/PRINT CPS DESCRIPTIVES MODELINFO FIT SUMMARY SOLUTION.
Resources	Processor Time	00:00:00.04
	Elapsed Time	00:00:00.00

Model Information

Dependent Variable		PSF33m
Probability Distribution		Normal
Link Function		Identity
Subject Effect	1	patients
Within-Subject Effect	1	eyes
Working Correlation Matrix Structure		Independent

Case Processing Summary

	N	Percent
Included	41	73.2%
Excluded	15	26.8%
Total	56	100.0%

Correlated Data Summary

Number of Levels	Subject Effect	patients	24
	Within-Subject Effect	eyes	2
Number of Subjects			24
Number of Measurements per Subject	Minimum		1
	Maximum		2
Correlation Matrix Dimension			2

Categorical Variable Information

			N	Percent
Factor	group	1	20	48.8%
		2	21	51.2%
		Total	41	100.0%
	eyes	1	24	58.5%
		2	17	41.5%
		Total	41	100.0%

Continuous Variable Information

		N	Minimum	Maximum	Mean	Std. Deviation
Dependent Variable	PSF33m	41	.02	.89	.1996	.24261

Goodness of Fit[a]

	Value
Quasi Likelihood under Independence Model Criterion (QIC)[b]	10.099
Corrected Quasi Likelihood under Independence Model Criterion (QICC)[b]	10.116

Dependent Variable: PSF33m
Model: (Intercept), group, eyes, group * eyes[a]

a. Information criteria are in smaller-is-better form.

b. Computed using the full log quasi-likelihood function.

Tests of Model Effects

Source	Type III		
	Wald Chi-Square	df	Sig.
(Intercept)	30.256	1	.000
group	3.457	1	.063
eyes	.696	1	.404
group * eyes	.115	1	.734

Dependent Variable: PSF33m
Model: (Intercept), group, eyes, group * eyes

Parameter Estimates

Parameter	B	Std. Error	95% Wald Confidence Interval		Hypothesis ...
			Lower	Upper	Wald Chi-Square
(Intercept)	.111	.0417	.030	.193	7.142
[group=1]	.107	.1067	-.102	.317	1.014
[group=2]	0[a]	.	.	.	.
[eyes=1]	.036	.0708	-.102	.175	.262
[eyes=2]	0[a]	.	.	.	.
[group=1] * [eyes=1]	.050	.1463	-.237	.336	.115
[group=1] * [eyes=2]	0[a]	.	.	.	.
[group=2] * [eyes=1]	0[a]	.	.	.	.
[group=2] * [eyes=2]	0[a]	.	.	.	.
(Scale)	.057				

Parameter Estimates

Parameter	Hypothesis Test	
	df	Sig.
(Intercept)	1	.008
[group=1]	1	.314
[group=2]	.	.
[eyes=1]	1	.609
[eyes=2]	.	.
[group=1] * [eyes=1]	1	.734
[group=1] * [eyes=2]	.	.
[group=2] * [eyes=1]	.	.
[group=2] * [eyes=2]	.	.
(Scale)		

Dependent Variable: PSF33m
Model: (Intercept), group, eyes, group * eyes

a. Set to zero because this parameter is redundant.

```
 * Generalized Estimating Equations.
GENLIN MTF33m BY group eyes (ORDER=ASCENDING)
  /MODEL group eyes group*eyes INTERCEPT=YES
 DISTRIBUTION=NORMAL LINK=IDENTITY
  /CRITERIA SCALE=MLE PCONVERGE=1E-006(ABSOLUTE) SINGULAR=1E-012 ANALYSISTY
PE=3(WALD) CILEVEL=95
    LIKELIHOOD=FULL
  /REPEATED SUBJECT=patients WITHINSUBJECT=eyes SORT=YES CORRTYPE=INDEPENDE
NT ADJUSTCORR=YES
    COVB=ROBUST
  /MISSING CLASSMISSING=EXCLUDE
  /PRINT CPS DESCRIPTIVES MODELINFO FIT SUMMARY SOLUTION.
```

Generalized Linear Models

Notes

Output Created		29-NOV-2023 19:57...
Comments		
Input	Data	/Users/yangshan/Desktop/2022-5-12/ /2023-10-OBL/2023-11-22 revised manuscript/OBL .sav
	Active Dataset	DataSet1
	Filter	<none>
	Weight	<none>
	Split File	<none>
	N of Rows in Working Data File	56
Missing Value Handling	Definition of Missing	User-defined missing values for factor, subject and within-subject variables are treated as missing.
	Cases Used	Statistics are based on cases with valid data for all variables in the model.
Weight Handling		not applicable

Notes

Syntax		GENLIN MTF33m BY group eyes (ORDER=ASCENDING)<br>/MODEL group eyes group*eyes INTERCEPT=YES<br><br>DISTRIBUTION=NORMAL LINK=IDENTITY<br>/CRITERIA SCALE=MLE PCONVERGE=1E-006 (ABSOLUTE) SINGULAR=1E-012 ANALYSISTYPE=3(WALD) CILEVEL=95<br>LIKELIHOOD=FULL<br>/REPEATED SUBJECT=patients WITHINSUBJECT=eyes SORT=YES CORRTYPE=INDEPENDENT ADJUSTCORR=YES<br>COVB=ROBUST<br>/MISSING CLASSMISSING=EXCLUDE<br>/PRINT CPS DESCRIPTIVES MODELINFO FIT SUMMARY SOLUTION.
Resources	Processor Time	00:00:00.04
	Elapsed Time	00:00:00.00

Model Information

Dependent Variable		MTF33m
Probability Distribution		Normal
Link Function		Identity
Subject Effect	1	patients
Within-Subject Effect	1	eyes
Working Correlation Matrix Structure		Independent

Case Processing Summary

	N	Percent
Included	40	71.4%
Excluded	16	28.6%
Total	56	100.0%

Correlated Data Summary

Number of Levels	Subject Effect	patients	24
	Within-Subject Effect	eyes	2
Number of Subjects			24
Number of Measurements per Subject	Minimum		1
	Maximum		2
Correlation Matrix Dimension			2

Categorical Variable Information

			N	Percent
Factor	group	1	20	50.0%
		2	20	50.0%
		Total	40	100.0%
	eyes	1	23	57.5%
		2	17	42.5%
		Total	40	100.0%

Continuous Variable Information

		N	Minimum	Maximum	Mean	Std. Deviation
Dependent Variable	MTF33m	40	.27	.68	.4718	.12555

Goodness of Fit[a]

	Value
Quasi Likelihood under Independence Model Criterion (QIC)[b]	8.675
Corrected Quasi Likelihood under Independence Model Criterion (QICC)[b]	8.587

Dependent Variable: MTF33m
Model: (Intercept), group, eyes, group * eyes[a]

a. Information criteria are in smaller-is-better form.

b. Computed using the full log quasi-likelihood function.

Tests of Model Effects

Source	Type III		
	Wald Chi-Square	df	Sig.
(Intercept)	521.838	1	.000
group	.359	1	.549
eyes	.910	1	.340
group * eyes	.487	1	.485

Dependent Variable: MTF33m
Model: (Intercept), group, eyes, group * eyes

Parameter Estimates

Parameter	B	Std. Error	95% Wald Confidence Interval		Hypothesis ...
			Lower	Upper	Wald Chi-Square
(Intercept)	.451	.0433	.367	.536	108.835
[group=1]	-.001	.0609	-.121	.118	.001
[group=2]	0[a]	.	.	.	.
[eyes=1]	.010	.0516	-.091	.111	.034
[eyes=2]	0[a]	.	.	.	.
[group=1] * [eyes=1]	.052	.0746	-.094	.198	.487
[group=1] * [eyes=2]	0[a]	.	.	.	.
[group=2] * [eyes=1]	0[a]	.	.	.	.
[group=2] * [eyes=2]	0[a]	.	.	.	.
(Scale)	.016				

Parameter Estimates

Parameter	Hypothesis Test	
	df	Sig.
(Intercept)	1	.000
[group=1]	1	.981
[group=2]	.	.
[eyes=1]	1	.853
[eyes=2]	.	.
[group=1] * [eyes=1]	1	.485
[group=1] * [eyes=2]	.	.
[group=2] * [eyes=1]	.	.
[group=2] * [eyes=2]	.	.
(Scale)		

Dependent Variable: MTF33m
Model: (Intercept), group, eyes, group * eyes

a. Set to zero because this parameter is redundant.
